# Supplementary figures and images for: Biological control under climate change: Distribution patterns of the South American fruit fly, Anastrepha fraterculus and two of its parasitoids in the Americas
Source: PLoS One. 2025 Jun 11;20(6):e0325761. doi: 10.1371/journal.pone.0325761 (PMC12157005; doi:10.1371/journal.pone.0325761)

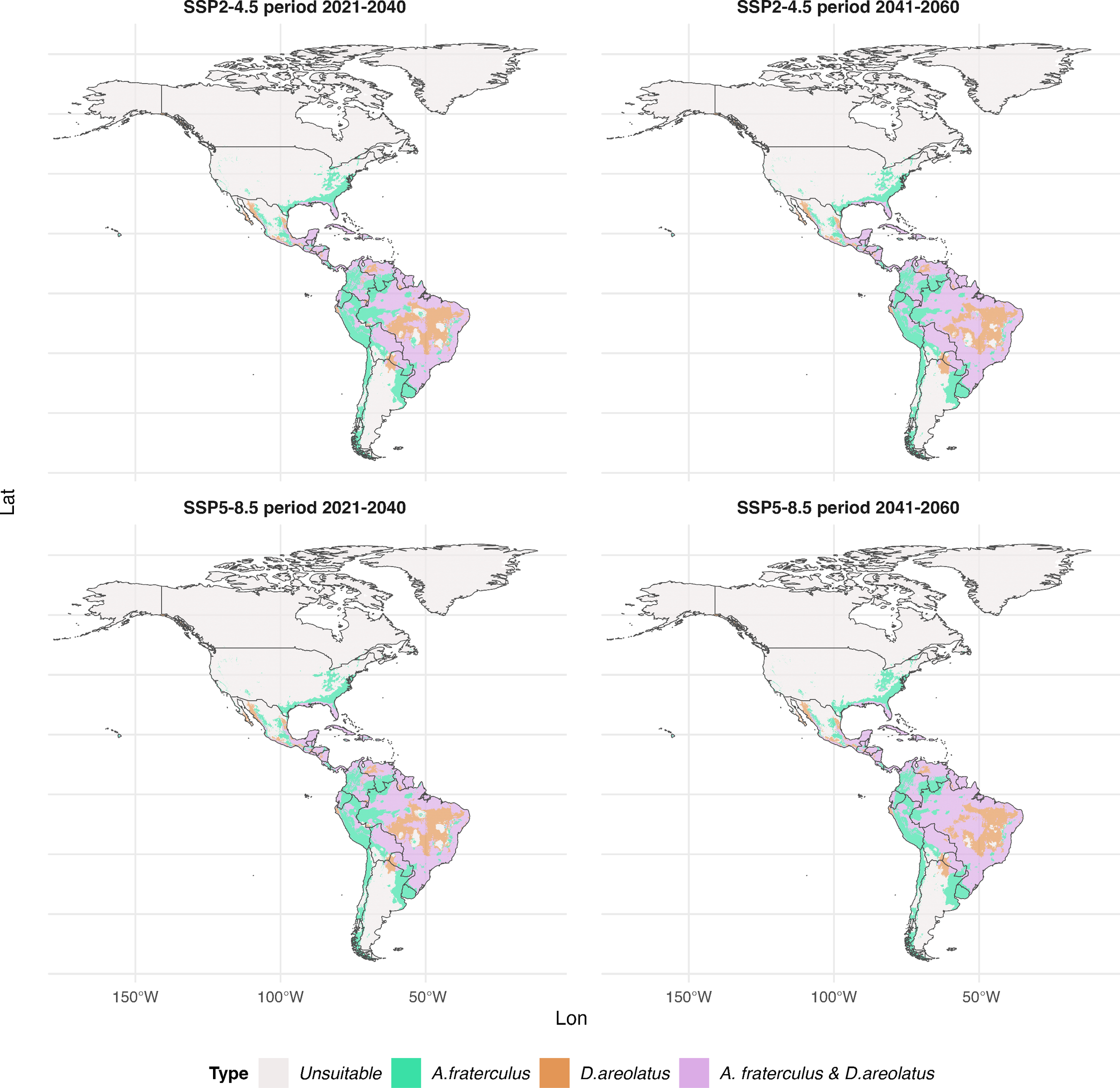

Supplement: S1 Fig — Overlapping suitable areas were predicted for A. fraterculus and D. areolatus under two future climatic scenarios (SSP2–4.5 and SSP5–8.5) and two time periods (2021–2040 and 2041–2060). (TIF) [file pone.0325761.s001.tif]

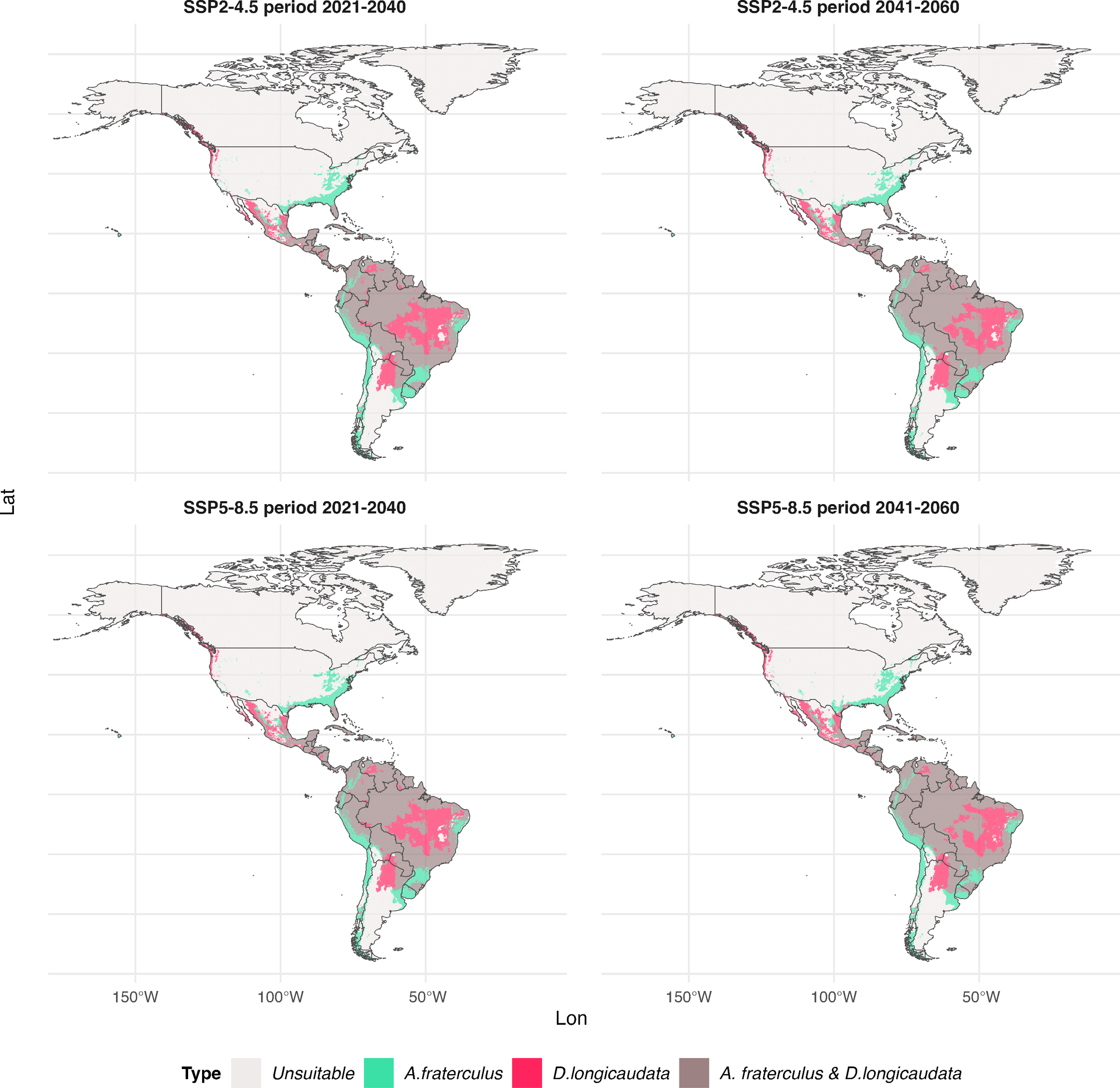

Supplement: S2 Fig — Overlapping suitable areas were predicted for A. fraterculus and D. longicaudata under two future climatic scenarios (SSP2–4.5 and SSP5–8.5) and two time periods (2021–2040 and 2041–2060). (TIF) [file pone.0325761.s002.tif]
