# Supplementary material for: Biological control under climate change: Distribution patterns of the South American fruit fly, Anastrepha fraterculus and two of its parasitoids in the Americas
Source: PLoS One. 2025 Jun 11;20(6):e0325761. doi: 10.1371/journal.pone.0325761 (PMC12157005; doi:10.1371/journal.pone.0325761)
Supplement: Attachment — Submitted filename: PONE-D-24-44826_R2.pdf [file pone.0325761.s007.pdf]

PLOS ONE

Biological control under climate change: Distribution patterns of the South American fruit fly, Anastrepha fraterculus and two of its parasitoids in the Americas

--Manuscript Draft--

**Manuscript Number:**	PONE-D-24-44826R2
**Article Type:**	Research Article
**Full Title:**	Biological control under climate change: Distribution patterns of the South American fruit fly, Anastrepha fraterculus and two of its parasitoids in the Americas
**Short Title:**	Distribution Patterns of the South American Fruit Fly and Two of its Parasitoids in the Americas
**Corresponding Author:**	Flor E. Acevedo, Ph.D.<br>Penn State College of Agricultural Sciences: The Pennsylvania State University College of Agricultural Sciences<br>University Park, PA UNITED STATES OF AMERICA
**Keywords:**	South American Fruit Flies; Climate change; Doryctobracon areolatus; Diachasmimorpha longicaudata; SDM; Biological control
**Abstract:**	Climate change affects the distribution of insects, such as pests and parasitoids. Species Distribution Models (SDMs) have been developed to determine distribution patterns and risk areas for pests and biological control agents under different climate change scenarios. The South American Fruit Fly, Anastrepha fraterculus (Wiedemann) (Diptera: Tephritidae), is an important pest of cultivated fruits throughout the Americas that can be controlled by natural enemies, such as the native parasitoid Doryctobracon areolatus (Szépligeti) (Hymenoptera: Braconidae) and the introduced parasitoid Diachasmimorpha longicaudata (Ashmead) (Hymenoptera: Braconidae). However, the control efficacy and parasitism performance of these organisms could be affected by changing environmental conditions. SDMs were conducted using Random Forest to predict suitable areas for the establishment of A. fraterculus, D. areolatus, and D. longicaudata under different climate scenarios or Representative Concentration Pathways (SSPs) (SSP 2-4.5 and 5-8.8) in two different periods (2021-2040 and 2041-2060). Our results predicted an increase in suitable areas for A.fraterculus in the Americas, especially in some South American countries such as Colombia and Brazil. Moreover, the projected distribution of these species is intricately linked to the regional climatic patterns. Where temperate and tropical areas were more suitable for the establishment of A. fraterculus, temperate climates were better for D. areolatus, and tropical climates were more suitable for D. longicaudata. Suitable areas for the establishment of both parasitoid species were predicted to increase in future climate scenarios, with D. longicaudata having a greater geographical expansion than D. areolatus. These parasitoids could be used as biocontrol agents in almost all areas suitable for the establishment of A. fraterculus.
**Order of Authors:**	Jesús H. Gómez-Llano
	Fabio L. Galvão Silva
	Flor E. Acevedo, Ph.D.
	Fabio Castro Llanos
	Marco Silva Gottschalk
	Dori Edson Nava
**Opposed Reviewers:**	
**Response to Reviewers:**	Response to reviewers<br><br>Editor comments:<br>Thank you for submitting your manuscript to PLOS ONE. After careful consideration, we feel that it has merit but does not fully meet PLOS ONE's publication criteria as it currently stands. Therefore, we invite you to submit a revised version of the manuscript that addresses the points raised during the review process.

This is a much improved version, however, the ms still needs additional improvements. Please careful edit your ms to remove grammatical error and improve other parts (e.g. quotation of references cited). Comments of both reviewers should be carefully considered and addressed. Authors may also consider to further elaborate either on maps or in the text on the expected fruit crops that would come under attack due to climate change.

Reply: We appreciate the editor's observations. The grammatical errors in the manuscript have been corrected, and the references have been revised to comply with the journal's requirements. Regarding the fruit crops potentially affected by the expansion of A. fraterculus, we mentioned some of these crops in the manuscript. However, we acknowledge that this pest has over 160 host species. To address this, we have added a paragraph at the end of the first section of the discussion, emphasizing the importance of focusing research on crops in high-risk regions.

Review Comments to the Author:

1.Reviewer #1: General comments:

The manuscript has been improved but grammatical errors remain at the following lines and should be corrected before publication.

Line 50- should be "Therefore, increases in mean temperature, fewer occurrences of freezing temperatures"

Response: The sentence was corrected (line 52)

Line 68- should be "This approach may help entomologists to predict".

Response: The sentence was corrected (line 71)

Line 79- "MASL (meters above sea level)".

Response: The sentence was corrected (line 83)

Line 110 delete "in the future"

Response: The sentence was corrected (line 123)

Line 398 delete "While" or use a comma instead of a period.

Response: The sentence was corrected (line 446)

Line 41 possibly should be "in the U.S. and Mexico"

Response: Maybe the Line wasn't written right by the reviewer, because do not match the line 41 with the suggestion.

Line 533 delete While" as in line 398.

Response: The sentence was corrected (line 623)

Line 581 "Florida. The parasitoid reduced"

Response: The sentence was corrected (line 690)

We sincerely appreciate the time and careful attention put into reviewing our manuscript. We have implemented all the grammatical corrections you identified,

2.Reviewer #3: General comments:

The manuscript entitled "Biological control under climate change: Distribution patterns of the South American fruit fly, Anastrepha fraterculus and two of its parasitoids in the Americas" uses species distribution models (SDM) to predict the potential geographic range of a pest, its biological control agents, and the areas that overlap between the two.

The manuscript is clear and acceptable for publication. Here are some minor suggestions or comments:

Introduction comments: The three hypotheses (L113 – 119) are interesting but are never discussed further.

Reply: In response to the reviewer's comment, we have added dedicated discussion paragraphs after each relevant section to explicitly address and evaluate the three hypotheses. See lines 525-534, 609-617, and 704-709.

Materials and methods comments: (L123-130) How many occurrences per species were found and used to build the SDM analysis? Are you sure you can find

Powered by Editorial Manager® and ProduXion Manager® from Aries Systems Corporation

	occurrences of all three species in the Vertebrate Network? Have you sorted or selected the occurrences (according to their reliability, for example)?<br>Reply: The number of occurrences used in the model's construction has been added to the methods section (lines 143-148). Regarding the vertebrate network database, no records were initially available for the species in this database. To obtain these records, we used the "occ" function from the "spocc" package in RStudio, following the code provided by its authors, and included all recommended databases. The occurrences were then filtered to remove duplicates. Due to the limited number of available records for the parasitoids, we retained all occurrences in the final model construction.<br><br>Results comments: In general: Occupation zones are described by countries, regions (Amazon region, Mesoamerican countries, Orinoquia region....) and geological formations (Andes mountains). These names are never indicated on any map, which makes them difficult for non-specialist readers to understand.<br>Reply: We appreciate the suggestion. While we did not label specific zones (e.g., countries, regions like the Amazon or Orinoquía, or geological formations like the Andes) on the maps, we intentionally described them using cardinal directions (e.g., northern, southern) within each country or area. This approach was taken to emphasize the significance of these regions in relation to pest expansion and to guide research focus accordingly. However, we acknowledge that adding geographical labels could improve clarity for non-specialist readers, and we will consider this for future work.<br><br>Other comments: L247-251: "the largest extension of suitable areas". This paragraph requires clarification. You mention "largest extension of suitable areas", but it is unclear whether the values refer to current or future occupancy or an extension factor. From Table 2, it looks like occupancy between 2041 and 2060 according to the SS95-8.5 scenario, although not all the values are identical.<br>Reply: The paragraph has been modified to specify that the values refer to projected occupancy under the SSP5-8.5 scenario (2041-2060). See lines 273-277<br><br>Fig 1 legend: L291: Remove "Extension of".<br>Reply: "Extension of" has been removed from the Fig 1 legend (Line 330)<br><br>Fig 2 legend: L297: Change "(B-D)" by (B-E). Idem for fig 3 and fig 5.<br>The figure references have been corrected to (B-E) in Figs 2, 3, and 5 legends. (lines 336, 370, 429).<br><br>Discussion comments: The three main parts of the discussion contain a substantial amount of information. It may be advisable to conclude each section with a summary of the key points (a take-home message).<br>Reply: We appreciate the reviewer's suggestion. In response, we have added concise summary statements at the end of each discussion section to highlight the key findings and main conclusions of our study.<br><br>Bibliography comments: In some refs, each word is capitalized. Species names are not always italicized.<br>Reply: Regarding our bibliography formatting, we have systematically addressed these issues by:<br>•Standardizing title capitalization to sentence case for all references<br>•Ensuring proper italicization of all species names throughout the bibliography<br>These corrections have been implemented across the entire reference section to comply with the journal's style guidelines and maintain proper formatting standards.<br><br>We extend our sincere gratitude to the reviewers for their insightful feedback, which has been important in refining this work. We deeply appreciate the careful attention given to our manuscript; each constructive comment has helped strengthen our arguments and enhance clarity. The manuscript has significantly improved through the revision process, and we are truly thankful for the expertise shared throughout the review.
**Additional Information:**	
**Question**	**Response**

**Financial Disclosure**<br><br>Enter a financial disclosure statement that describes the sources of funding for the work included in this submission. Review the submission guidelines for detailed requirements. View published research articles from *PLOS ONE* for specific examples.<br><br>This statement is required for submission and **will appear in the published article** if the submission is accepted. Please make sure it is accurate.<br><br>**Funded studies**<br>Enter a statement with the following details:<br>• Initials of the authors who received each award<br>• Grant numbers awarded to each author<br>• The full name of each funder<br>• URL of each funder website<br>• Did the sponsors or funders play any role in the study design, data collection and analysis, decision to publish, or preparation of the manuscript?<br><br>Did you receive funding for this work?	Yes
Please add funding details.<br>as follow-up to "**Financial Disclosure**<br><br>Enter a financial disclosure statement that describes the sources of funding for the work included in this submission. Review the submission guidelines for detailed requirements. View published research articles from *PLOS ONE* for specific examples.<br><br>This statement is required for submission and **will appear in the published article** if the submission is accepted. Please make sure it is accurate.	The author J. H. Gomez received financial support from the Coordination for the Improvement of Higher Education Personnel (CAPES) – Financing Code 001 for the development of this study. The author D. E. Nava received financial support from the National Council for Scientific and Technological Development (CNPq), Grant #310233/2020-8, for the development of this study

**Funded studies**<br>Enter a statement with the following details:<br>• Initials of the authors who received each award<br>• Grant numbers awarded to each author<br>• The full name of each funder<br>• URL of each funder website<br>• Did the sponsors or funders play any role in the study design, data collection and analysis, decision to publish, or preparation of the manuscript?<br><br>Did you receive funding for this work?"	
Please select the country of your main research funder (please select carefully as in some cases this is used in fee calculation).<br>as follow-up to "**Financial Disclosure**<br><br>Enter a financial disclosure statement that describes the sources of funding for the work included in this submission. Review the submission guidelines for detailed requirements. View published research articles from *PLOS ONE* for specific examples.<br><br>This statement is required for submission and **will appear in the published article** if the submission is accepted. Please make sure it is accurate.<br><br>**Funded studies**<br>Enter a statement with the following details:<br>• Initials of the authors who received each award<br>• Grant numbers awarded to each author<br>• The full name of each funder<br>• URL of each funder website<br>• Did the sponsors or funders play any role in the study design, data collection and analysis, decision to publish, or preparation of the manuscript?<br><br>Did you receive funding for this work?"	BRAZIL - BR
**Competing Interests**	The authors have declared that no competing interests exist

Use the instructions below to enter a competing interest statement for this submission. On behalf of all authors, disclose any competing interests that could be perceived to bias this work—acknowledging all financial support and any other relevant financial or non-financial competing interests.<br><br>This statement is required for submission and **will appear in the published article** if the submission is accepted. Please make sure it is accurate and that any funding sources listed in your Funding Information later in the submission form are also declared in your Financial Disclosure statement.<br><br>View published research articles from *PLOS ONE* for specific examples.<br><br>**NO authors have competing interests**<br><br>Enter: *The authors have declared that no competing interests exist.*<br><br>**Authors with competing interests**<br><br>Enter competing interest details beginning with this statement:<br><br>*I have read the journal's policy and the authors of this manuscript have the following competing interests: [insert competing interests here]*<br><br>* typeset	
**Ethics Statement**<br><br>Enter an ethics statement for this submission. This statement is required if the study involved:<br><br>• Human participants<br>• Human specimens or tissue<br>• Vertebrate animals or cephalopods<br>• Vertebrate embryos or tissues	N/A

• Field research<br><br>Write "N/A" if the submission does not require an ethics statement.<br><br>General guidance is provided below. Consult the submission guidelines for detailed instructions. **Make sure that all information entered here is included in the Methods section of the manuscript.**	

Powered by Editorial Manager® and ProduXion Manager® from Aries Systems Corporation

**Format for specific study types**<br><br>**Human Subject Research (involving human participants and/or tissue)**<br>• Give the name of the institutional review board or ethics committee that approved the study<br>• Include the approval number and/or a statement indicating approval of this research<br>• Indicate the form of consent obtained (written/oral) or the reason that consent was not obtained (e.g. the data were analyzed anonymously)<br><br>**Animal Research (involving vertebrate animals, embryos or tissues)**<br>• Provide the name of the Institutional Animal Care and Use Committee (IACUC) or other relevant ethics board that reviewed the study protocol, and indicate whether they approved this research or granted a formal waiver of ethical approval<br>• Include an approval number if one was obtained<br>• If the study involved *non-human primates*, add *additional details* about animal welfare and steps taken to ameliorate suffering<br>• If anesthesia, euthanasia, or any kind of animal sacrifice is part of the study, include briefly which substances and/or methods were applied<br><br>**Field Research**<br><br>Include the following details if this study involves the collection of plant, animal, or other materials from a natural setting:<br>• Field permit number<br>• Name of the institution or relevant body that granted permission	
**Data Availability**<br><br>Authors are required to make all data underlying the findings described fully available, without restriction, and from the time of publication. PLOS allows rare exceptions to address legal and ethical concerns. See the PLOS Data Policy and FAQ for detailed information.	Yes - all data are fully available without restriction

A Data Availability Statement describing where the data can be found is required at submission. Your answers to this question constitute the Data Availability Statement and **will be published in the article**, if accepted.<br><br>**Important:** Stating 'data available on request from the author' is not sufficient. If your data are only available upon request, select 'No' for the first question and explain your exceptional situation in the text box.<br><br>Do the authors confirm that all data underlying the findings described in their manuscript are fully available without restriction?	
**Describe where the data may be found in full sentences. If you are copying our sample text, replace any instances of XXX with the appropriate details.**<br><br>• If the data are **held or will be held in a public repository**, include URLs, accession numbers or DOIs. If this information will only be available after acceptance, indicate this by ticking the box below. For example: *All XXX files are available from the XXX database (accession number(s) XXX, XXX.*).<br>• If the data are all contained **within the manuscript and/or Supporting Information files**, enter the following: *All relevant data are within the manuscript and its Supporting Information files.*<br>• If neither of these applies but you are able to provide **details of access elsewhere**, with or without limitations, please do so. For example:<br><br>*Data cannot be shared publicly because of [XXX]. Data are available from the XXX Institutional Data Access / Ethics Committee (contact via XXX) for researchers who meet the criteria for access to confidential data.*<br><br>*The data underlying the results presented in the study are available from (include the name of the third party*	All relevant data are within the manuscript and its Supporting Information files. The raw data are available at https://doi.org/10.5281/zenodo.15299850<br>in the Zenodo repositoy

Powered by Editorial Manager® and ProduXion Manager® from Aries Systems Corporation

and contact information or URL).<br>• This text is appropriate if the data are owned by a third party and authors do not have permission to share the data.<br><br>* typeset	
Additional data availability information:	Tick here if the URLs/accession numbers/DOIs will be available only after acceptance of the manuscript for publication so that we can ensure their inclusion before publication.

Brazil RS, Jan 1, 2025

Nikos T. Papadopoulos
Academic Editor
PlOS ONE

We are pleased to re-submit the attached manuscript titled: **"Biological Control under Climate Change: Distribution Patterns of the South American Fruit Fly, *Anastrepha fraterculus* and Two of its Parasitoids in the Americas"** for your consideration**.** Climate change is causing widespread global impacts, altering environmental conditions and affecting species distribution patterns. The South American fruit fly, *Anastrepha fraterculus*, is one of the major pests of cultivated fruits, with a distribution range extending from south of Texas to Argentina. Currently, fruit fly management in the Americas involves the use of native parasitoids such as *Dorytobraconn areolatus* and exotic parasitoids like *Diachamismorpha longicaudata*. However, there is a lack of knowledge regarding the response of these parasitoids to Climate change. Understanding how climate change affects the distribution patterns of both, pests and their parasitoids, is essential for developing effective pest management strategies. In this study, we use Species Distribution Models (SDMS) to determine suitable areas for *A. fraterculus* and its parasitoids *D. areolatus* and *D longicaudata*, assessing both current and future time predictions in the Americas. The future climate predictions were made in two periods of time 2021-2041 and 2041-2060 under two different climate change scenarios: a favorable scenario (SSP2-4.5) and the worst scenario (SSP5-8.5). Our findings indicate that suitable areas for *A. fraterculus* could increase in future all climate change scenarios, posing substantial challenges in South American countries such as Brazil and Colombia. This expansion could lead to greater economic losses for fruit crops in these regions. Additionally, our predictions suggest that suitable areas for both parasitoids' species could increase in the future, with tropical climate regions being more suitable for *D. longicaudata* and temperate climate regions being more suitable for *D. areolatus*.
This study also provides useful knowledge for early detections and rapid response strategies for *A. fraterculus* by predicting new suitable areas under climate change in the Americas. Furthermore, the knowledge gained from this study can guide future biological control programs involving the release of *D. areolatus* and *D. longicaudata* in the Americas. As a result, this study is adding novel information for fruit fly biological control in the context of climate change.

We are grateful to the reviewers for their valuable suggestions. Additionally, I would like to request assistance in modifying my Funding Statement with the following paragraph: "The author J. H. Gomez received financial support from the Coordination for the Improvement of Higher Education Personnel (CAPES) – Financing Code 001 for the development of this study. The author D. E. Nava received financial support from the National Council for Scientific and Technological Development (CNPq), Grant #310233/2020-8, for the development of this study". Also, I request help to know if there is any possibility of adding or change the author of the correspondence.

Thank you for your attention. We look forward to your comments.

Jesus Hernando Gomez Llano: jesushernandogll@gmail.com

manuscript Click here to access/download;Manuscript;Manuscript .docx

Biological control under climate change: Distribution patterns of the South American fruit fly, *Anastrepha fraterculus* and two of its parasitoids in the Americas

Jesús Hernando Gómez Llano[1]*, Fábio L. Galvão-Silva[1], Flor Edith Acevedo[2*], Fabio Castro Llanos[3], Marco Silva Gottschalk[4], Dori Edson Nava[5]

[1]Universidade Federal de Pelotas (UFPEL), Av. Eliseu Maciel – Campus Universitário Capão do Leão. Capão do Leão, Rio Grande do Sul, Brazil.

[2]Department of Entomology, The Pennsylvania State University, University Park, PA, United States

[3]International Center for Tropical Agriculture (CIAT), Valle del Cauca, Colombia.

[4]Departamento de Ecologia, Zoologia e Genética, Universidade Federal de Pelotas (UFPel), Capão do Leão, Rio Grande do Sul, Brazil.

[5]Embrapa Clima Temperado, Pelotas, Rio Grande do Sul, Brazil.

* Corresponding authors:

E-mail: jesushernandogll@gmail.com , fea5007@psu.edu

Abstract

Climate change affects the distribution of insects, such as pests and parasitoids. Species Distribution Models (SDMs) have been developed to determine distribution patterns and risk areas for pests and biological control agents under different climate change scenarios. The South American Fruit Fly, *Anastrepha fraterculus* (Wiedemann) (Diptera: Tephritidae), is an important pest of cultivated fruits throughout the Americas that can be controlled by natural enemies, such as the native parasitoid *Doryctobracon areolatus* (Szépligeti) (Hymenoptera: Braconidae) and the introduced parasitoid *Diachasmimorpha longicaudata* (Ashmead) (Hymenoptera: Braconidae). However, the control efficacy and parasitism performance of these organisms could be affected by changing environmental conditions. SDMs were conducted using Random Forest to predict suitable areas for the establishment of *A. fraterculus*, *D. areolatus*, and *D. longicaudata* under different climate scenarios or Representative Concentration Pathways (SSPs) (SSP 2-4.5 and 5-8.8) in two different periods (2021-2040 and 2041-2060). Our results predicted an increase in suitable areas for *A. fraterculus* in the Americas, especially in some South American countries such as Colombia and Brazil. Moreover, the projected distribution of these species is intricately linked to the regional climatic patterns. Where temperate and tropical areas were more suitable for the establishment of *A. fraterculus,* temperate climates were better for *D. areolatus*, and tropical climates were more suitable for *D. longicaudata.* Suitable areas for the establishment of both parasitoid species were predicted to increase in future climate scenarios, with *D. longicaudata* having a greater geographical expansion than *D. areolatus.* These parasitoids could be used as biocontrol agents in almost all areas suitable for the establishment of *A. fraterculus*.

Keywords: South American Fruit Flies; Climate change; *Doryctobracon areolatus*; *Diachasmimorpha longicaudata*; SDM; Biological control.

Introduction

Climate change and extreme weather conditions are consequences of increasing concentrations of greenhouse gases in the atmosphere from human activity [1,2]. Therefore, increases in mean temperature, fewer occurrences of freezing temperatures, and increases in extreme precipitation are expected in some areas of the planet [3]. These phenomena affect not only the weather but also the distribution of species, including insect pests and their natural enemies. Increasing temperatures can directly affect insect reproduction, development, survival, and dispersal patterns [4]. Climate change is predicted to have a greater impact on insect natural enemies, such as parasitoids, than on insect pests themselves. The limited plasticity of natural enemies at high temperatures causes temporal desynchronization with their hosts and decreases suitable distribution areas for parasitoids [5–7]. Although increasing temperatures often increase parasitism rates to a certain level, after the optimum peak is reached, any increase in temperature reduces parasitism rates [8,9]. Therefore, climate change is critical to the success of biological control programs.

The recognized effect of climate change on the distribution of insects has led to the development of computational models for predicting pest risks in different areas [10]. These species distribution models (SDMs) use environmental variables and records of species occurrences to predict potentially suitable areas for species to grow and develop; these correlative models are commonly used to predict changes in biodiversity in various climate change scenarios

[11]. Currently, some SDMs are being developed using machine learning algorithms, such as MaxEnt, Bioclim, and Random Forest [12–16]. The goal of these SDMs is to predict suitable areas for species in the current and future climate scenarios. This approach may help entomologists to predict the effects of climate change on agricultural pests and their natural enemies and can be used as a tool to determine appropriate areas for the release of parasitoids for pest mitigation [17–19].

The South American fruit fly *Anastrepha fraterculus* (Wiedemann, 1830) (Diptera: Tephritidae) is one of the most significant pests in the Americas and is distributed from Mexico to Argentina [20]. Currently, this species exhibit eight distinct morphotypes, as defined by Hernández-Ortiz et al. [21–23]: Andean, Brazilian-1, Brazilian-2, Brazilian-3, Ecuadorian, Mexican, Peruvian, and Venezuelan. The fruit fly *A. fraterculus* feeds on at least 160 crop species [24,25]. In Brazil, it is considered one of the primary pests affecting apple (*Malus domestica* Borkh.), peach [*Prunus persica* (L.) Bastch], and guava (*Psidium guajava* L.) [25,26]. In Colombia, *A. fraterculus* is a quarantine species reported feeding on coffee berries (*Coffea arabica* L.) in the Andean mountains at up to 1.100 MASL (meters above sea level) [27–29]. In Peru, this species is common in mango crops (*Magnifera indica* L.) [30,31]. In Mexico, this pest is particularly problematic for mango crops, which is a major agricultural product in many areas of the country [32].

One of the most common methods for controlling *Anastrepha* fruit flies involves the use of classical, augmentative, and conservative biological control techniques, utilizing native and exotic parasitoids [32–34]. Some of the more prevalent parasitoid species of *Anastrepha* sp. are *Doryctobracon areolatus* (Szépligeti, 1911) (Hymenoptera: Braconidae), native to South America, and *Diachasmimorpha longicaudata* (Ashmead, 1905) (Hymenoptera: Braconidae), native to

Southeast Asia [35,36]. The parasitism rates of *D. areolatus* on *A. fraterculus* varied from 41.6% to 68.6% (53.5% on average), demonstrating its potential in biological control programs [37,38]. Similar, the parasitism rates of *D. longicaudata* on *A. fraterculus* ranged from 12.9% to 62.9% when reared on citrus and guava fruits in laboratory conditions and 35.5% in artificial diet [39,40], Recently, the Brazilian Ministry of Agriculture and Livestock (MAPA) has registered *D. areolatus* as a fruit fly biocontrol agent for *Anastrepha* spp. [41].

The parasitoid *D. longicaudata* has been introduced in different countries in the Americas, including Colombia, Venezuela, Brazil, Costa Rica, Guatemala, El Salvador, Mexico, Nicaragua, Trinidad, and the United States of America (Florida) as a biological control agent of many tephritids [42,43]. This parasitoid has been found parasitizing *A. fraterculus* in Costa Rica, Mexico, Nicaragua, Guatemala, El Salvador, Trinidad, Colombia, and Venezuela [42]. Furthermore, augmentative releases of *D. longicaudata* in Mexico have resulted in up to 50% parasitism of *Ceratitis capitata* (Wiedemann, 1824) [44]. This parasitoid was also introduced to Argentina in the 1960s and 1990s to control *C. capitata* and *A. fraterculus* [45]. In Brazil, it was introduced in 1994 and officially registered as a biocontrol agent of tephritids in 2018 [33,46]. However, despite some releases in the states of Bahia and São Paulo in Brazil, where the parasitism rates reached values of 19.8% in citrus orchards [47], the parasitoid has not been successfully established speculatively due to intraspecific competition with native parasitoid species. *D. longicaudata* also failed to establish in the state of Rio Grande do Sul due to low temperatures in the region [43,48]. These studies suggest that *D. areolatus* and *D. longicaudata* require different ecological conditions for their successful establishment.

This study predicts the potential geographic distribution of the fruit fly *A. fraterculus* and its parasitoids *D. areolatus* and *D. longicaudata* across the Americas under current and future climate

conditions. Additionally, this study forecasts potential population range shifts using a comprehensive set of climate projection models, considering two shared socioeconomic pathways of gas emissions (SSP) and two distinct periods of time. We tested the following hypotheses: 1) the suitable areas for the establishment of the fruit fly *A. fraterculus* will increase in the future; 2) the suitable areas for the establishment of the parasitoids *D. longicaudata* and *D. areolatus* will not increase in the future*;* and 3) there will be less suitable areas for the introduced parasitoid *D. longicaudata* than for the native parasitoid *D. fraterculus* in future climate change scenarios. The results of this study contribute to the development of pest management programs for *A. fraterculus* by determining potential areas for the establishment of this pest and two of its parasitoid species in the Americas.

Materials and methods

Species record occurrences

The geographical coordinates of species occurrences used to build the SDM analysis for *A. fraterculus*, *D. longicaudata*, and *D. areolatus* were obtained using the "occ" function from the "spocc" RStudio package [49]. The occurrence records of these species were sourced from the following databases: The Global Biodiversity Information Facility (GBIF; https://www.gbif.org), The Vertebrate Network (VerNet, http://vertnet.org/index.html), iNaturalist (https://www.inaturalist.org/), and The Integrated Digitized Biocollections (iDigBio). Additional occurrences of *D. areolatus* were extracted from the records reported by Moreira et al. [50]. After filtering the data to remove duplicates, we obtained a total of 193 records for *A. fraterculus*, 21 for

D. longicaudata, and 205 for *D. areolatus*. The georeferenced occurrences for each species were then compiled and saved in a ".csv" file for subsequent analysis.

Both occurrences and absences are usually necessary to build SDMs. Nevertheless, due to the lack of true absence data in the databases, "pseudo-absence" locations were generated randomly to characterize the environmental background of the study area. To do this, we used the function "randomPoints" from the "dismo" package [51] in R statistical computing software v4.3.2; [50]. These pseudo-absences are locations where it is assumed that there is no record or presence of the species, thus providing a point of comparison from those locations where the species has been recorded. In this study, a ratio of 2:1 pseudo-absence to presence locations was generated for each species, reducing over-fitting and maintaining the capacity of the model to discriminate between areas of presence and absence [53].

Environmental variables

SDMs use data on environmental conditions in which a species is known to exist to predict suitable habitats for that species. Environmental data for current and future climate conditions in the Americas were obtained from the WorldClim database (Global Climate Data; available at: https://www.worldclim.org/data/index.html) [54]. These data consist of 19 bioclimatic variables derived from temperature and precipitation in "raster" format (Table 1), at a spatial resolution of 2.5 arc-minutes (approximately 4.5 km).

Table 1. Bioclimatic variables.

Code	Variables

BIO1	Annual Mean Temperature
BIO2	Mean Diurnal Range (Mean of monthly (max temp - min temp))
BIO3	Isothermality (BIO2/BIO7) (×100)
BIO4	Temperature Seasonality (standard deviation ×100)
BIO5	Max Temperature of Warmest Month
BIO6	Min Temperature of Coldest Month
BIO7	Temperature Annual Range (BIO5-BIO6)
BIO8	Mean Temperature of Wettest Quarter
BIO9	Mean Temperature of Driest Quarter
BIO10	Mean Temperature of Warmest Quarter
BIO11	Mean Temperature of Coldest Quarter
BIO12	Annual Precipitation
BIO13	Precipitation of Wettest Month
BIO14	Precipitation of Driest Month
BIO15	Precipitation Seasonality (Coefficient of Variation)
BIO16	Precipitation of Wettest Quarter
BIO17	Precipitation of Driest Quarter
BIO18	Precipitation of Warmest Quarter
BIO19	Precipitation of Coldest Quarter

Bioclimatic variables used for SDM (extracted from www.worldclim.org/data/bioclim.html**)**.

The current environmental conditions were downloaded from the WorldClim database version 2.1 and correspond to historical records from 1970 to 2000 [54]. The environmental conditions for future climate scenarios were obtained from 26 climatic projections or Global Climate Models (GCM) from the Coupled Model Intercomparison Project Phase 6 (CMIP6; available at https://www.worldclim.org/data/cmip6/cmip6climate.html, 52) (S1 Table). These projections consist of climatic predictions made by various modeling groups based on CO_2 emissions and mitigation effort scenarios up to the year 2100, called Shared Socioeconomic Pathways (SSPs). We selected two SSPs at two time periods, from 2021 to 2040 and from 2041 to 2060: (a) SSP2-4.5, which represents a stabilization scenario that assumes the implementation of climatic policies to mitigate greenhouse gas emissions [56]; and (b) SSP5-8.5, which corresponds

to high greenhouse gas emissions pathway and is considered a baseline scenario without any specific targets for climate mitigation [57].

Suppression of collinearity in predictor variables

Collinearity, also known as multicollinearity, refers to the strong interdependence among explanatory variables typically observed in regression models. This phenomenon can lead to two primary issues: inflated estimates of variable effects and uncertainty in model extrapolation [58]. The most common approach for addressing collinearity issues is a priori variable selection, which involves either excluding the most correlated variables or combining them into new explanatory terms [59]. For this purpose, each species occurrence datum was projected into the current environmental conditions database, and the values of each bioclimatic variable at those points were extracted. Subsequently, the Variance Inflation Factor (VIF) was calculated using the "vifstep" function from the "usdm" package [60]. This test excluded the highly correlated variables with VIF values >10; the remaining variables were used to run the SDMs projections.

Modeling and projections

Random Forest (RF) is a powerful machine-learning method for building SDMs and training models with small sample sizes [61–63]. This method consists of hundreds of trees constructed using random samples from the databases of occurrences/pseudo-absences and selected bioclimatic variables (predictors). Each tree is built from a bootstrap sample, which is a random sample with replacement from the original dataset. At each node of the tree, the best predictor is selected from a small, randomly chosen subset of the predictor variable pool. This process helps introduce randomness and reduce overfitting of the model [64]. The final prediction

is then made by aggregating the predictions of all individual trees. This ensemble approach tends to improve the robustness and generalization performance of the model compared to the use of a single decision tree [65].

RF involves several parameters that control the structure of each tree, the overall structure and size of the forest, and its randomness properties. For example, the number of randomly drawn candidate variables (mtry), the node size, and the number of trees (ntree). The optimal selection of these parameters significantly influences model prediction accuracy [66]. To determine the best values for these parameters, strategies such as "Tuning" have been developed [67]. In this context, parameter selection was performed using the "train" function from the "caret" package [68]. For all predictions made for *A. fraterculus* and *D. areolatus*, a ntree of 2000, a mtry of 3, and a node size of 2 with 25 replications were selected. However, for all predictions involving *D. longicaudata*, a ntree of 2500, a mtry of 3, and a node size of 2, with 10 replications were selected. Subsequently, all the models were adjusted using 75% of the occurrence data to train the model and the remaining 25% for testing purposes. An ensemble was created using all replications for the current projection. For future projections, an ensemble was formed by combining all 25 Global Climate Models (GCMs) for each climatic period (2021-2040 and 2041-2060) and Representative Concentration Pathway (SSP).

SDMs using RF usually provide numerical predictions in the range of 0–1. However, to make the data more practical, it is essential to establish a threshold for interpreting the predictions using levels of suitability and transforming them into binary data indicating species presence or absence. Suitability scores higher than the threshold value are considered suitable areas for the establishment of a given species. In this study, threshold values were obtained for each insect species by extracting all the Receiver Operating Characteristic (ROC) curve data using the function

“roc” of the “pROC” package [69]. Subsequently, the threshold was determined as the mean of all the threshold values in each replication for each species (25 for *A. fraterculus* and *D. areolatus*, and 10 for *D. longicaudata*). We used four suitability levels represented on a color gradient from green to red on the plotted maps: a) highly suitable areas depicted in green color correspond to suitability values close to 1; b) moderately suitable areas illustrated in yellow designate suitability values close to 0.5; c) low suitable areas depicted in red show suitability values close to the threshold for each species (0.08 for *A. fraterculus*, 0.31 for *D. areolatus* and 0.15 for *D. longicaudata*); and d) unsuitable areas represented by the absence of color correspond to values below the threshold and close to 0.

Lastly, the suitability values for each predicted scenario and period were converted into binary values using a species-specific threshold. This transformation was conducted for two key purposes: 1) to quantify the suitable and unsuitable areas in square kilometers for all species, facilitating the assessment of expansion areas across different countries in the Americas; 2) to generate maps illustrating the overlap of suitable areas between each parasitoid and the pest *A. fraterculus*. For the first one, we utilized the "expanse" function from the "terra" package [70]. The resulting data were subsequently saved in a ".csv" file. To aid interpretation, the top 10 countries with the largest suitable areas for the fruit fly *A. fraterculus* were selected. The same countries were also chosen for quantifying the suitability areas of the parasitoids *D. areolatus* and *D. longicaudata*, and the data were visualized using a bar plot.

All the maps generated illustrating the suitable areas for each species, as well as the overlap of suitable areas between the parasitoids and pest, were generated using the function “ne_countries” of the “rnaturalearth” RStudio package [71].

Model performance

To assess the performance of the models, we used the Receiver Operating Characteristic (ROC) curve analysis, specifically examining the area under the curve (AUC) [72]. The AUC value is a widely accepted metric for evaluating the accuracy of SDMs. Generally, AUC values below 0.7 indicate poor performance, those between 0.7 and 0.9 suggest moderate performance, and values exceeding 0.9 indicate good performance [73]. For each species, the relative importance of the selected bioclimatic variables was determined through analysis of multicollinearity and AUC metrics. This comprehensive approach allowed us to gauge both the predictive power of the models and the significance of individual variables in the SDMs.

Results

The predicted SDMs constructed for *A. fraterculus*, *D. areolatus,* and *D. longicaudata*, had good performance with AUC values of 0.995 ± 0.005, 0.963± 0.034, and 0.9583 ± 0.1317 respectively. These models indicate that suitable areas for the pest *A. fraterculus* and the parasitoids *D. areolatus* and *D. longicaudata,* were predicted to increase in the Americas in all the scenarios and periods evaluated (Table 2). The species with the largest suitable areas in each prediction (SSPs scenario and periods) was *D. longicaudata*, estimated to occupy up to 38.06% of America's territory, followed by *A. fraterculus* with 36.17%, and *D. areolatus* with 29.66% (Table 2). The predicted occupancy areas had no marked differences between the climatic scenarios analyzed (Table 2).

Table 2. **Percentage of predicted suitable areas for the establishment of the pest *Anastrepha fraterculus*, and the parasitoids *Doryctobracon areolatus* and *Diachasmimorpha longicaudata* in the Americas in two Shared Socioeconomic Pathways (SSPs) at two periods.**

Species		SSP2-4.5		SSP5-8.5	
	Current	2021-2040	2041-2060	2021-2040	2041-2060
Anastrepha fraterculus	31.39%	34.56%	35.55%	34.58%	36.17%
Diachasmimorpha longicaudata	35.07%	36.66%	37.42%	36.79%	38.06%
Doryctobracon areolatus	21.53%	26.25%	28.37%	26.59%	29.66%

Among the bioclimatic variables analyzed, Isothermality (BIO3) had the highest contribution to the *A. fraterculus* SDM with 20.51%, followed by the mean temperature of the driest quarter (BIO9) with a contribution of 14.22%, and the precipitation of the warmest quarter (BIO18) with 13.52% (Table 3). For *D. areolatus* SDM, the bioclimatic variable with major contribution was the annual temperature range (BIO7) with 25.35%, followed by isothermality (BIO3) with a contribution of 16.20%, and precipitation of the wettest month (BIO13) with 12.05%. For *D. longicaudata*, two main variables contributed more than 70% to the SDM model: the average temperature of the coldest quarter (BIO11) with 51.02% and the precipitation of the wettest quarter (BIO18) with 27.33% (Table 3).

Table 3. The relative contribution of the bioclimatic variables for Random Forest models (RF) built for *Anastrepha fraterculus*, *Doryctobracon areolatus*, and *Diachasmimorpha longicaudata*.

Species	Codes	Bioclimatic variables	Contribution (%)
Anastrepha fraterculus	BIO2	Mean Diurnal Range	10.02
	BIO3	Isothermality	20.51
	BIO8	Mean Temperature of Wettest Quarter	11.02
	BIO9	Mean Temperature of Driest Quarter	14.22
	BIO13	Precipitation of Wettest Month	7.70
	BIO15	Precipitation Seasonality	11.54
	BIO18	Precipitation of Warmest Quarter	13.52
	BIO19	Precipitation of Coldest Quarter	11.45
Doryctobracon areolatus	BIO3	Isothermally	16.20
	BIO7	Temperature Annual Range	25.35
	BIO8	Mean Temperature of Wettest Quarter	8.22
	BIO10	Mean Temperature of Warmest Quarter	9.88
	BIO13	Precipitation of Wettest Month	12.05
	BIO14	Precipitation of Driest Month	6.04
	BIO15	Precipitation Seasonality	4.57
	BIO18	Precipitation of Warmest Quarter	10.79
	BIO19	Precipitation of Coldest Quarter	6.89
Diac hasm	BIO 11	Mean Temperature of Coldest Quarter	52.02

BIO15	Precipitation Seasonality	4.36
BIO16	Precipitation of Wettest Quarter	27.33
BIO18	Precipitation of Warmest Quarter	11.83
BIO19	Precipitation of Coldest Quarter	4.46

The *Anastrepha fraterculus* SDM

Our models predict that *A. fraterculus* has an extensive suitable area for establishment in South America, with Brazil exhibiting the largest extent (up to 5 million km^2), followed by Peru (up to 1 million km^2) and Colombia (up to 0.8 million km^2) (Fig 1). In Brazil, the current time projected suitable areas occupy 61% of the national territory, with a potential increase of up to 75% under future climate scenarios (S1 Table). These predicted suitable areas were predominant in the southern states (Rio Grande do Sul, Santa Catarina, Paraná), southeastern states (São Paulo, Minas Gerais, Rio de Janeiro, Espírito Santo), and parts of Bahia State in Brazil (Fig 2). In Peru, the projected suitable areas for *A. fraterculus* extend up to 98% of the country, with minimal variation in future projections (S1 Table), these suitable areas were predicted in the Andean mountains and adjacent valleys (Fig 2). Conversely, Colombia is projected to experience a significant expansion of suitable areas, from 70% to 99% of its territory under future scenarios (Fig 1), with new suitable areas emerging in the Plains (East Colombia) and Amazon (South Colombia) regions, showing moderate to low suitability for *A. fraterculus* (Fig 2).

Fig 1. Top 10 countries with the largest suitable areas (Km^2) for *Anastrepha fraterculus*. Predicted suitable areas for the parasitoids *Diachasmimorpha longicaudata* and *Doryctobracon areolatus* and the pest *A. fraterculus* in current and future time predictions (SSP2-4.5, SSP5-8.5) (2021-2040, 2041-2060.

Fig 2. Suitable areas predicted for *Anastrepha fraterculus* in the Americas. (A). Suitable areas predicted in the current time period*;* (B-E). Suitable areas predicted under two future climate change scenarios (SSP2-4.5 and SSP5-8.5) (periods 2021-2040 and 2041-2060) ;(B) scenario SSP2-4.5 time period 2021-2060; (C) scenario SSP2-4.5 time period 2041-2060; (D) scenario SSP5-8.5 time period 2021-2040; (E) scenario SSP5-8.5 time period 2041-2060. The maps show suitability values in a green to-red color where: high suitable areas depicted in green color correspond to suitability values close to 1; moderately suitable areas illustrated in yellow designate suitability values close to 0.5; low suitable areas depicted in red show suitability values close to the threshold the species (0.08 ~~for *A. fraterculus*~~); unsuitable areas represented by the absence of color correspond to values below the threshold and close to 0.

In North America, current time predictions indicate suitable areas for *A. fraterculus* along the east coast of the United States (0.9 million km^2) and in Mexico (0.8 million km^2) (Fig 1), particularly in the coastal states of Yucatán, Campeche, Chiapas, Oaxaca, and Tabasco. In the United States the suitable areas for this pest are predicted to increase in future climate scenarios by 12% of its territory concentrated in the states of Ohio, Indiana, and Kentucky (Fig 2). In

contrast, Mexico was projected to experience a slight decline of suitable areas for *A. fraterculus* from 39% to 36% of its territory in future scenarios (S2 Table). The Mesoamerican countries such as Panama, Costa Rica, Guatemala, and Honduras display low to high suitability across nearly all their territories in both current and future projections, with minimal temporal variation (Fig 2).

The fruit fly parasitoid, *Doryctobracon areolatus* SDM

Moderate to high suitable areas for *D. areolatus* were identified across South America, particularly along the western and coastal regions of Brazil, as well as in northern Venezuela, Paraguay, Colombia, and Bolivia (Fig 3). In contrast, areas with low suitability were predicted in the Amazon region and some areas in the Andes mountains, spanning Chile, Peru, Ecuador, and Colombia. Additionally, some areas were classified as low to moderate suitable for the establishment of *D. areolatus* in Central and North America (Fig 3).

Fig 3. Suitable areas predicted for *Doryctobracon areolatus* in the Americas. (A) Suitable areas predicted in the current time period; (B-E). Suitable areas predicted under two future climate change scenarios (SSP2-4.5 and SSP5-8.5) (periods 2021-2040 and 2041-2060); (B) scenario SSP2-4.5 time period 2021-2060; (C) scenario SSP2-4.5 time period 2041-2060; (D) scenario SSP5-8.5 time period 2021-2040; (E) scenario SSP5-8.5 time period 2041-2060. The maps show suitability values in a green to-red color where: high suitable areas depicted in green color correspond to suitability values close to 1; moderately suitable areas illustrated in yellow designate suitability values close to 0.5; low suitable areas depicted in red show suitability values close to

the threshold the species (0.31 ~~for *D. areolatus*~~); unsuitable areas represented by the absence of color correspond to values below the threshold and close to 0.

In Brazil, current projections estimate that 69% of the territory is suitable for the establishment of *D. areolatus*, with future climate scenarios predicting an increase of up to 89% (Fig 1). In Peru, suitability is projected to increase from 16% to 36%, and in Colombia, from 26% to 56% (Fig 1), mainly in the Plains and Amazon regions (Fig 3). In Mexico, the current suitable areas predicted for *D. areolatus* are concentrated in the southeast, southwest, and central-south (Fig 3), with future projections suggesting a 10% increase (Fig 1; S2 Table). In the United States, suitable areas were consistently identified along the Southeast Coast, particularly in Florida, under all projections (Fig 3; S2 Table).

Overlapping suitable areas between *A. fraterculus* and *D. areolatus*, were projected in the south and littoral region of Brazil, in the north of Colombia and Venezuela, and in the majority of Mesoamerica (Fig 4). In Mexico, overlapping suitable areas were more predominant in the Yucatan peninsula and in the south part of the country (Fig 4). In contrast, in the United States of America overlapping areas were restricted to the south of Florida (Fig 4). Future projections, show an increase of overlapping suitable areas between *A. fraterculus* and *D. areolatus* in almost all Brazilian territory, except in some areas in the Amazon region and the center region of Brazil (Fig 4). However, In Colombia, common suitable areas between both *A. fraterculus* and *D. areolatus*, were predicted in the Amazon and the Plains (Fig 4). In contrast, in Mexico and the United States of America, common suitable areas for the pest and the parasitoid had no significant change in future predictions (Fig 4).

Fig 4. Overlapping suitable areas predicted for *Doryctobracon areolatus* and *Anastrepha fraterculu*s in the Americas. (A) Overlapped suitable areas predicted in the current time period; (B) Overlapped suitable areas predicted in the future 2041-2060 scenario SSP5-8.5.

The fruit fly parasitoid, *Diachasmimorpha longicaudata* SDM

Our predictions showed that suitable areas for *D. longicaudata* occupy 35.07% of the Americas territory (Table 2). Future projections indicate a slight expansion of suitable areas of up to 38.06% in the Americas (Table 2). Highly to moderate suitable areas for *D. longicaudata* were projected in the Center-West and North of Brazil, with an increase in suitable areas of 14 % in its territory in future predictions (Fig 5, S2 Table). In Peru, suitable areas for the current period were predicted to cover 63% of the national territory, which could increase to 67% in future scenarios (Fig 1; S2 Table). In Colombia, future projections indicate that up to 90% of the country will be suitable for *D. longicaudata* (Fig 1; S2 Table), with moderate to high suitability across most regions, except for the Andean mountains, which remain unsuitable in future predictions (Fig 5.). In the United States, suitable areas are limited to central and southern Florida, with no significant expansion observed in future scenarios (Fig 5). Across Mesoamerica, future projections suggest moderate suitability for *D. longicaudata* along coastal areas and low suitability in the Northeast, lowlands, and central regions of Mexico (Fig 5). Suitable areas in Mexico are expected to increase 17% in future projections.

Fig 5. Suitable areas predicted for *Diachasmimorpha longicaudata* in the Americas (A) Suitable areas predicted in the current time period; (B-E). Suitable areas predicted under two future climate change scenarios (SSP2-4.5 and SSP5-8.5) (periods 2021-2040 and 2041-2060); (B) scenario SSP2-4.5 time period 2021-2060; (C) scenario SSP2-4.5 time period 2041-2060; (D) scenario SSP5-8.5 time period 2021-2040; (E) scenario SSP5-8.5 time period 2041-2060. The maps show suitability values in a green to-red color where: high suitable areas depicted in green color correspond to suitability values close to 1; moderately suitable areas illustrated in yellow designate suitability values close to 0.5; low suitable areas depicted in red show suitability values close to the species threshold (0.15); unsuitable areas represented by the absence of color correspond to values below the threshold and close to 0.

Notably, there is a substantial overlap of suitable areas for *D. longicaudata* and *A. fraterculus*, particularly in South America and Mesoamerica (Fig 6). Current predictions showed suitable overlapping areas across the Amazon region in Brazil, Peru, and Colombia; these areas are predicted to have a significant expansion in future projections (Fig 6). In Mexico, overlapping suitable areas for the establishment of *D. longicaudata* and *A. fraterculus* were predicted along the coast in both current and future predictions (Fig 6). In the United States of America, overlapping suitable areas for the parasitoid and the pest were predicted only in the state of Florida (Fig 6).

Fig 6. Predicted overlapping suitable areas for the establishment of *Diachasmimorph longicaudata* and *Anastrepha fraterculu*s in the Americas. (A) Overlapping suitable areas

predicted in the current time period; (B) Overlapping suitable areas predicted in the future 2041-2060 scenario SSP5-8.5.

Discussion

Anastrepha fraterculus is predicted to expand to new territories in the Americas under future climate change scenarios

Our models predict a reduced ability of *A. fraterculus* to establish in warmer, dry regions, and a high ability to establish in temperate areas (Fig 2). These predictions agree with previous laboratory bioassays that found a reduced emergence of *A. fraterculus* adults at temperatures exceeding 30°C, whereas the emergence rate exceeded 70% at 15 °C [74]. Similarly, Santos [75] concluded that water stress due to scarcity can cause mortality of *A. fraterculus* under warmer conditions. Our results are comparable with those reported for *A. grandis* SDM, where the precipitation of the warmest quarter (BIO10) was the most influential variable, suggesting that extreme drought events may drive the future distribution of this species [76]. Also, in our predictions, regions with temperate climates showed moderate and high suitability for the establishment of *A. fraterculus*, whereas low suitable and unsuitable areas include some tropical climate regions. Based on this, we suggest that temperate climate areas with high humidity seem to be more suitable for the establishment of this species at the current time and in the future. Indeed, the spread of *A. fraterculus* in temperate climate regions due to climate change has already been reported in the field [77].

In Brazil, temperate climates are predominant in the southern and southeastern regions and are characterized by hot summers, no dry seasons, and cooler winters [78]. However, our study found suitable areas for the establishment of *A. fraterculus* in the coastal region characterized by high temperature, low altitude, and high humidity [79]. Our projections concur with previous studies reporting the presence of different *A. fraterculus* morphotypes in these regions. For example, the Brazilian-2 and Brazilian-3 morphotypes seem to be more adapted to coastal areas, whereas the Brazilian-1 morphotype seems to thrive in temperate areas [22,80,81]. Our model predicted areas with low or no suitability for *A. fraterculus* in the Brazilian Amazon region and central-western states. These regions experience tropical climates, characterized by dry winters and summers, whereas the Brazilian Amazon has predominantly tropical monsoon and tropical rainforest climates [78,82]. The low suitability predicted in these areas might be the result of not conducting separate analyses for each *A. fraterculus* morphotype. SDMs developed by Selivon et al. [81] for each *A. fraterculus* morphotype in Brazil, found suitable areas only in the southeast and south of the country. On the other hand, populations of *A. fraterculus* in Mesoamerica, known as the 'Mexican morphotype', were found at low elevations (30 – 1,400m) and thrive in both temperate and tropical climates [22,23]. Therefore, our results of the expansion of the suitable regions for *A. fraterculus* in the Brazilian Amazon are for the species without differentiation between its morphotypes. However, the continued presence of *A. fraterculus* in future scenarios in the Brazilian southern and southeastern regions could pose a risk for crops, such as apples and peaches in these regions [26,83].

The Peruvian mountains, predicted by our study as suitable for *A. fraterculus,* are characterized by two types of climates, temperate and arid [78]. A study conducted by Ramos Peña et al. [84] in the Abancay Valley in Apurimac, Peru, found an increase in fruit fly populations

during the rainy season and the transitions to the dry season. The authors also suggested that low temperatures and the availability of suitable hosts increase fruit fly populations. Studies conducted in guava orchards in Oxapampa, Peru, revealed an increase in the abundance and uniform distribution of *A. fraterculus* at higher elevations. Moreover, these trends appear to be influenced by both seasonality and altitude [85]. In Colombia the presence *A. fraterculus* in coffee crops in the Andean mountains was previously reported by Jaramillo et al. [28], representing 99.9% of all specimens of fruit flies in coffee berries; however, its effects on fruit development and coffee quality are unknown. Additionally, at least 37 other hosts for *A. fraterculus* have been reported in Colombia [86]. Some of these hosts are important crops in the Plains and Amazon regions and will be at risk in the future, such as mango [87,88], soursop (*Annona muricata* L.), papaya (*Carica papaya* L.)[87], and tangerine (*Citrus reticulata* Blanco) [27]. Given the potential increase of suitable areas in the Plains and Amazon regions of Colombia in the coming years, efforts to monitor the dispersion of *A. fraterculus* are highly recommended.

Our results show suitable areas for *A. fraterculus* in the Yucatan peninsula in Mexico, where this pest is currently present [23]. The loss of suitable areas predicted by our models in Mexico can be explained by a reduction of temperate climate regions, transitioning to arid climates in the future [78]. This suggests that arid climates are not optimal for the establishment of the species. Indeed, states such as Chihuahua, Durango, Coahuila, Nuevo Leon, and Zacatecas, characterized by arid climates, were found unsuitable for *A. fraterculus* in all our predictions. The presence of *A. fraterculus* in the United States was previously reported in southern Texas, where the species appears to be restricted [89]. However, our future projections suggest that this pest can spread to new suitable habitats. Therefore, despite the low suitability of the new regions for the

establishment of *A. fraterculus*, it remains crucial to monitor the potential expansion of this pest in the U.S.

Overall, our results predict an expansion in the suitable areas for *A. fraterculus* in temperate regions and some tropical areas across the Americas in the coming years. These predictions support our first hypothesis about the pest's potential distribution. The models suggest this expansion could occur despite some regions becoming less suitable due to climate change. However, additional studies will be needed to determine if the predicted suitable areas for this pest overlap with compatible areas for the cultivation of susceptible crops. Our continental-scale projections provide valuable insights into the pest distribution, but we recognize that local microclimates, agricultural practices, and the presence of different pest morphotypes are essential to determine the establishment of this pest. Nevertheless, monitoring efforts should prioritize regions identified as suitable in our models, especially if valuable host crops are cultivated in those areas.

The fruit fly parasitoid, *Doryctobracon areolatus* as a promising control agent in temperate regions

Suitable areas for *D. areolatus* are predicted in arid and temperate regions, while tropical climates and high-altitude regions seem to be unsuitable or low-suitable for the establishment of this species. Similar results were reported by Núñez-Campero et al. [90], who built SDM for *D. areolatus* in the current period and found that this species is associated with semiarid conditions while high humidity decreases suitability for its establishment. In their projections, precipitation of the driest quarter (BIO17) and precipitation of the coldest quarter (BIO19) were the variables with the greatest contribution to the model with values of 18% and 33%, respectively. Nonetheless, in our models, the bioclimatic variables associated with temperature and precipitation had the

highest contribution values; this discrepancy could be related to different methodologies to construct the models and to different geographic areas of study. However, previous studies demonstrate that the best thermal conditions for the growth of *D. areolatus* are between 22 and 25 °C [91]. Also, studies conducted at different elevations have demonstrated that the parasitism rates of *D. areolatus* are significantly reduced at high altitudes, supporting our results.

In Brazil, certain areas classified by Beck et al. [78] as tropical rainforests in the northern regions of the country were predicted as low suitable or unsuitable for *D. areolatus,* particularly in the Amazonas state (Fig 3). The limited suitability of this parasitoid species in tropical rainforest regions can be attributed to the high precipitation levels in these areas, which affect the population dynamics and reduce the parasitism capacity [5,92]. Our current and future projections indicate that the temperate zones in southern and southeastern Brazil are more suitable for the establishment of *D. areolatus*. These regions are also suitable for the establishment of *A. fraterculus,* suggesting that *D. areolatus* could be a good biological control agent for this pest. For example, in the state of Rio Grande do Sul, the parasitoid was found in association with *A. fraterculus,* exhibiting parasitism rates between 8% and 25% [93,94].

Moreover, overlapping suitable areas for *A. fraterculus* and *D. areolatus* were predicted in other countries across Latin America (Fig 4). For instance, in Peru, Colombia, Venezuela, Ecuador, and Bolivia, the parasitoid *D. areolatus* has been found in association with *A. fraterculus* [20,95]. In Peru, some suitable areas for *D. areolatus* were predicted in the "la Sierra" region, which is heavily planted with fruit crops and is characterized in part by a temperate climate [78,96]. Additionally, a study conducted by Salazar-Mendoza et al. [97] in Oxapampa, Pasco (low elevation in la Sierra region), found 11.76% of *A. fraterculus* parasitized by *D. areolatus* in níspero (*Eriobotrya japonica* Lindl.). However, our predictions for future climate scenarios show a low

suitability of these regions for the establishment of the parasitoid *D. areolatus*. Therefore, we suggest the use of other biocontrol agents to manage *A. fraterculus* in these regions in the future.

Moderately and highly suitable areas in current and future projections were predicted in the Valley of the Andean Colombia, specifically in the departments of Huila, Valle del Cauca, and Tolima (Fig 3). The presence of *D. areolatus* in the department of Valle del Cauca was previously reported by Carrejo and González [98] preying on *Anastrepha obliqua* (Macquart, 1835) in mango crops. Also, *D. areolatus* in Colombia seems to be more common in areas of low elevations [99], which coincides with our predictions of moderate to low suitability in the Atlantic Coast and Plains regions of Colombia. Therefore, the parasitoid *D. areolatus* could help mitigate the effect of *A. fraterculus* and other *Anastrepha* species in crops planted in low-altitude areas.

In Mexico, we found suitable areas for *D. areolatus* in regions characterized by a low elevation and a tropical savannah climate [78]. Previous studies reported that *D. areolatus* was the most predominant parasitoid across various host species of *Anastrepha* in the states of Vera Cruz and Chiapas [100]. Also, Sivinski et al. [101] found that *D. areolatus* was more frequent in low altitudes in the state of Vera Cruz, which supports our findings.

In the United States of America, the parasitoid *D. areolatus* was introduced in 1969 in Florida to control the Caribbean fruit fly, *Anastrepha suspensa* (Loew, 1862) [102]. This parasitoid was successfully established in Florida except in the southern part of the state. Eitam et al. [103] pointed out that the absence of *D. areolatus* in this region can be attributed to competitive displacement due to the introduction of *D. longicaudata*. These authors also found that *D. areolatus* was more common in sites with lower mean temperatures, where greater seasonality and less homogeneous host distribution favor this species.

Collectively, our results suggest that the native parasitoid *D. areolatus* is a promising biological control agent against *Anastrepha* fruit flies, particularly in temperate and arid regions, where its establishment and parasitism rates are highest. Tropical and high-altitude areas seem to be unsuitable or low-suitable due to excessive humidity and cooler temperatures, a trend reinforced by future climate projections. The suitable areas for the establishment of *D. areolatus* are predicted to increase by between 4.72 and 8.13% in future climate change scenarios, contradicting our second hypothesis of no expansion in suitable areas for this species. This suggests that climate change could expand suitable areas also for pests' parasitoids, reinforcing the viability of biological control programs in the future.

The fruit fly parasitoid, *Diachasmimorpha longicaudata* as a promising control agent in tropical regions

According to our projections, highly and moderately suitable areas for *D. longicaudata* have tropical climates, including areas characterized as tropical rainforests like the Amazon region. Low suitability or unsuitability is observed in areas with temperate or arid climate, such as the South of Brazil, Paraguay, along the Andean mountains, and certain states in Northwest and Northeast Mexico (Fig 4). Previous studies demonstrated that the optimum temperature for the development of immature stages and fecundity of *D. longicaudata* is between 25 ºC and 27 ºC, with complete development at 30 ºC in the hosts *Bactrocera dorsalis* (Handel, 1912) and *C. capitata* [104–106]. In another study developed by Meirelles et al. [104], regions with an average temperature higher than 25 ºC were more favorable for establishing *D. longicaudata* as a control agent in the field. Also, according to Harbi et al. [105], temperatures between 16 ºC and 24 ºC and relative humidity between 45% and 60% are ideal for parasitism by *D. longicaudata*. According

to the same authors, a decrease in relative humidity reduces parasitism rates, while an increase in temperature increases parasitism. These findings collectively suggest that tropical regions provide more suitable conditions for the successful establishment and performance of *D. longicaudata*.

In Northern Brazil, areas suitable for *D. longicaudata* overlap with those of the pest *A. fraterculus* (Fig 2 and 4). However, this tropical region has low suitability for the pest, suggesting that *D. longicaudata* could be a good biological control agent of other tephritids, such as *Bactrocera carambolae* (Drew & Hancock, 1994) (Diptera: Tephritidae) in these zones [107]. Moderately suitable areas were also identified in Southeast Brazil, particularly in São Paulo, where *D. longicaudata* could aid in tephritid control in citrus orchards. Paranhos et al. [33] reported that summer releases enhance parasitism, while winter conditions allow high survival rates despite heavy rainfall. In contrast, a low adaptation of *D. longicaudata* was observed before in the state of Amapá in North Brazil [108], which aligns with our current projections for some areas classified as low suitable. However, future predictions indicate that Southeast and North Brazil may become moderately to highly suitable for the establishment of this parasitoid, suggesting that *D. longicaudata* could be used in forthcoming biological control programs of tephritid fruit flies. In the south of Brazil, previous studies developed in Rio Grande do Sul, demonstrated that the parasitoid has limited tolerance to winter, leading to less number of generations [104]. Therefore, other biocontrol agents that are better adapted to the environmental conditions of southern Brazil, such as *D. areolatus,* may be better options for controlling *A. fraterculus*.

In Peru, unlike *D. areolatus*, high and moderately suitable areas were projected across the entire region of “la Selva” and low suitable areas in almost the entire region of “la Sierra” (Fig 4). This suggests that the parasitoid *D. longicaudata* could be an important biocontrol agent for *A. fraterculus* in Peru’s fruit-growing regions. Similarly, in Colombia, *D. longicaudata* may be an

effective biological control agent of *A. fraterculus* particularly in tropical regions that are predicted to be unsuitable or low suitable for the establishment of *D. areolatus*.

In future scenarios, suitable areas for the establishment of *D. longicaudata* were predicted across all Mesoamerica (Fig 5). The coastal regions of Mexico characterized by a tropical climate were predicted as suitable for the establishment of *D. longicaudata*, while the temperate and arid regions of Northeast, and Central Mexico were unsuitable. Our predictions indicate an expansion of suitable areas for *D. longicaudata* in future climate change scenarios, which can be attributed to a projected shift in certain Mexican regions from temperate to tropical [78]. This change could benefit the expansion of *D. longicaudata* populations in the region. Some suitable areas for *D. longicaudata* in Mexico, such as the southern of Chiapas cultivated with mango exhibit parasitism rates between 0.44% and 29.23% on other *Anastrepha* species [109]. Augmentative biological control of *Anastrepha* species in this region has shown that *D. longicaudata* can reduce pest populations without negatively affecting parasitoid local richness [110,111]. Therefore, *D. longicaudata* is a promising biocontrol agent for *A. fraterculus* and other *Anastrepha* species in the tropical climate areas in Mexico.

In the United States, *D. longicaudata* was introduced in 1988 to control *C. capitata* in Florida. This parasitoid reduced the fruit fly populations and is now established in the region [103,112]. The distribution of *D. longicaudata* proposed a decade ago aligns closely with the suitable areas predicted by our model [103]. According to our results, both *D. areolatus* and *D. longicaudata* could be used for the control of *A. fraterculus* in the State of Florida (Fig 4 and 6). However, if the pest spreads to other northern states in the future, where conditions are unsuitable for both parasitoids according to our predictions, the use of alternative control tactics or biocontrol agents may become necessary.

In summary, suitable habitats for *D. longicaudata* may expand in tropical regions under future climate scenarios, contradicting our second and third hypotheses. This suggests that non-native parasitoids like *D. longicaudata* could occupy a broader geographical range than native species under climate change. Importantly, *D. longicaudata* could serve as an effective biocontrol agent against fruit flies, particularly in areas where *D. areolatus* is unsuitable, thereby nearly covering the entire potential range of the pest.

Conclusions

Our study highlights the potential expansion of the South American fruit fly *Anastrepha fraterculus* in the future under climate change, with a potential increase in fruit damage in countries like Brazil and Colombia. Suitable areas for the establishment and dispersion of this pest in the Americas were mostly associated with temperate and some tropical climate regions. Our findings also suggest an increase of suitable areas for the establishment of the parasitoids *D. areolatus* and *D. longicaudata*. The exotic parasitoid *D. longicaudata* could have a greater geographical expansion than *D. areolatus* under future climate change scenarios. Suitable areas for the establishment of *D. longicaudata* were more common in tropical climate regions, especially in tropical rainforests, whereas suitable areas for the establishment of *D. areolatus* were more common in temperate climates. Due to the ability of these parasitoids to thrive in different climates, they could be used as biocontrol agents for *A. fraterculus* in almost all suitable areas for the establishment of the pest. Future studies using SDMs for each *A. fraterculus* morphotype can be informative for designing control tactics at a local level in the Americas. The use of SDMs to predict the establishment of biocontrol agents and pests under climate change is informative and useful for designing pest management strategies.

Acknowledgments

We thank the two anonymous reviewers who helped with the improvement of the manuscript.

References

1. Prentice IC, Farquhar GD, Fasham MJR, Goulden ML, Heimann M, Jaramillo VJ, et al. The carbon cycle and atmospheric carbon dioxide. In: Climate change 2001: the scientific basis, Intergovernmental panel on climate change [Internet]. 2001 [cited 2024 Jan 23]. Available from: https://hal.science/hal-03333974

2. Rosenzweig C. Global climate change: Predictions and observations. Am J Agric Econ. 1989;71(5):1265–1271.

3. Field CB, Barros VR, Mastrandrea MD, Mach KJ, Abdrabo MAK, Adger WN, et al. Summary for policymakers. In: Field CB, Barros VR, Dokken DJ, Mastrandrea MD, Bilir TE, Chatterjee M, et al., editors. Climate change 2014. Cambridge, United Kingdom, and New York, NY, USA: Cambridge University Press; 2015. pp. 1–32.

4. Bale JS, Masters GJ, Hodkinson ID, Awmack C, Bezemer TM, Brown VK, et al. Herbivory in global climate change research: direct effects of rising temperature on insect herbivores. Glob Change Biol. 2002;8(1): 1–16.

5. Hance T, van Baaren J, Vernon P, Boivin G. Impact of extreme temperatures on parasitoids in a climate change perspective. Annu Rev Entomol. 2007;52(1): 107–126.

6. Wu LH, Hoffmann AA, Thomson LJ. Potential impact of climate change on parasitism efficiency of egg parasitoids: A meta-analysis of *Trichogramma* under variable climate conditions. Agric Ecosyst Environ. 2016;231: 143–155.

7. Furlong MJ, Zalucki MP. Climate change and biological control: the consequences of increasing temperatures on host–parasitoid interactions. Curr Opin Insect Sci. 2017; 20:39–44.

8. Chidawanyika F, Mudavanhu P, Nyamukondiwa C. Global climate change as a driver of bottom-up and top-down factors in agricultural landscapes and the fate of host-parasitoid interactions. Front Ecol Evol. 2019;7.

9. Zhang YB, Zhang GF, Liu WX, Wan FH. Variable temperatures across different stages have novel effects on behavioral response and population viability in a host-feeding parasitoid. Sci Rep. 2019;9(1): 2202.

10. Srivastava V, Lafond V, Griess VC. Species distribution models (SDM): Applications, benefits and challenges in invasive species management. CABI Rev. 2019;2019: 1–13.

11. Gillson L, Dawson TP, Jack S, McGeoch MA. Accommodating climate change contingencies in conservation strategy. Trends Ecol Evol. 2013;28(3): 135–142.

12. Kumar S, Neven LG, Yee WL. Evaluating correlative and mechanistic niche models for assessing the risk of pest establishment. Ecosphere. 2014;5(7): art86.

13. Ørsted IV, Ørsted M. Species distribution models of the spotted wing *Drosophila* (*Drosophila suzukii*, Diptera: Drosophilidae) in its native and invasive range reveal an ecological niche shift. J Appl Ecol. 2019;56(2): 423–435.

14. Boys WA, Siepielski AM, Smith BD, Patten MA, Bried JT. Predicting the distributions of regional endemic dragonflies using a combined model approach. Insect Conserv Divers. 2021;14(1): 52–66.

15. Srivastava V, Roe AD, Keena MA, Hamelin RC, Griess VC. Oh the places they'll go: improving species distribution modelling for invasive forest pests in an uncertain world. Biol Invasions. 2021;23(1): 297–349.

16. Wang CJ, Wang R, Yu CM, Dang XP, Sun WG, Li QF, et al. Risk assessment of insect pest expansion in alpine ecosystems under climate change. Pest Manag Sci. 2021;77(7): 3165–3178.

17. Fischbein D, Lantschner MV, Corley JC. Modelling the distribution of forest pest natural enemies across invaded areas: Towards understanding the influence of climate on parasitoid establishment success. Biol Control. 2019;132: 177–188.

18. Lantschner MV, de la Vega G, Corley JC. Predicting the distribution of harmful species and their natural enemies in agricultural, livestock and forestry systems: An overview. Int J Pest Manag. 2019;65(3): 190–206.

19. Zhao H, Yang N, Huang H, Shi J, Xian X, Wan F, et al. Integrating biogeographic approach into classical biological control: Assessing the climate matching and ecological niche

overlap of two natural enemies against common ragweed in China. J Environ Manage. 2023;347: 119095.

20. Garcia FRM, Ovruski SM, Suárez L, Cancino J, Liburd OE. Biological control of Tephritid fruit flies in the Americas and Hawaii: A review of the use of parasitoids and predators. Insects. 2020;11(10): 662.

21. Hernández-Ortiz V, Gómez-Anaya JA, Sánchez A, McPheron BA, Aluja M. Morphometric analysis of Mexican and South American populations of the *Anastrepha fraterculus* complex (Diptera: Tephritidae) and recognition of a distinct Mexican morphotype. Bull Entomol Res. 2004 Dec;94(6): 487–499.

22. Hernández-Ortiz V, Bartolucci AF, Morales-Valles P, Frías D, Selivon D. Cryptic species of the *Anastrepha fraterculus* complex (Diptera: Tephritidae): A multivariate approach for the recognition of South American morphotypes. Ann Entomol Soc Am. 2012;105(2): 305–318.

23. Hernández-Ortiz V, Canal NA, Salas JOT, Ruíz-Hurtado FM, Dzul-Cauich JF. Taxonomy and phenotypic relationships of the *Anastrepha fraterculus* complex in the Mesoamerican and Pacific Neotropical dominions (Diptera, Tephritidae). ZooKeys. 2015 Nov 26;(540): 95–124.

24. Nava DE, Botton M. Bioecologia e controle de *Anastrepha fraterculus* e *Ceratitis capitata* em pessegueiro. Pelotas. Embrapa Clima Temperado. 2010 [cited 2024 Jan 26]; Available from: http://www.infoteca.cnptia.embrapa.br/infoteca/handle/doc/888672

25. Zucchi RA, Moraes RCB de, Malavasi A, Adaime R, Nava DE. Moscas-das-frutas, suas plantas hospedeiras e parasitoides no Brasil: dados consolidados (2022). Moscas-das-frutas no Brasil conhecimento básico e aplicado [Internet]. 2023 [cited 2024 Jan 26];2. Available from: https://repositorio.usp.br/item/003123905

26. Dias VS, Lucky A. South American fruit fly (suggested common name) *Anastrepha fraterculu*s (Wiedemann)(Insecta: Diptera: Tephritidae): EENY-696/IN1196, 12/2017. EDIS [Internet]. 2018 [cited 2024 Jan 26];2018(1). Available from: https://journals.flvc.org/edis/article/download/105365/126710

27. Castañeda M del R, Osorio F A, Canal NA, Galeano PÉ. Especies, distribución y hospederos del género *Anastrepha* Schiner en el departamento del Tolima, Colombia. Agron Colomb. 2010;28(2): 264–272.

28. Jaramillo MG, Machado PB, Sáenz JRR, Montoya D, Valencia FF, Pinzón JFT. Las moscas de las frutas de la zona central cafetera de Colombia y su clave ilustrada. Cenicafé. 2015;66(2):58–72.

29. ICA[Internet]. Plagas cuarentenarias presentes [cited 2024 Jun 6]. Available from: https://www.ica.gov.co/areas/agricola/servicios/epidemiologia-agricola/plagas-reglamentadas/plagas-cuarentenarias-presentes.aspx

30. Hernández Calderón FA. Etapas de la erradicación y manejo integrado de la mosca de la fruta (*Ceratitis capitata* Wied) en la Región Ica. Thesis, Universidad Nacional Agraria la Molina. 2016. Available from: http://repositorio.lamolina.edu.pe/handle/20.500.12996/1783

31. Perez-Staples D, Diaz-Fleischer F, Montoya P, Vera MT. Area-wide management of fruit fly pests. CRC Press; 2019.

32. Montoya P, Cancino J, Zenil M, Santiago G, Gutierrez JM. The augmentative biological control component in the Mexican national campaign against *Anastrepha* spp. fruit flies. In: Vreysen MJB, Robinson AS, Hendrichs J, editors. Area-wide control of insect pests. Dordrecht: Springer Netherlands; 2007. pp. 661–670.

33. Paranhos BJ, Nava DE, Malavasi A. Biological control of fruit flies in Brazil. Pesqui Agropecuária Bras. 2019;54: e26037.

34. Dias NP, Montoya P, Nava DE. A 30-year systematic review reveals success in tephritid fruit fly biological control research. Entomol Exp Appl. 2022;170(5): 370–384.

35. Alvarado-Gálvez L, Medianero E. Especies de parasitoides asociados a moscas de la fruta del género *Anastrepha* (Diptera: Tephritidae) en Panamá, República de Panamá. Sci Panamá. 2015;25: 47–62.

36. Monteiro LB, Nishimura G, Monteiro RS. Natural parasitism in fruit fly (Diptera: Tephritidae) and interaction with wild hosts surrounding apple orchards adjacent to Atlantic Forest fragments in Paraná State, Brazil. Braz J Biol. 2021;83: e250505.

37. Nunes AM, Nava DE, Müller FA, Gonçalves R da S, Garcia MS. Biology and parasitic potential of *Doryctobracon areolatus* on *Anastrepha fraterculus* larvae. Pesqui Agropecuária Bras. 2011;46: 669–671.

38. Nava DE, Gonçalves R da S, Nornberg S, Scheunemann T, Grutzmacher A. Avaliação preliminar da seletividade de inseticidas e do parasitismo de *Doryctobracon areolatus* (Hymenoptera: Braconidae) em moscas-das-frutas. Empresa Brasileira de Pesquisa Agropecuária: Embrapa Clima Temperado, Ministério da Agricultura, Pecuária e Abastecimento. 2019, Boletin de Pesquisa e Desenvolvimiento, ISSN 1678-2518; 325.

39. Ovruski SM, Bezdjian LP, Nieuwenhove GAV, Albornoz-Medina P, Schliserman P. Host preference by *Diachasmimorpha longicaudata* (Hymneoptera: Braconidae) reared on larvae of *Anastrepha fraterculus* and *Ceratitis capitata* (Diptera: Tephritidae). Fla Entomol. 2011 Jun;94(2): 195–200.

40. Ovruski SM, Oroño LE, Schliserman P, Nuñez-Campero S. The effect of four fruit species on the parasitization rate of *Anastrepha fraterculus* (Diptera: Tephritidae, Trypetinae) by *Diachasmimorpha longicaudata* (Hymenoptera: Braconidae, Opiinae) under laboratory rearing conditions. Biocontrol Sci Technol. 2007;17(10): 1079–1085.

41. Governo federal do Brasil, Portaria DAS n 784, de 19 de abril 2023 (April 19, 2023).

42. Ovruski S, Aluja M, Sivinski J, Wharton R. Hymenopteran parasitoids on fruit-infesting Tephritidae (Diptera) in Latin America and the Southern United States: Diversity, distribution, taxonomic status and their use in fruit fly biological control. Integr Pest Manag Rev. 2000;5(2): 81–107.

43. Carvalho R da S. Avaliação das liberações inoculativas do parasitóide exótico *Diachasmimorpha longicaudata* (Ashmead)(Hymenoptera: Braconidae) em pomar diversificado em Conceição do Almeida, BA. Neotrop Entomol. 2005 Oct;34: 799–805.

44. Cancino J, Ruiz L, López E, Aguilar E, Galvez C, Montoya P, et al. Suppression of *Ceratitis capitata* (Wied.) (Diptera: Tephritidae) populations in coffee in the Mexico–Guatemala border region through the augmentative releases of *Diachasmimorpha longicaudata* (Ashmead) (Hymenoptera: Braconidae). Biocontrol Sci Technol. 2019 Aug 3;29(8): 822–826.

45. Ovruski SM, Colin C, Soria A, Oroño LE, Schliserman P. Introducción y producción en laboratorio de *Diachasmimorpha tryoni* y *Diachasmimorpha longicaudata* (Hymenoptera: Braconidae) para el control biológico de *Ceratitis capitata* (Diptera: Tephritidae) en la Argentina. Rev Soc Entomológica Argent. 2003;62(3–4): 49–59.

46. Governo federal do Brasil, Instrução normativa conjunta SDA/SMC n 2, de 29 de agosto de 2018. (Aug 29, 2018).

47. Walder JMM. Produção de moscas-das-frutas e seus inimigos naturais: associação de moscas estéreis e controle biológico. Controle Biológico no Brasil Parasitóides e Predadores. São Paulo, Manole. Brazil; 2002. p. 181–190.

48. Sugayama RL, Malavasi A. Anastrepha fraterculus (Wiedemann) (Diptera: Tephritidae) na região produtora de maçãs do Rio Grande do Sul: relação com seus inimigos naturais e potencial para controle biológico. Ph. D. Thesis, Universidade de São Paulo. 2000. Available from: https://repositorio.usp.br/item/001083555

49. Owens H, Barve V, Chamberlain S, Ram K, Hart T, rOpenSci (ropensci.org/). spocc: Interface to species occurrence data sources. 2023 [cited 2024 Feb 2]. Available from: https://cran.r-project.org/web/packages/spocc/index.html

50. Moreira G da C, Bosco NS, Shimbori EM, Sobral-Souza T, Onody HC. Synthesis of the knowledge of braconids in Brazil: list of species, data gaps and spatial biases in the sampling. [Preprint]. 2023 [cited 2024 Jun 21]. Available from: https://www.researchsquare.com/article/rs-2527415/v1

51. Hijmans RJ, Phillips S, Elith JL and J. dismo: Species distribution modeling. 2023 [cited 2024 Sep 8]. Available from: https://cran.r-project.org/web/packages/dismo/index.html

52. R Core Team. R: A language and environment for statistical computing. Vienna, Austria: R Foundation for Statistical Computing; 2023. Available from: https://www.R-project.org/

53. Barbet-Massin M, Jiguet F, Albert CH, Thuiller W. Selecting pseudo-absences for species distribution models: how, where and how many?. Methods Ecol Evol. 2012;3(2): 327–338.

54. Fick SE, Hijmans RJ. WorldClim 2: new 1-km spatial resolution climate surfaces for global land areas. Int J Climatol. 2017;37(12): 4302–4315.

55. O'Neill BC, Tebaldi C, Van Vuuren DP, Eyring V, Friedlingstein P, Hurtt G, et al. The scenario model intercomparison project (ScenarioMIP) for CMIP6. Geosci Model Dev. 2016;9(9): 3461–3482.

56. Thomson AM, Calvin KV, Smith SJ, Kyle GP, Volke A, Patel P, et al. RCP4.5: a pathway for stabilization of radiative forcing by 2100. Clim Change. 2011;109(1): 77.

57. Riahi K, Rao S, Krey V, Cho C, Chirkov V, Fischer G, et al. RCP 8.5—A scenario of comparatively high greenhouse gas emissions. Clim Change. 2011;109(1): 33.

58. Graham MH. Confronting multicollinearity in ecological multiple regression. Ecology. 2003;84(11): 2809–2815.

59. Dormann CF, Elith J, Bacher S, Buchmann C, Carl G, Carré G, et al. Collinearity: a review of methods to deal with it and a simulation study evaluating their performance. Ecography. 2013;36(1): 27–46.

60. Naimi B, Hamm NAS, Groen TA, Skidmore AK, Toxopeus AG. Where is positional uncertainty a problem for species distribution modelling?. Ecography. 2014;37(2): 191–203.

61. Mi C, Huettmann F, Guo Y, Han X, Wen L. Why choose Random Forest to predict rare species distribution with few samples in large undersampled areas? Three Asian crane species models provide supporting evidence. PeerJ. 2017 ;5: e2849.

62. Luan J, Zhang C, Xu B, Xue Y, Ren Y. The predictive performances of random forest models with limited sample size and different species traits. Fish Res. 2020;227: 105534.

63. Li Z, Ye Z, Wan R, Zhang C. Model selection between traditional and popular methods for standardizing catch rates of target species: A case study of Japanese Spanish mackerel in the gillnet fishery. Fish Res. 2015;161: 312–319.

64. Breiman L. Random forests. Mach Learn. 2001;45(1): 5–32.

65. Hastie T, Tibshirani R, Friedman J. Random Forests. In: Hastie T, Tibshirani R, Friedman J, editors. The elements of statistical learning: Data mining, inference, and prediction. New York, NY: Springer; 2009. pp. 587–604.

66. Probst P, Wright MN, Boulesteix A. Hyperparameters and tuning strategies for random forest. WIREs Data Min Knowl Discov. 2019;9(3): e1301.

67. Probst P, Boulesteix AL, Bischl B. Tunability: Importance of hyperparameters of machine learning algorithms. J Mach Learn Res. 2019;20(53): 1–32.

68. Kuhn M. A Short Introduction to the caret Package. R Found Stat Comput. 2015;1: 1–10.

69. Robin X, Turck N, Hainard A, Tiberti N, Lisacek F, Sanchez JC, et al. pROC: an open-source package for R and S+ to analyze and compare ROC curves. BMC Bioinformatics. 2011;12(1): 77.

70. Hijmans RJ, Bivand R, Pebesma E, Sumner MD. terra: Spatial data analysis. 2024 [cited 2024 Mar 24]. Available from: https://cran.r-project.org/web/packages/terra/index.html

71. Massicotte P, South A, Hufkens K. rnaturalearth: World map data from natural earth. 2023 [cited 2025 Jan 7]. Available from: https://cran.rproject.org/web/packages/rnaturalearth/index.html

72. Fielding AH, Bell JF. A review of methods for the assessment of prediction errors in conservation presence/absence models. Environ Conserv. 1997;24(1): 38–49.

73. Araújo MB, Pearson RG, Thuiller W, Erhard M. Validation of species–climate impact models under climate change. Glob Change Biol. 2005;11(9): 1504–1513.

74. Galvão-Silva FL, Araújo AS, Dias VS, do Nascimento AS, Joachim-Bravo IS. Responses of two *Anastrepha* species' immature stages infesting preferential hosts to different temperature exposures. Neotrop Entomol. 2024; 53: 342-350.

75. Santos W da S. Zoneamento ecológico de *Anastrepha fraterculus* e *Ceratitis capitata* (Diptera: Tephritidae) em dois cenários climáticos no Brasil. Ph. D. Thesis. Universidade de São Paulo. 2008. Available from: http://www.teses.usp.br/teses/disponiveis/11/11146/tde-12062008-161926/

76. Machado Teixeira C, Peter Krüger A, Nava DE, Mello Garcia FR. Global potential distribution of *Anastrepha grandis* (Diptera, Tephritidae) under climate change scenarios. Crop Prot. 2022;151: 105836.

77. Skendžić S, Zovko M, Živković IP, Lešić V, Lemić D. The impact of climate change on agricultural insect pests. Insects. 2021;12(5): 440.

78. Beck HE, McVicar TR, Vergopolan N, Berg A, Lutsko NJ, Dufour A, et al. High-resolution (1 km) Köppen-Geiger maps for 1901–2099 based on constrained CMIP6 projections. Sci Data. 2023;10(1): 724.

79. Morellato LPC, Haddad CFB. Introduction: The Brazilian Atlantic forest1. Biotropica. 2000;32(4): 786.

80. Selivon D, Perondini ALP, Morgante JS. A genetic–morphological characterization of Two cryptic species of the *Anastrepha fraterculus* complex (Diptera: Tephritidae). Ann Entomol Soc Am. 2005;98(3): 367–381.

81. Selivon D, Perondini ALP, Hernández-Ortiz V, doVal FC, Camacho A, Gomes FR, et al. Genetical, morphological, behavioral, and ecological traits support the existence of three Brazilian species of the *Anastrepha fraterculus* complex of cryptic species. Front Ecol Evol. 2022;10.

82. Rubel F, Kottek M. Observed and projected climate shifts 1901-2100 depicted by world maps of the Köppen-Geiger climate classification. Meteorol Z. 2010;19(2): 135.

83. Araujo ES, Monteiro LB, Monteiro RS, Nishimura G, Franck P, Lavigne C. Impact of native forest remnants and wild host plants on the abundance of the South American fruit fly, *Anastrepha fraterculus* in Brazilian apple orchards. Agric Ecosyst Environ. 2019;275: 93–99.

84. Ramos Peña ÁM, Yábar Landa E, Ramos Peña JC, Ramos Peña ÁM, Yábar Landa E, Ramos Peña JC. Diversidad, fluctuación poblacional y hospedantes de moscas de la fruta *Anastrepha* spp. y *Ceratitis capitata* (Wiedemann) (Diptera: Tephritidae) en el Valle de Abancay, Apurímac, Perú. Acta Zool Mex. 2019;35.

85. Salazar-Mendoza P, Peralta-Aragón I, Romero-Rivas L, Salamanca J, Rodriguez-Saona C. The abundance and diversity of fruit flies and their parasitoids change with elevation in guava orchards in a tropical Andean Forest of Peru, independent of seasonality. PLOS ONE. 2021;16(4): e0250731.

86. Hernández-Ortiz V, Barradas-Juanz N, Díaz-Castelazo C. A Review of the natural host plants of the *Anastrepha fraterculus* complex in the Americas. In: Area-wide management of fruit fly pests. CRC Press; 2019.

87. Mendoza RG. Contribución al estudio de las moscas Anastrephas en Colombia. Rev Fac Nac Agron Medellín. 1952;12(42): 423–549.

88. Nùñez Bueno L. Contribución al reconocimiento de las moscas de las frutas (Diptera: Tephriridae) en Colombia. Rev ICA. 1981;(4): 173–179.

89. Rust EW. Anastrepha fraterculus Wied.(Trypetidae)—A severe menace to the southern United States. J Econ Entomol. 1918;11(6): 457–467.

90. Núñez-Campero SR, González C, Rull J, Ovruski SM. Maximum Entropy (MaxEnt) as extreme distribution indicator of two Neotropical fruit fly parasitoids in irrigated drylands of Argentina. Bull Entomol Res. 2022;112(5): 636–645.

91. da Silva Gonçalves R, Nunes AM, Poncio S, Manica-Berto R, Nörnberg SD, Grützmacher AD, et al. Bionomics, thermal requirements, and life table of the fruit fly parasitoid *Doryctobracon areolatus* (Hymenoptera: Braconidae) under various thermal regimes. Biol Control. 2018;127: 101–108.

92. Coley PD. Possible effects of climate change on plant/herbivore interactions in moist tropical forests. Clim Change. 1998;39(2): 455–472.

93. Jahnke SM, Ponte EMD, Redaelli LR, Rego DRGP. Spatial patterns and associations of *Anastrepha fraterculus* (Diptera: Tephritidae) and its parasitoid *Doryctobracon areolatus* (Hymenoptera: Braconidae) in organic orchards of *Psidium guajava* and *Acca sellowiana*. Fla Entomol. 2014;97(2): 744–752.

94. Piovesan B, Gonçalves R da S, Nörnberg SD, Llano JHG, Contreras-Miranda J, Bernardi D, et al. Spatial and temporal dispersion of *Doryctobracon areolatus* (Szépligeti) (Hymenoptera: Braconidae) in orchards infested with *Anastrepha fraterculus* (Wiedemann) (Diptera: Tephritidae). Environ Entomol. 2023;52(4): 565–573.

95. Ovruski SM, Schliserman P, Oroño LE, Nuñéz-Campero SR, Albornoz-Medina P, Bezdjian LP, et al. Natural occurrence of Hymenopterous parasitoids associated with *Anastrepha*

fraterculus (Diptera: Tephritidae) in Myrtaceae species in Entre Rios, Northeastern Argentina. Fla Entomol. 2008;91(2): 220–227.

96. Banco Mundial. Tomando impulso en la agricultura Peruana: oportunidades para aumentar la productividad y mejorar la competitividad del sector. 2017 [cited 2024 Jun 18]. Available from: https://documents.worldbank.org/en/publication/documents-reports/documentdetail/781561519138355286/Gaining-momentum-in-Peruvian-agriculture-opportunities-to-increase-productivity-and-enhance-competitiveness

97. Salazar-Mendoza P, Peralta-Aragón I, Romero Rivas L. Análisis faunístico, fluctuación poblacional y parasitoides asociados a las moscas de la fruta (Diptera: Tephritidae) en el níspero. Rev Perú Entomol. 2018 Dec 1;53: 21–32.

98. Carrejo NS, O. RG. Parasitoids reared from species of *Anastrepha* (Diptera: Tephritidae) in Valle del Cauca, Colombia. Fla Entomol. 1999;82(1): 113–118.

99. Saavedra-Díaz J, Galeano-Olaya PE, Canal D NA. Relaciones ecológicas entre frutos hospederos, moscas frugívoras y parasitoides en un fragmento de bosque seco tropical. Rev Cienc Agríc. 2017;34(1): 32–49.

100. Montoya P, Ayala A, López P, Cancino J, Cabrera H, Cruz J, et al. Natural parasitism in fruit fly (Diptera: Tephritidae) populations in disturbed areas adjacent to commercial mango orchards in Chiapas and Veracruz, Mexico. Environ Entomol. 2016;45(2): 328–337.

101. Sivinski J, Piñero J, Aluja M. The distributions of parasitoids (Hymenoptera) of *Anastrepha* fruit flies (Diptera: Tephritidae) along an altitudinal gradient in Veracruz, Mexico. Biol Control. 2000;18(3): 258–269.

102. Baranowski RM, Swanson RW. Introduction of *Parachasma* (=*Opius*) *Cereus* (Hymenoptera: Braconidae) into Florida as a parasite of *Anastrepha suspensa* (Diptera: Tephritidae). Fla Entomol. 1970;53(3): 161–162.

103. Eitam A, Sivinski J, Holler T, Aluja M. Biogeography of braconid parasitoids of the Caribbean fruit fly (Diptera: Tephritidae) in Florida. Ann Entomol Soc Am. 2004;97(5): 928–939.

104. Meirelles RN, Redaelli LR, Ourique CB. Thermal requirements and annual number of generations of *Diachasmimorpha longicaudata* (Hymenoptera: Braconidae) reared in the South American fruit fly and the Mediterranean fruit fly (Diptera: Tephritidae). Fla Entomol. 2015;98(4): 1223–1226.

105. Harbi A, Beitia F, Chermiti B, de Pedro L, Ferrara F, Asís JD, et al. Abiotic factors affecting *Diachasmimorpha longicaudata* (Hymenoptera: Braconidae) activity as a natural enemy of *Ceratitis capitata* (Diptera: Tephritidae) under semi-natural conditions in the Mediterranean region. J Appl Entomol. 2018;142(8): 755–764.

106. Ndlela S, Azrag AGA, Mohamed SA. Determination of temperature thresholds for the parasitoid *Diachasmimorpha longicaudata* (Hymenoptera: Braconidae), using life cycle simulation modeling: Implications for effective field releases in classical biological control of fruit flies. PLOS ONE. 2021;16(8): e0255582.

107. Soares GKA, Fidelis EG, Farias ES, Rodrigues GS, Paes JLA. Range expansion and population dynamics of *Bactrocera carambolae* in Roraima, Brazil. Crop Prot. 2023;165: 106167.

108. Carvalho RS. Estudos de laboratório e de campo com o parasitóide exótico *Diachasmimorpha longicaudata* Ashmead (Hymenoptera. Braconidae. no Brasil. Ph.D. Thesis, Universidade de São Paulo. 2003.

109. Aluja M, Guillen J, Liedo P, Cabrera M, Rios E, de la Rosa G, et al. Fruit infesting tephritids [Dipt.: Tephritidae] and associated parasitoids in Chiapas, Mexico. Entomophaga. 1990;35(1): 39–48.

110. Montoya P, Liedo P, Benrey B, Cancino J, Barrera JF, Sivinski J, et al. Biological control of *Anastrepha* spp. (Diptera: Tephritidae) in mango orchards through augmentative releases of *Diachasmimorpha longicaudata* (Ashmead) (Hymenoptera: Braconidae). Biol Control. 2000;18(3): 216–224.

111. Montoya P, López P, Cruz J, López F, Cadena C, Cancino J, et al. Effect of *Diachasmimorpha longicaudata* releases on the native parasitoid guild attacking *Anastreph*a spp. larvae in disturbed zones of Chiapas, Mexico. BioControl.;62(5):581–593.

112. Baranowski R, Glenn H, Sivinski J. Biological control of the Caribbean fruit fly (Diptera: Tephritidae). Fla Entomol. 1993;76(2): 245–251.

Supporting information

S1 Fig. Overlapping suitable predicted areas for *A. fraterculus* and *D. areolatus* in the future. Overlapping suitable areas were predicted for *A. fraterculus* and *D. areolatus* under two future climatic scenarios (SSP2-4.5 and SSP5-8.5) and two time periods (2021-2040 and 2041-2060).

S2 Fig. Overlapping suitable predicted areas for *A. fraterculus* and *D. longicaudata* in the future. Overlapping suitable areas were predicted for *A. fraterculus* and *D. longicaudata* under two future climatic scenarios (SSP2-4.5 and SSP5-8.5) and two time periods (2021-2040 and 2041-2060).

S1 Table. Global Climate Models (GCM) from the Coupled Model Intercomparison Project Phase 6 (CMIP6) used in our RF model construction.

S2 Table. Extension of suitable and unsuitable areas predicted for the pest, *A. fraterculus* and the parasitoids *D. areolatus* and *D. longicaudata* across the Americas.

Fig_1

Click here to access/download;Figure;Fig_1.tif

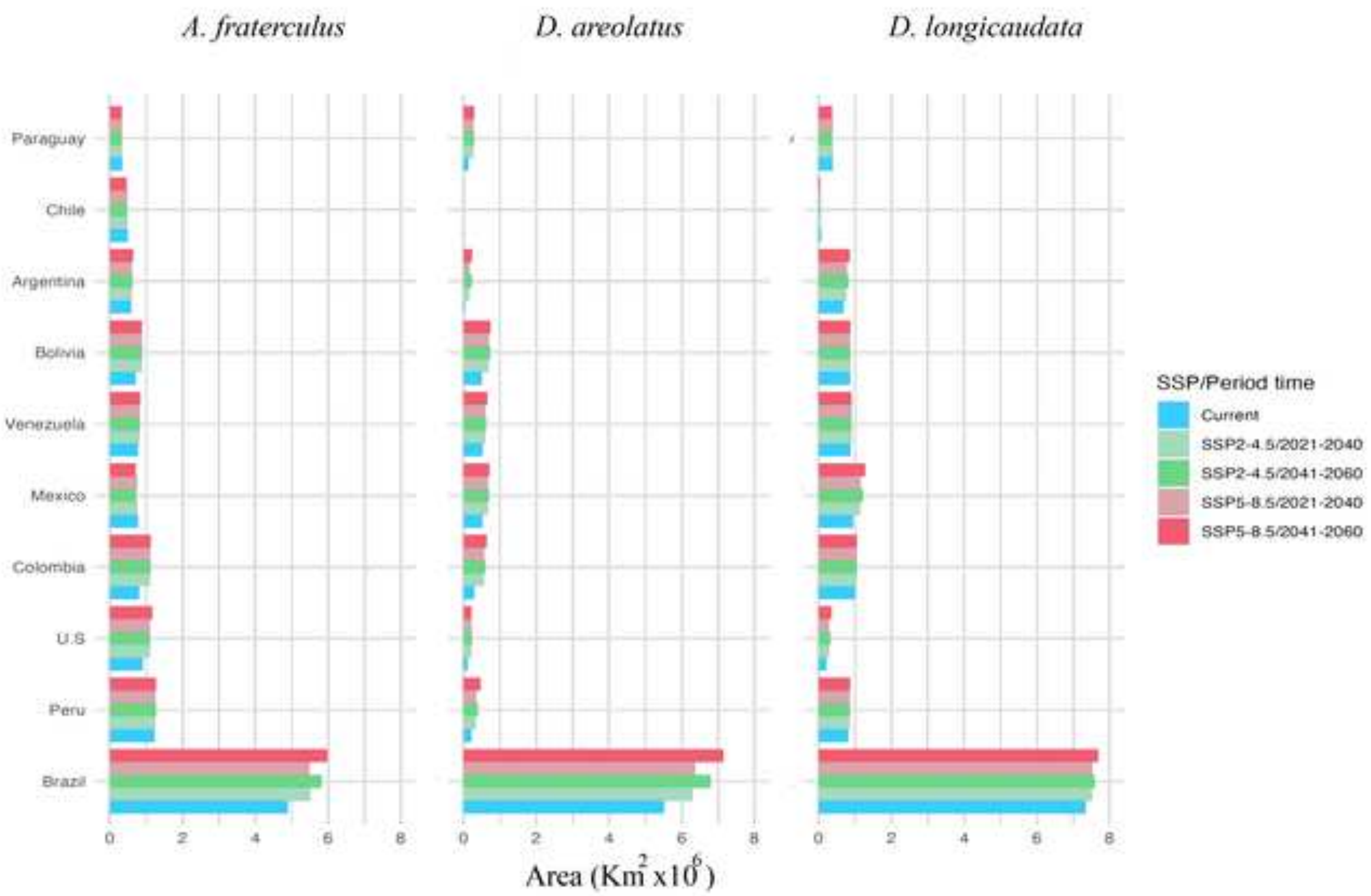

Click here to access/download;Figure;Fig_2.tif

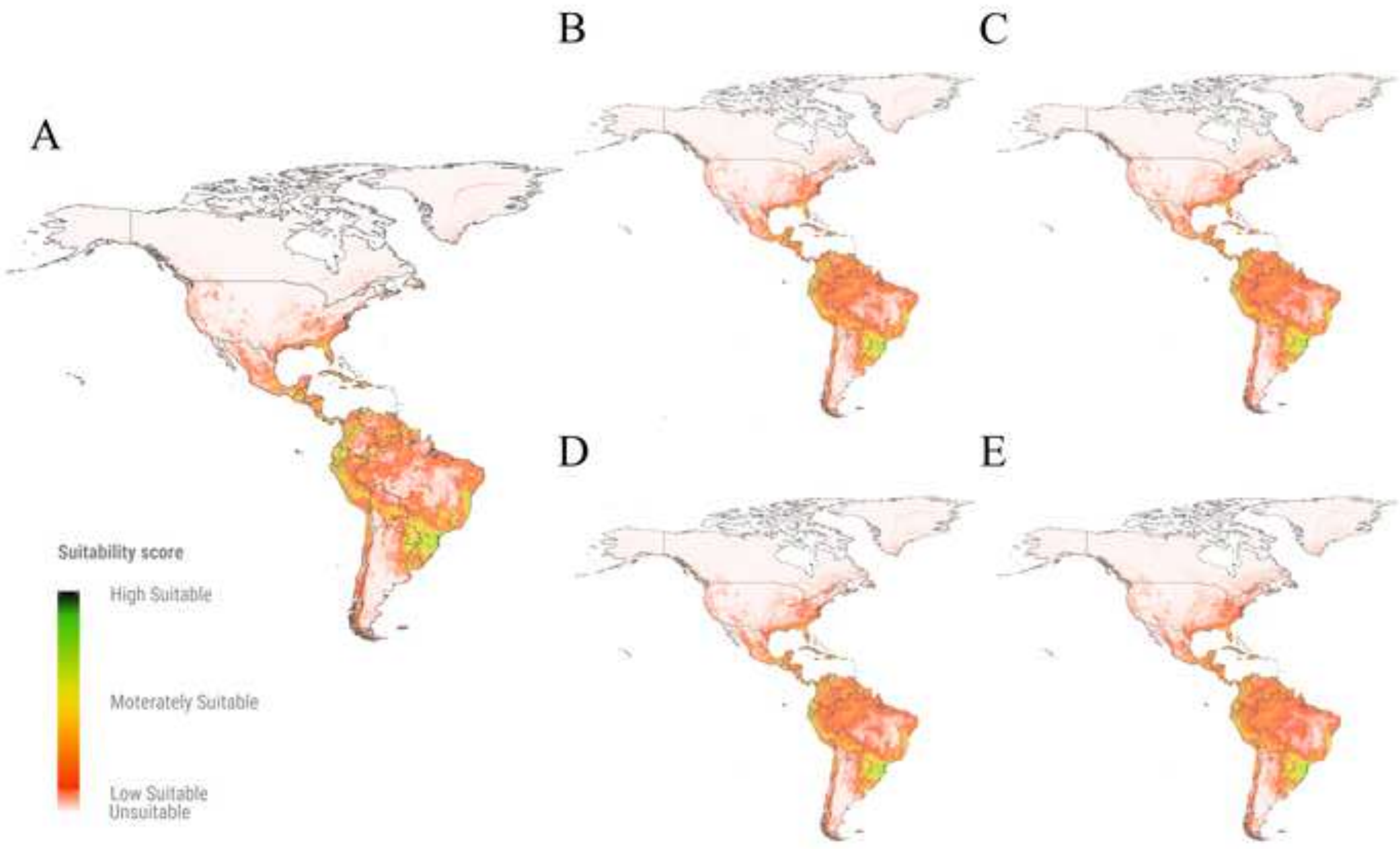

Fig_3

Click here to access/download;Figure;Fig_3.tif

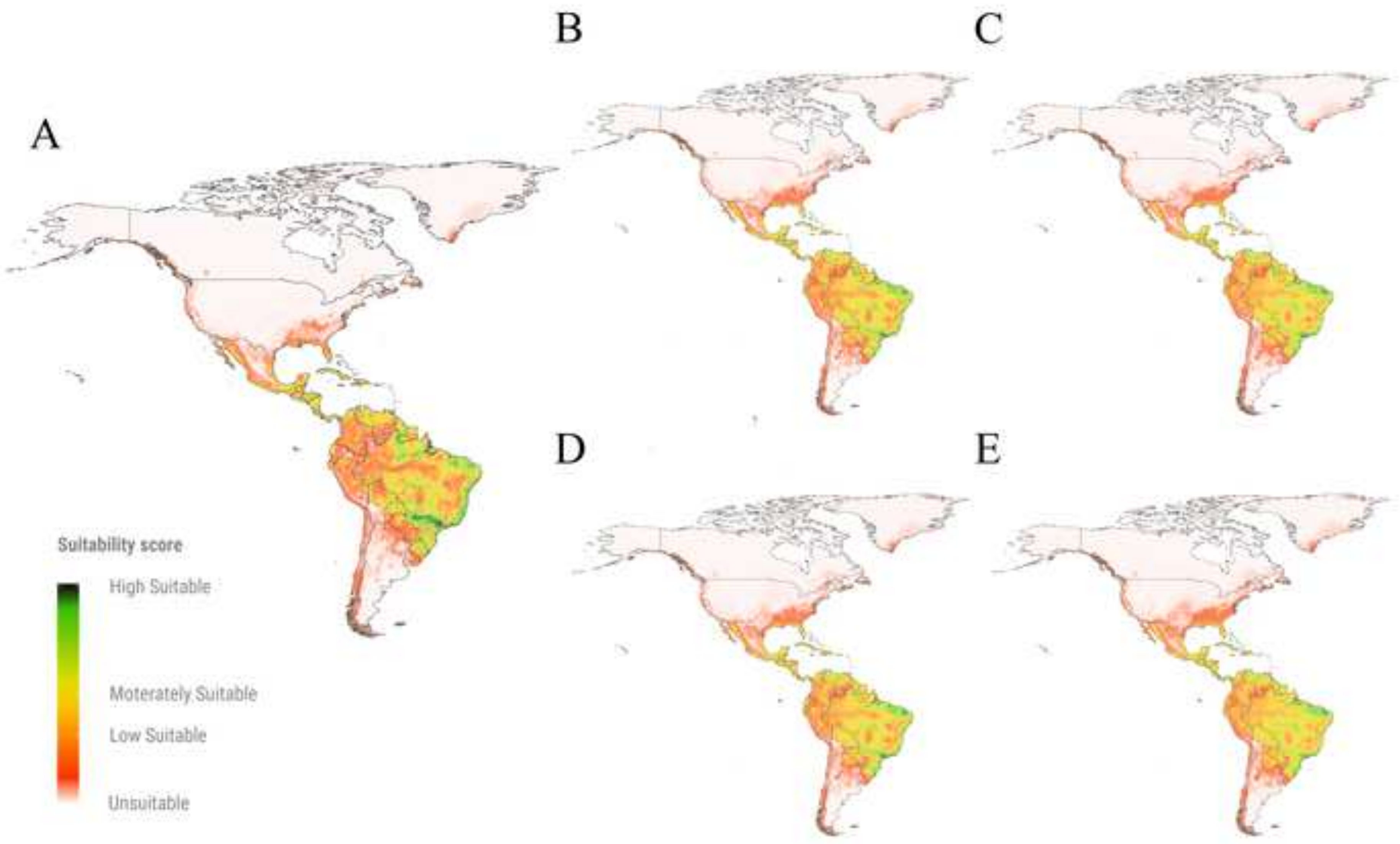

Fig_4 Click here to access/download;Figure;Fig_4.tif

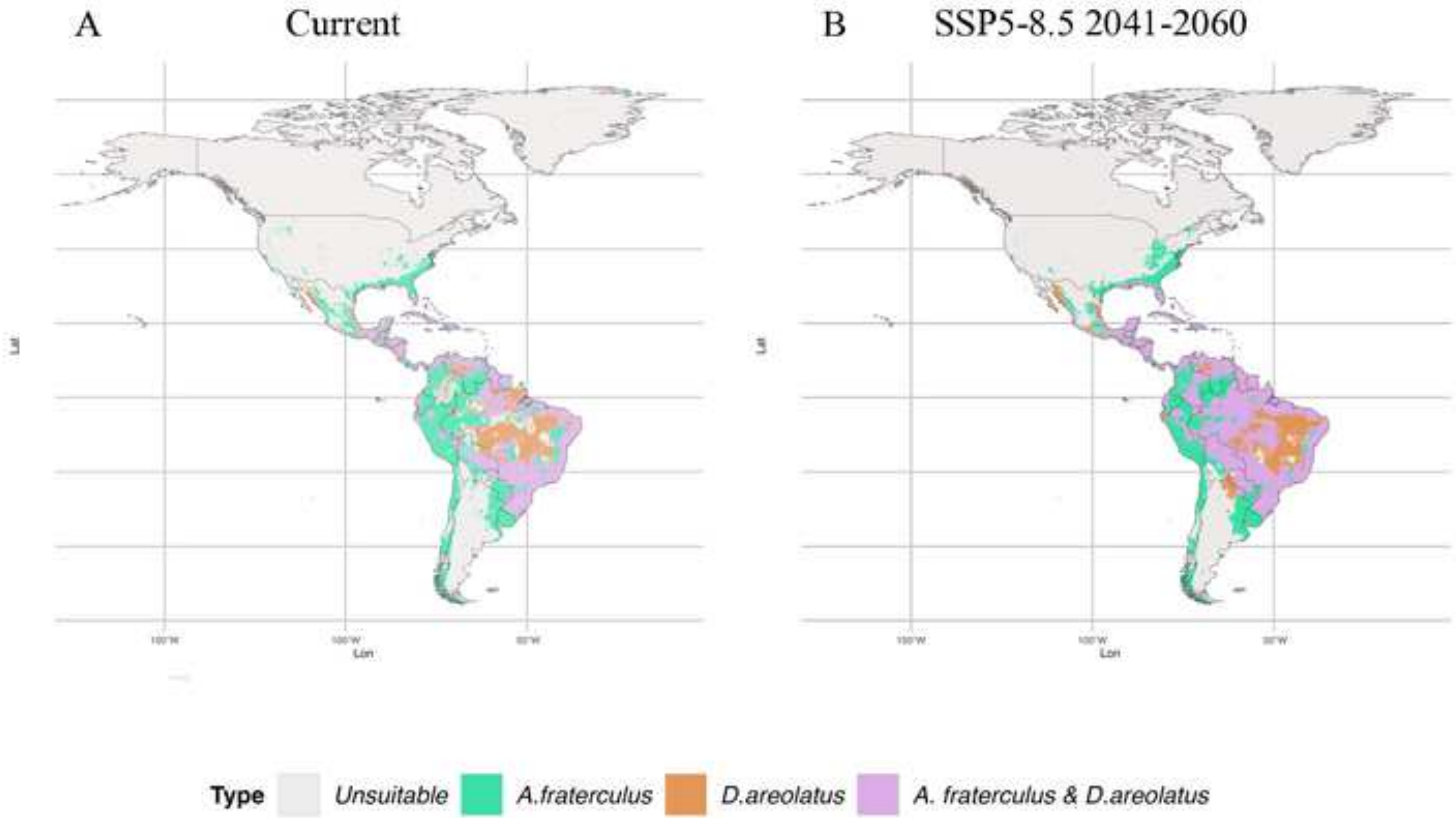

Click here to access/download;Figure;Fig_5.tif

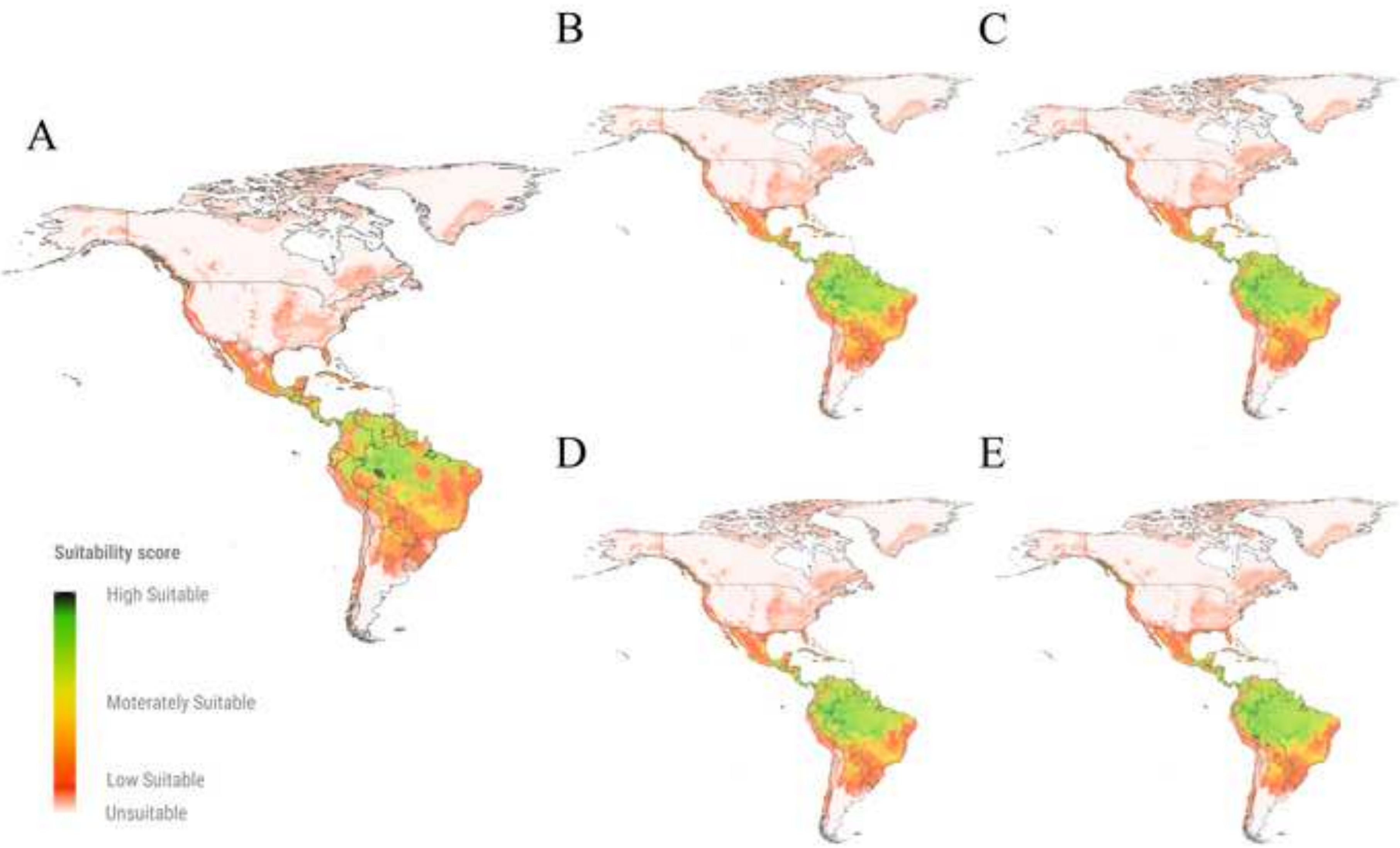

Fig_6

Click here to access/download;Figure;Fig_6.tif

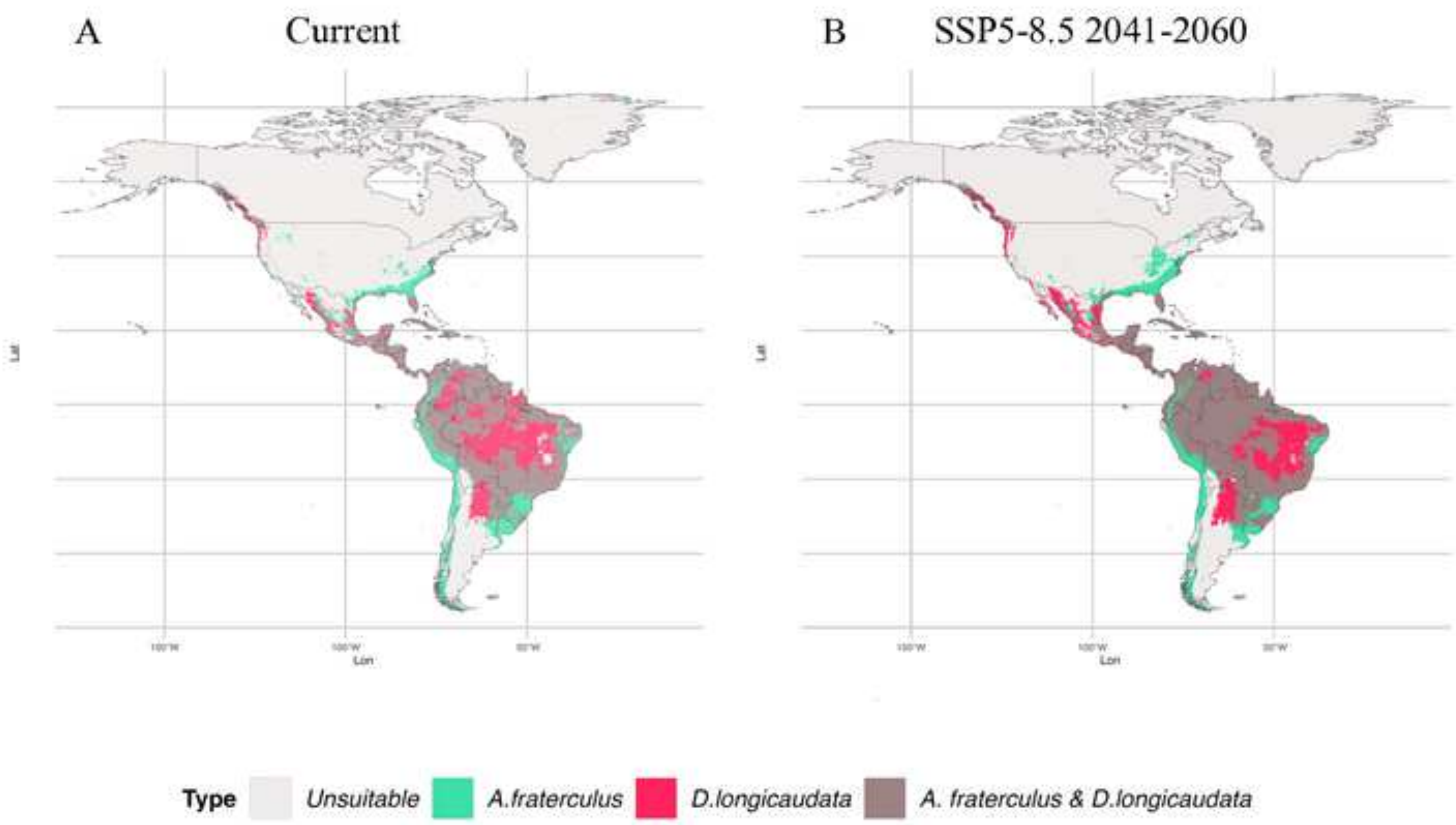

S1_Fig

Click here to access/download
Supporting Information
S1_Fig.tif

S2_Fig

Click here to access/download
Supporting Information
S2_Fig.tif

Formatted: Font: 18 pt

Biological control under climate change: Distribution patterns of the South American fruit fly, *Anastrepha fraterculus* and two of its parasitoids in the Americas

Jesús Hernando Gómez Llano[1]*, Fábio L. Galvão-Silva[1], Flor Edith Acevedo[2*], Fabio Castro Llanos[3], Marco Silva Gottschalk[4], Dori Edson Nava[5]

[1]Universidade Federal de Pelotas (UFPEL), Av. Eliseu Maciel – Campus Universitário Capão do Leão. Capão do Leão, Rio Grande do Sul, Brazil.

[2]Department of Entomology, The Pennsylvania State University, University Park, PA, United States

[3]International Center for Tropical Agriculture (CIAT), Valle del Cauca, Colombia.

[4]Departamento de Ecologia, Zoologia e Genética, Universidade Federal de Pelotas (UFPel), Capão do Leão, Rio Grande do Sul, Brazil.

[5]Embrapa Clima Temperado, Pelotas, Rio Grande do Sul, Brazil.

* Corresponding authors:

E-mail: jesushernandogll@gmail.com , fea5007@psu.edu

Abstract

Climate change affects the distribution of insects, such as pests and parasitoids. Species Distribution Models (SDMs) have been developed to determine distribution patterns and risk areas for pests and biological control agents under different climate change scenarios. The South American Fruit Fly, *Anastrepha fraterculus* (Wiedemann) (Diptera: Tephritidae), is an important pest of cultivated fruits throughout the Americas that can be controlled by natural enemies, such as the native parasitoid *Doryctobracon areolatus* (Szépligeti) (Hymenoptera: Braconidae) and the introduced parasitoid *Diachasmimorpha longicaudata* (Ashmead) (Hymenoptera: Braconidae). However, the control efficacy and parasitism performance of these organisms could be affected by changing environmental conditions. SDMs were conducted using Random Forest to predict suitable areas for the establishment of *A. fraterculus*, *D. areolatus*, and *D. longicaudata* under different climate scenarios or Representative Concentration Pathways (SSPs) (SSP 2-4.5 and 5-8.8) in two different periods (2021-2040 and 2041-2060). Our results predicted an increase in suitable areas for *A. fraterculus* in the Americas, especially in some South American countries such as Colombia and Brazil. Moreover, the projected distribution of these species is intricately linked to the regional climatic patterns. Where temperate and tropical areas were more suitable for the establishment of *A. fraterculus*, temperate climates were better for *D. areolatus*, and tropical

climates were more suitable for *D. longicaudata.* Suitable areas for the establishment of both parasitoid species were predicted to increase in future climate scenarios, with *D. longicaudata* having a greater geographical expansion than *D. areolatus*. These parasitoids could be used as biocontrol agents in almost all areas suitable for the establishment of *A. fraterculus*.

Keywords: South American Fruit Flies; Climate change; *Doryctobracon areolatus*; *Diachasmimorpha longicaudata*; SDM; Biological control.

Introduction

Climate change and extreme weather conditions are consequences of increasing concentrations of greenhouse gases in the atmosphere from human activity [1,2]. Therefore, increases in mean temperature, ~~less cold~~fewer occurrences of freezing temperatures, and increases in extreme precipitation are expected in some areas of the planet [3]. These phenomena affect not only the weather but also the distribution of species, including insect pests and their natural enemies. Increasing temperatures can directly affect insect reproduction, development, survival, and dispersal patterns [4]. Climate change is predicted to have a greater impact on insect natural enemies, such as parasitoids, than on insect pests themselves. The limited plasticity of natural enemies at high temperatures causes temporal desynchronization with their hosts and decreases suitable distribution areas for parasitoids [5–7]. Although increasing temperatures often increase parasitism rates to a certain level, after the optimum peak is reached, any increase in temperature reduces parasitism rates [8,9]. Therefore, climate change is critical to the success of biological control programs.

The recognized effect of climate change on the distribution of insects has led to the development of computational models for predicting pest risks in different areas [10]. These species distribution models (SDMs) use environmental variables and records of species occurrences to predict potentially suitable areas for species to grow and develop; these correlative models are commonly used to predict changes in biodiversity in various climate change scenarios [11]. Currently, some SDMs are being developed using machine learning algorithms, such as MaxEnt, Bioclim, and Random Forest [12–16]. The goal of these SDMs is to predict suitable areas for species in the current and future climate scenarios. This approach may help~~s~~ entomologists to predict the effects of climate change on agricultural pests and their natural enemies and can be used as a tool to determine appropriate areas for the release of parasitoids for pest mitigation [17–19].

The South American fruit fly *Anastrepha fraterculus* (Wiedemann, 1830) (Diptera: Tephritidae) is one of the most significant pests in the Americas and is distributed from Mexico to Argentina [20]. Currently, this species exhibit eight distinct morphotypes, as defined by Hernández-Ortiz et al. [21–23]: Andean, Brazilian-1, Brazilian-2, Brazilian-3, Ecuadorian, Mexican, Peruvian, and Venezuelan. The fruit fly *A. fraterculus* feeds on at least 160 crop species [24,25]. In Brazil, it is considered one of the primary pests affecting apple (*Malus domestica* Borkh.), peach [*Prunus persica* (L.) Bastch], and guava (*Psidium guajava* L.) [25,26]. In Colombia, *A. fraterculus* is a quarantine species reported feeding on coffee berries (*Coffea arabica* L.) in the Andean mountains at up to 1.100 MASL (meters above sea level) [27–29]. In Peru, this species is common in mango crops (*Magnifera indica* L.) [30,31]. In Mexico, this pest is particularly problematic for mango crops, which is a major agricultural product in many areas of the country [32].

One of the most common methods for controlling *Anastrepha* fruit flies involves the use of classical, augmentative, and conservative biological control techniques, utilizing native and exotic parasitoids [32–34]. Some of the more prevalent parasitoid species of *Anastrepha* sp. are *Doryctobracon areolatus* (Szépligeti, 1911) (Hymenoptera: Braconidae), native to South America, and *Diachasmimorpha longicaudata* (Ashmead, 1905) (Hymenoptera: Braconidae), native to Southeast Asia [35,36]. The parasitism rates of *D. areolatus* on *A. fraterculus* varied from 41.6~~0~~% to 68.6~~0~~% (53.5~~0~~% on average), demonstrating its potential in biological control programs [37,38]. Similar, the parasitism rates of *D. longicaudata* on *A. fraterculus* ranged from 12.9% to 62.9% when reared on citrus and guava fruits in laboratory conditions~~Similar parasitism rates of. *D. longicaudata* on *A. fraterculus* were found under laboratory contidions with rates up to 35.5% [39], and rates from 12.9% to 62.9% in citrus and guajaba fruits~~ ~~[40]~~and 35.5% in artificial diet~~.~~ [39,40]. Recently, the Brazilian Ministry of Agriculture and Livestock (MAPA) has registered *D. areolatus* as a fruit fly biocontrol agent for *Anastrepha* spp. [41].

The parasitoid *D. longicaudata* has been introduced in different countries in the Americas, including Colombia, Venezuela, Brazil, Costa Rica, Guatemala, El Salvador, Mexico, Nicaragua, Trinidad, and the United States of America (Florida) as a biological control agent of many tephritids [42,43]. This parasitoid has been found parasitizing *A. fraterculus* in Costa Rica, Mexico, Nicaragua, Guatemala, El Salvador, Trinidad, Colombia, and Venezuela [42]. Furthermore, augmentative releases of *D. longicaudata* in Mexico have resulted in up to 50% parasitism of *Ceratitis capitata* (Wiedemann, 1824) [44]. This parasitoid was also introduced to Argentina in the 1960s and 1990s to control *C. capitata* and *A. fraterculus* [45]. In Brazil, it was introduced in 1994 and officially registered as a biocontrol agent of tephritids in 2018 [33,46]. However, despite some releases in the states of Bahia and São Paulo in Brazil, where the parasitism

Formatted: Font: Not Italic, Highlight

Formatted: Font: Not Italic, Highlight

Formatted: Font: Not Italic, Highlight

Formatted: Font: Not Italic, Highlight

Formatted: Highlight

Formatted: Highlight

Formatted: Portuguese (Brazil)

rates reached values of 19.8% in citrus orchards [47], the parasitoid has not been successfully established speculatively due to intraspecific competition with native parasitoid species. *D. longicaudata* also failed to establish in the state of Rio Grande do Sul due to low temperatures in the region [43,48]. These studies suggest that *D. areolatus* and *D. longicaudata* require different ecological conditions for their successful establishment.

This study predicts the potential geographic distribution of the fruit fly *A. fraterculus* and its parasitoids *D. areolatus* and *D. longicaudata* across the Americas under current and future climate conditions. Additionally, this study forecasts potential population range shifts ~~in the future~~ using a comprehensive set of climate projection models, considering two shared socioeconomic pathways of gas emissions (SSP) and two distinct periods of time. We tested the following hypotheses: 1) the suitable areas for the establishment of the fruit fly *A. fraterculus* will increase in the future; 2) the suitable areas for the establishment of the parasitoids *D. longicaudata* and *D. areolatus* will not increase in the future*;* and 3) there will be less suitable areas for the introduced parasitoid *D. longicaudata* than for the native parasitoid *D. fraterculus* in future climate change scenarios. The results of this study contribute to the development of pest management programs for *A. fraterculus* by determining potential areas for the establishment of this pest and two of its parasitoid species in the Americas.

Materials and methods

Species record occurrences

The geographical coordinates of species occurrences used to build the SDM analysis for *A. fraterculus*, *D. longicaudata,* and *D. areolatus* were obtained using the "occ" function from the "spocc" RStudio package [49]. The occurrence records of these species were sourced from the following databases: The Global Biodiversity Information Facility (GBIF; https://www.gbif.org), The Vertebrate Network (VerNet, http://vertnet.org/index.html), iNaturalist (https://www.inaturalist.org/), and The Integrated Digitized Biocollections (iDigBio). Additional occurrences of *D. areolatus* were extracted from the records reported by Moreira et al. [50]. After filtering the occurrence recordsdata to remove duplicates, we obtained a total of 193 records for *A. fraterculus*, 21 for *D. longicaudata*, and 205 for *D. areolatus*. The coordinates for species presence records were saved in a ". cvs" file for further useThe georeferenced presence dataoccurrences for each species were then compiled and saved in a ".csv" file for subsequent analysis.

Both occurrences and absences are usually necessary to build SDMs. Nevertheless, due to the lack of true absence data in the databases, "pseudo-absence" locations were generated randomly to characterize the environmental background of the study area. To do this, we used the function "randomPoints" from the "dismo" package [51] in] in R statistical computing software v4.3.2; [50]-. These pseudo-absences are locations where it is assumed that there is no record or presence of the species, thus providing a point of comparison from those locations where the species has been recorded. In this study, a ratio of 2:1 pseudo-absence to presence locations was generated for each species, reducing over-fitting and maintaining the capacity of the model to discriminate between areas of presence and absence [53].

Formatted: Font: Italic, Highlight

Formatted: Font: Italic, Highlight

Formatted: Font: Italic, Highlight

Formatted: Font color: Text 1, Pattern: Clear

Environmental variables

SDMs use data on environmental conditions in which a species is known to exist to predict suitable habitats for that species. Environmental data for current and future climate conditions in the Americas were obtained from the WorldClim database (Global Climate Data; available at: https://www.worldclim.org/data/index.html) [54]. These data consist of 19 bioclimatic variables derived from temperature and precipitation in "raster" format (Table 1), at a spatial resolution of 2.5 arc-minutes (approximately 4.5 km).

Table 1. Bioclimatic variables.

Bioclimatic variables used for SDM (extracted from www.worldclim.org/data/bioclim.html**).**

Code	Variables
BIO1	Annual Mean Temperature
BIO2	Mean Diurnal Range (Mean of monthly (max temp - min temp))
BIO3	Isothermality (BIO2/BIO7) (×100)
BIO4	Temperature Seasonality (standard deviation ×100)
BIO5	Max Temperature of Warmest Month
BIO6	Min Temperature of Coldest Month
BIO7	Temperature Annual Range (BIO5-BIO6)
BIO8	Mean Temperature of Wettest Quarter
BIO9	Mean Temperature of Driest Quarter
BIO10	Mean Temperature of Warmest Quarter
BIO11	Mean Temperature of Coldest Quarter
BIO12	Annual Precipitation
BIO13	Precipitation of Wettest Month
BIO14	Precipitation of Driest Month
BIO15	Precipitation Seasonality (Coefficient of Variation)
BIO16	Precipitation of Wettest Quarter
BIO17	Precipitation of Driest Quarter
BIO18	Precipitation of Warmest Quarter
BIO19	Precipitation of Coldest Quarter

The current environmental conditions were downloaded from the WorldClim database version 2.1 and correspond to historical records from 1970 to 2000 [54]. The environmental conditions for future climate scenarios were obtained from 26 climatic projections or Global Climate Models (GCM) from the Coupled Model Intercomparison Project Phase 6 (CMIP6; available at https://www.worldclim.org/data/cmip6/cmip6climate.html, 52) (S1 Table). These projections consist of climatic predictions made by various modeling groups based on CO_2 emissions and mitigation effort scenarios up to the year 2100, called Shared Socioeconomic Pathways (SSPs). We selected two SSPs at two time periods, from 2021 to 2040 and from 2041 to 2060: (a) SSP2-4.5, which represents a stabilization scenario that assumes the implementation of climatic policies to mitigate greenhouse gas emissions [56]; and (b) SSP5-8.5, which corresponds to high greenhouse gas emissions pathway and is considered a baseline scenario without any specific targets for climate mitigation [57].

Suppression of collinearity in predictor variables

Collinearity, also known as multicollinearity, refers to the strong interdependence among explanatory variables typically observed in regression models. This phenomenon can lead to two primary issues: inflated estimates of variable effects and uncertainty in model extrapolation [58]. The most common approach for addressing collinearity issues is a priori variable selection, which involves either excluding the most correlated variables or combining them into new explanatory terms [59]. For this purpose, each species occurrence datum was projected into the current environmental conditions database, and the values of each bioclimatic variable at those points were

extracted. Subsequently, the Variance Inflation Factor (VIF) was calculated using the “vifstep” function from the “usdm” package [60]. This test excluded the highly correlated variables with VIF values >10; the remaining variables were used to run the SDMs projections.

Modeling and projections

Random Forest (RF) is a powerful machine-learning method for building SDMs and training models with small sample sizes [61–63]. This method consists of hundreds of trees constructed using random samples from the databases of occurrences/pseudo-absences and selected bioclimatic variables (predictors). Each tree is built from a bootstrap sample, which is a random sample with replacement from the original dataset. At each node of the tree, the best predictor is selected from a small, randomly chosen subset of the predictor variable pool. This process helps introduce randomness and reduce overfitting of the model [64]. The final prediction is then made by aggregating the predictions of all individual trees. This ensemble approach tends to improve the robustness and generalization performance of the model compared to the use of a single decision tree [65].

RF involves several parameters that control the structure of each tree, the overall structure and size of the forest, and its randomness properties. For example, the number of randomly drawn candidate variables (mtry), the node size, and the number of trees (ntree). The optimal selection of these parameters significantly influences model prediction accuracy [66]. To determine the best values for these parameters, strategies such as "Tuning" have been developed [67]. In this context, parameter selection was performed using the "train" function from the "caret" package [68]. For all predictions made for *A. fraterculus* and *D. areolatus*, a ntree of 2000, a mtry of 3, and a node size of 2 with 25 replications were selected. However, for all predictions involving *D.*

longicaudata, a ntree of 2500, a mtry of 3, and a node size of 2, with 10 replications were selected. Subsequently, all the models were adjusted using 75% of the occurrence data to train the model and the remaining 25% for testing purposes. An ensemble was created using all replications for the current projection. For future projections, an ensemble was formed by combining all 25 Global Climate Models (GCMs) for each climatic period (2021-2040 and 2041-2060) and Representative Concentration Pathway (SSP).

SDMs using RF usually provide numerical predictions in the range of 0–1. However, to make the data more practical, it is essential to establish a threshold for interpreting the predictions using levels of suitability and transforming them into binary data indicating species presence or absence. Suitability scores higher than the threshold value are considered suitable areas for the establishment of a given species. In this study, threshold values were obtained for each insect species by extracting all the Receiver Operating Characteristic (ROC) curve data using the function "roc" of the "pROC" package [69]. Subsequently, the threshold was determined as the mean of all the threshold values in each replication for each species (25 for *A. fraterculus* and *D. areolatus*, and 10 for *D. longicaudata*). We used four suitability levels represented on a color gradient from green to red on the plotted maps: a) highly suitable areas depicted in green color correspond to suitability values close to 1; b) moderately suitable areas illustrated in yellow designate suitability values close to 0.5; c) low suitable areas depicted in red show suitability values close to the threshold for each species (0.08 for *A. fraterculus*, 0.31 for *D. areolatus* and 0.15 for *D. longicaudata*); and d) unsuitable areas represented by the absence of color correspond to values below the threshold and close to 0.

Lastly, the suitability values for each predicted scenario and period were converted into binary values using a species-specific threshold. This transformation was conducted for two key

p~~ro~~urposes: 1) to quantify the suitable and unsuitable areas in square kilometers for all species, facilitating the assessment of expansion areas across different countries in the Americas; 2) to generate maps illustrating the overlap of suitable areas between each parasitoid and the pest *A. fraterculus*. For the first one, we utilized the "expanse" function from the "terra" package [70]. The resulting data were subsequently saved in a ".csv" file. To aid interpretation, the top 10 countries with the largest suitable areas for the fruit fly *A. fraterculus* were selected. The same countries were also chosen for quantifying the suitability areas of the parasitoids *D. areolatus* and *D. longicaudata*, and the data were visualized using a bar plot.

All the maps generated illustrating the suitable areas for each species, as well as the overlap of suitable areas between the parasitoids and pest, were generated using the function “ne_countries” of the “rnaturalearth” RStudio package [71].

Model performance

To assess the performance of the models, we used the Receiver Operating Characteristic (ROC) curve analysis, specifically examining the area under the curve (AUC) [72]. The AUC value is a widely accepted metric for evaluating the accuracy of SDMs. Generally, AUC values below 0.7 indicate poor performance, those between 0.7 and 0.9 suggest moderate performance, and values exceeding 0.9 indicate good performance [73]. For each species, the relative importance of the selected bioclimatic variables was determined through analysis of multicollinearity and AUC metrics. This comprehensive approach allowed us to gauge both the predictive power of the models and the significance of individual variables in the SDMs.

Results

The predicted SDMs constructed for *A. fraterculus*, *D. areolatus*, and *D. longicaudata*, had good performance with AUC values of 0.995 ± 0.005, 0.963± 0.034, and 0.9583 ± 0.1317 respectively. These models indicate that suitable areas for the pest *A. fraterculus* and the parasitoids *D. areolatus* and *D. longicaudata*, were predicted to increase in the Americas in all the scenarios and periods evaluated (Table 2). The species with the largest suitable areas ~~Among the three species analyzed, the parasitoid *D. longicaudata* showed the largest extension of suitable areas~~ in each ~~all the predictions~~prediction (SSPs scenario and periods) was *D. longicaudata*, estimated to occupy~~ing~~ up to 38~~6~~.06~~54~~% of America's territory, followed by *A. fraterculus* with 36.17%, and *D. areolatus* with 29.66% (Table 2). The predicted occupancy areas had ~~Additionally,~~ n~~n~~o marked differences ~~were found~~ between the climatic scenarios analyzed (Table 2).

Table 2. **Percentage of predicted suitable areas for the establishment of the pest *Anastrepha fraterculus*, and the parasitoids *Doryctobracon areolatus* and *Diachasmimorpha longicaudata* in the Americas in two Shared Socioeconomic Pathways (SSPs) at two periods.**

Species		SSP2-4.5		SSP5-8.5	
	Current	2021-2040	2041-2060	2021-2040	2041-2060
Anastrepha fraterculus	31.39%	34.56%	35.55%	34.58%	36.17%
Diachasmimorpha longicaudata	35.07%	36.66%	37.42%	36.79%	38.06%
Doryctobracon areolatus	21.53%	26.25%	28.37%	26.59%	29.66%

Among the bioclimatic variables analyzed, Isothermality (BIO3) had the highest contribution to the *A. fraterculus* SDM with 20.51%, followed by the mean temperature of the driest quarter (BIO9) with a contribution of 14.22%, and the precipitation of the warmest quarter (BIO18) with 13.52% (Table 3). For *D. areolatus* SDM, the bioclimatic variable with major contribution was the annual temperature range (BIO7) with 25.35%, followed by isothermality (BIO3) with a contribution of 16.20%, and precipitation of the wettest month (BIO13) with 12.05%. For *D. longicaudata*, two main variables contributed more than 70% to the SDM model: the average temperature of the coldest quarter (BIO11) with 51.02% and the precipitation of the wettest quarter (BIO18) with 27.33% (Table 3).

Table 3. **The relative contribution of the bioclimatic variables for Random Forest models (RF) built for *Anastrepha fraterculus*, *Doryctobracon areolatus*, and *Diachasmimorpha longicaudata.***

Species	Codes	Bioclimatic variables	Contribution (%)
Anastrepha fraterculus	BIO2	Mean Diurnal Range	10.02
	BIO3	Isothermality	20.51
	BIO8	Mean Temperature of Wettest Quarter	11.02
	BIO9	Mean Temperature of Driest Quarter	14.22
	BIO13	Precipitation of Wettest Month	7.70
	BIO15	Precipitation Seasonality	11.54
	BIO18	Precipitation of Warmest Quarter	13.52
	BIO19	Precipitation of Coldest Quarter	11.45
Dory ctobr	BIO3	Isothermally	16.20

	BIO7	Temperature Annual Range	25.35
	BIO8	Mean Temperature of Wettest Quarter	8.22
	BIO10	Mean Temperature of Warmest Quarter	9.88
	BIO13	Precipitation of Wettest Month	12.05
	BIO14	Precipitation of Driest Month	6.04
	BIO15	Precipitation Seasonality	4.57
	BIO18	Precipitation of Warmest Quarter	10.79
	BIO19	Precipitation of Coldest Quarter	6.89
Diachasmimorpha longicaudata	BIO 11	Mean Temperature of Coldest Quarter	52.02
	BIO15	Precipitation Seasonality	4.36
	BIO16	Precipitation of Wettest Quarter	27.33
	BIO18	Precipitation of Warmest Quarter	11.83
	BIO19	Precipitation of Coldest Quarter	4.46

The *Anastrepha fraterculus* SDM

Our models predict that *A. fraterculus* has an extensive suitable area for establishment in South America, with Brazil exhibiting the largest extent (up to 5 million km²), followed by Peru (up to 1 million km²) and Colombia (up to 0.8 million km²) (Fig 1). In Brazil, the current time projected suitable areas occupy 61% of the national territory, with a potential increase of up to 75% under future climate scenarios (S1 Table). These predicted suitable areas were predominant in the southern states (Rio Grande do Sul, Santa Catarina, Paraná), southeastern states (São Paulo,

Minas Gerais, Rio de Janeiro, Espírito Santo), and parts of Bahia State in Brazil (Fig 2). In Peru, the projected suitable areas for *A. fraterculus* extend up to 98% of the country, with minimal variation in future projections (S1 Table), these suitable areas were predicted in the Andean mountains and adjacent valleys (Fig 2). Conversely, Colombia is projected to experience a significant expansion of suitable areas, from 70% to 99% of its territory under future scenarios (Fig 1), with new suitable areas emerging in the ~~Orinoquía~~Plains (East Colombia) and Amazon (South Colombia) regions, showing moderate to low suitability for *A. fraterculus* (Fig 2).

Fig 1. Top 10 countries with the largest suitable areas (Km^2) for *Anastrepha fraterculus*. ~~Extension of Ssuitable areas p~~Predicted suitable areas for the parasitoids *Diachasmimorpha longicaudata* and *Doryctobracon areolatus* and the pest *A. fraterculus* in current and future time predictions (SSP2-4.5, SSP5-8.5) (2021-2040, 2041-2060.

Fig 2. Suitable areas predicted for *Anastrepha fraterculus* in the Americas. (A). Suitable areas predicted in the current time period*;* (B-E~~D~~). Suitable areas predicted under two future climate change scenarios (SSP2-4.5 and SSP5-8.5) (periods 2021-2040 and 2041-2060) ;(B) scenario SSP2-4.5 time period 2021-2060; (C) scenario SSP2-4.5 time period 2041-2060; (D) scenario SSP5-8.5 time period 2021-2040; (E) scenario SSP5-8.5 time period 2041-2060. The maps show suitability values in a green to-red color where: high suitable areas depicted in green color correspond to suitability values close to 1; moderately suitable areas illustrated in yellow designate suitability values close to 0.5; low suitable areas depicted in red show suitability values close to

the threshold the species (0.08 for *A. fraterculus*); unsuitable areas represented by the absence of color correspond to values below the threshold and close to 0.

In North America, current time predictions indicate suitable areas for *A. fraterculus* along the east coast of the United States (0.9 million km²) and in Mexico (0.8 million km²) (Fig 1), particularly in the coastal states of Yucatán, Campeche, Chiapas, Oaxaca, and Tabasco. In the United States the suitable areas for this pest are predicted to increase in future climate scenarios by 12% of its territory concentrated in the states of Ohio, Indiana, and Kentucky (Fig 2). In contrast, Mexico was projected to experience a slight decline of suitable areas for *A. fraterculus* from 39% to 36% of its territory in future scenarios (S2 Table). The Mesoamerican countries such as Panama, Costa Rica, Guatemala, and Honduras display low to high suitability across nearly all their territories in both current and future projections, with minimal temporal variation (Fig 2).

The fruit fly parasitoid, *Doryctobracon areolatus* SDM

Moderate to high suitable areas for *D. areolatus* were identified across South America, particularly along the western and coastal regions of Brazil, as well as in northern Venezuela, Paraguay, Colombia, and Bolivia (Fig 3). In contrast, areas with low suitability were predicted in the Amazon region and some areas in the Andes mountains, spanning Chile, Peru, Ecuador, and Colombia. Additionally, some areas were classified as low to moderate suitable for the establishment of *D. areolatus* in Central and North America (Fig 3).

Fig 3. Suitable areas predicted for *Doryctobracon areolatus* in the Americas. (A) Suitable areas predicted in the current time period; (B-~~D)~~E Suitable areas predicted under two future climate change scenarios (SSP2-4.5 and SSP5-8.5) (periods 2021-2040 and 2041-2060); (B) scenario SSP2-4.5 time period 2021-2060; (C) scenario SSP2-4.5 time period 2041-2060; (D) scenario SSP5-8.5 time period 2021-2040; (E) scenario SSP5-8.5 time period 2041-2060. The maps show suitability values in a green to-red color where: high suitable areas depicted in green color correspond to suitability values close to 1; moderately suitable areas illustrated in yellow designate suitability values close to 0.5; low suitable areas depicted in red show suitability values close to the threshold the species (0.31 for *D. areolatus*); unsuitable areas represented by the absence of color correspond to values below the threshold and close to 0.

In Brazil, current projections estimate that 69% of the territory is suitable for the establishment of *D. areolatus*, with future climate scenarios predicting an increase of up to 89% (Fig 1). In Peru, suitability is projected to increase from 16% to 36%, and in Colombia, from 26% to 56% (Fig 1), mainly in the ~~Orinoquia~~ Plains and Amazon regions (Fig 3). In Mexico, the current suitable areas predicted for *D. areolatus* are concentrated in the southeast, southwest, and central-south (Fig 3), with future projections suggesting a 10% increase (Fig 1; S2 Table). In the United States, suitable areas were consistently identified along the Southeast Coast, particularly in Florida, under all projections (Fig 3; S2 Table).

Overlapping suitable areas between *A. fraterculus* and *D. areolatus*, were projected in the south and littoral region of Brazil, in the north of Colombia and Venezuela, and in the majority of Mesoamerica (Fig 4). In Mexico, overlapping suitable areas were more predominant in the

Yucatan peninsula and in the south part of the country (Fig 4). In contrast, in the United States of America overlapping areas were restricted to the south of Florida (Fig 4). Future projections, show an increase of overlapping suitable areas between *A. fraterculus* and *D. areolatus* in almost all Brazilian territory, except in some areas in the ~~amazon~~ Amazon region and the center region of Brazil (Fig 4). However, In Colombia, common suitable areas between both *A. fraterculus* and *D. areolatus*, were predicted in the ~~amazon~~ Amazon and ~~Orinoquia~~ the Plains ~~valley~~ (Fig 4). In contrast, in Mexico and the United States of America, common suitable areas for the pest and the parasitoid had no significant change in future predictions (Fig 4).

Fig 4. Overlapping suitable areas predicted for *Doryctobracon areolatus* and *Anastrepha fraterculus* in the Americas. (A) Overlapped suitable areas predicted in the current time period; (B) Overlapped suitable areas predicted in the future 2041-2060 scenario SSP5-8.5.

The fruit fly parasitoid, *Diachasmimorpha longicaudata* SDM

Our predictions showed that suitable areas for *D. longicaudata* occupy 35.07% of the Americas territory (Table 2). Future projections indicate a slight expansion of suitable areas of up to 38.06% in the Americas (Table 2). Highly to moderate suitable areas for *D. longicaudata* were projected in the Center-West and North of Brazil, with an increase in suitable areas of 14 % in its territory in future predictions (Fig 5, S2 Table). In Peru, suitable areas for the current period were predicted to cover 63% of the national territory, which could increase to 67% in future scenarios (Fig 1; S2 Table). In Colombia, future projections indicate that up to 90% of the country will be

suitable for *D. longicaudata* (Fig 1; S2 Table), with moderate to high suitability across most regions, except for the Andean mountains, which remain unsuitable in future predictions (Fig 5.). In the United States, suitable areas are limited to central and southern Florida, with no significant expansion observed in future scenarios (Fig 5). Across Mesoamerica, future projections suggest moderate suitability for *D. longicaudata* along coastal areas and low suitability in the Northeast, lowlands, and central regions of Mexico (Fig 5). Suitable areas in Mexico are expected to increase 17% in future projections.

Fig 5. Suitable areas predicted for *Diachasmimorpha longicaudata* in the Americas (A) Suitable areas predicted in the current time period; (B-~~D)~~E Suitable areas predicted under two future climate change scenarios (SSP2-4.5 and SSP5-8.5) (periods 2021-2040 and 2041-2060); (B) scenario SSP2-4.5 time period 2021-2060; (C) scenario SSP2-4.5 time period 2041-2060; (D) scenario SSP5-8.5 time period 2021-2040; (E) scenario SSP5-8.5 time period 2041-2060. The maps show suitability values in a green to-red color where: high suitable areas depicted in green color correspond to suitability values close to 1; moderately suitable areas illustrated in yellow designate suitability values close to 0.5; low suitable areas depicted in red show suitability values close to the species threshold (0.15); unsuitable areas represented by the absence of color correspond to values below the threshold and close to 0.

Notably, there is a substantial overlap of suitable areas for *D. longicaudata* and *A. fraterculus*, particularly in South America and Mesoamerica (Fig 6). Current predictions showed suitable overlapping areas across the Amazon region in Brazil, Peru, and Colombia; these areas

are predicted to have a significant expansion in future projections (Fig 6). In Mexico, overlapping suitable areas for the establishment of *D. longicaudata* and *A. fraterculus* were predicted along the coast in both current and future predictions (Fig 6). ~~While i~~In the United States of America, overlapping suitable areas for the parasitoid and the pest were predicted only in the state of Florida (Fig 6).

Fig 6. Predicted overlapping suitable areas for the establishment of *Diachasmimorph longicaudata* and *Anastrepha fraterculus* in the Americas. (A) Overlapping suitable areas predicted in the current time period; (B) Overlapping suitable areas predicted in the future 2041-2060 scenario SSP5-8.5.

Discussion

Anastrepha fraterculus is predicted to expand to new territories in the Americas under future climate change scenarios

Our models predict a reduced ability of *A. fraterculus* to establish in warmer, dry regions, and a high ability to establish in temperate areas (Fig 2). These predictions agree with previous laboratory bioassays that found a reduced emergence of *A. fraterculus* adults at temperatures exceeding 30°C, whereas the emergence rate exceeded 70% at 15 °C [74]. Similarly, Santos [75] concluded that water stress due to scarcity can cause mortality of *A. fraterculus* under warmer conditions. Our results are comparable with those reported for *A. grandis* SDM, where the precipitation of the warmest quarter (BIO10) was the most influential variable, suggesting that

extreme drought events may drive the future distribution of this species [76]. Also, in our predictions, regions with temperate climates showed moderate and high suitability for the establishment of *A. fraterculus*~~.~~, whereas low suitable and unsuitable areas include some tropical climate regions. Based on this, we suggest that temperate climate areas with high humidity seem to be more suitable for the establishment of this species at the current time and in the future. Indeed, the spread of *A. fraterculus* in temperate climate regions due to climate change has already been reported in the field [77].

In Brazil, temperate climates are predominant in the southern and southeastern regions and are characterized by hot summers, no dry seasons, and cooler winters [78]. However, our study found suitable areas for the establishment of *A. fraterculus* in the coastal region characterized by high temperature, low altitude, and high humidity [79]. Our projections concur with previous studies reporting the presence of different *A. fraterculus* morphotypes in these regions. For example, the Brazilian-2 and Brazilian-3 morphotypes seem to be more adapted to coastal areas, whereas the Brazilian-1 morphotype seems to thrive in temperate areas [22,80,81]. Our model predicted areas with low or no suitability for *A. fraterculus* in the Brazilian Amazon region and central-western states. These regions experience tropical climates, characterized by dry winters and summers, whereas the Brazilian Amazon has predominantly tropical monsoon and tropical rainforest climates [78,82]. The low suitability predicted in these areas might be the result of not conducting separate analyses for each *A. fraterculus* morphotype. SDMs developed by Selivon et al. [81] for each *A. fraterculus* morphotype in Brazil, found suitable areas only in the southeast and south of the country. On the other hand, populations of *A. fraterculus* in Mesoamerica, known as the ‘Mexican morphotype’, were found at low elevations (30 – 1,400m) and thrive in both temperate and tropical climates [22,23]. Therefore, our results of the expansion of the suitable

regions for *A. fraterculus* in the Brazilian Amazon are for the species without differentiation between its morphotypes. However, the continued presence of *A. fraterculus* in future scenarios in the Brazilian southern and southeastern could pose a risk for ~~some~~ crops, such as apples and peaches in these regions [26,83].

The Peruvian mountains, predicted by our study as suitable for *A. fraterculus*, are characterized by two types of climates, temperate and arid [78]. A study conducted by Ramos Peña et al. [84] in the Abancay Valley in Apurimac, Peru, found an increase in fruit fly populations during the rainy season and the transitions to the dry season. The authors also suggested that low temperatures and the availability of suitable hosts increase fruit fly populations. Studies conducted in guava orchards in Oxapampa, Peru, revealed an increase in the abundance and uniform distribution of *A. fraterculus* at higher elevations. Moreover, these trends appear to be influenced by both seasonality and altitude [85]. In Colombia the presence *A. fraterculus* in coffee crops in the Andean mountains was previously reported by Jaramillo et al. [28], representing 99.9% of all specimens of fruit flies in coffee berries; however, its effects on fruit development and coffee quality are unknown. Additionally, at least 37 other hosts for *A. fraterculus* have been reported in Colombia [86]. Some of these hosts are important crops in the ~~Orinoquia~~ Plains and Amazon regions and will be at risk in the future, such as mango [87,88], soursop (*Annona muricata* L.), papaya (*Carica papaya* L.) [87], and tangerine (*Citrus reticulata* Blanco) [27]. Given the potential increase of suitable areas in the ~~Orinoquia~~ Plains and Amazon regions of Colombia in the coming years, efforts to monitor the dispersion of *A. fraterculus* are highly recommended.

Our results show suitable areas for *A. fraterculus* in the Yucatan peninsula in Mexico, where this pest is currently present [23]. The loss of suitable areas predicted by our models in Mexico can be explained by a reduction of temperate climate regions, transitioning to arid climates

in the future [78]. This suggests that arid climates are not optimal for the establishment of the species. Indeed, states such as Chihuahua, Durango, Coahuila, Nuevo Leon, and Zacatecas, characterized by arid climates, were found unsuitable for *A. fraterculus* in all our predictions. The presence of *A. fraterculus* in the United States was previously reported in southern Texas, where the species appears to be restricted [89]. However, our future projections suggest that this pest can spread to new suitable habitats. Therefore, despite the low suitability of the new regions for the establishment of *A. fraterculus*, it remains crucial to monitor the potential expansion of this pest in the U.S.

Overall, our results predict an expansion in the suitable areas for *A. fraterculus* in temperate regions and some tropical areas across the Americas in the coming years. These predictions support our first hypothesis about the pest's potential distribution. The models suggest this expansion could occur despite some regions becoming less suitable due to climate change. However, additional studies will be needed to determine if the predicted suitable areas for this pest overlap with compatible areas for the cultivation of susceptible crops. Our continental-scale projections provide valuable insights into the pest distribution, but we recognize that local microclimates, agricultural practices, and the presence of different pest morphotypes are essential to determine the establishment of this pest. Nevertheless, monitoring efforts should prioritize regions identified as suitable in our models, especially if valuable host crops are cultivated in those areas.

In light of the evidence, suitable areas for *A. fraterculus* could expand its range into temperate regions and certain tropical areas across the Americas in the coming years. These findings support our first hypothesis about the pest's potential distribution. The models suggest this expansion could occur despite some regions becoming less suitable due to climate change. Future studies focused in how these shifting patterns may bring the pest into contact with important crops could be

Formatted: Font: Not Italic, Highlight

Formatted: Indent: First line: 0"

Formatted: Font: Italic

necessary to be developed. While our continental-scale projections provide valuable insights, we recognize that local factors including specific microclimates, agricultural practices, and the presence of different morphotypes, could be essential to determine where and how severely this pest establishes itself. However future monitoring efforts should prioritize regions identified as suitable in our models, especially where valuable host crops are cultivated.

The fruit fly parasitoid, *Doryctobracon areolatus* as a promising control agent in temperate regions

Suitable areas for *D. areolatus* are predicted in arid and temperate regions, while tropical climates and high-altitude regions seem to be unsuitable or low-suitable for the establishment of this species. Similar results were reported by Núñez-Campero et al. [90], who built SDM for *D. areolatus* in the current period and found that this species is associated with semiarid conditions while high humidity decreases suitability for its establishment. In their projections, precipitation of the driest quarter (BIO17) and precipitation of the coldest quarter (BIO19) were the variables with the greatest contribution to the model with values of 18% and 33%, respectively. Nonetheless, in our models, the bioclimatic variables associated with temperature and precipitation had the highest values of contribution values; this discrepancy could be related to different methodologies to construct the models and to different geographic areas of study. However, previous studies demonstrate that the best thermal conditions for the growth of *D. areolatus* are between 22 and 25 °C [91]. Also, studies conducted at different elevations have demonstrated that the parasitism rates of *D. areolatus* are significantly reduced at high altitudes, supporting our results.

In Brazil, certain areas classified by Beck et al. [78] as tropical rainforests in the northern regions of the country were predicted as low suitable or unsuitable for *D. areolatus*, particularly

in the Amazonas state (Fig 3). The limited suitability of this parasitoid species in tropical rainforest regions can be attributed to the high precipitation levels in these areas, which affect the population dynamics and reduce the parasitism capacity [5,92]. Our current and future projections indicate that the temperate zones in southern and southeastern Brazil are more suitable for the establishment of *D. areolatus*. These regions are also suitable for the establishment of *A. fraterculus*, suggesting that *D. areolatus* could be a good biological control agent for this pest. For example, in the state of Rio Grande do Sul, the parasitoid was found in association with *A. fraterculus*, exhibiting parasitism rates between 8% and 25% [93,94].

Moreover, overlapping suitable areas for *A. fraterculus* and *D. areolatus* were predicted in other countries across Latin America (Fig 4). For instance, in Peru, Colombia, Venezuela, Ecuador, and Bolivia, the parasitoid *D. areolatus* has been found in association with *A. fraterculus* [20,95]. In Peru, some suitable areas for *D. areolatus* were predicted in the "la Sierra" region, which is heavily planted with fruit crops and is characterized in part by a temperate climate [78,96]. Additionally, a study conducted by Salazar-Mendoza et al. [97] in Oxapampa, Pasco (low elevation in la Sierra region), found 11.76% of *A. fraterculus* parasitized by *D. areolatus* in níspero (*Eriobotrya japonica* Lindl.). However, our predictions for future climate scenarios show a low suitability of these regions for the establishment of the parasitoid *D. areolatus*. Therefore, we suggest the use of other biocontrol agents to manage *A. fraterculus* in these regions in the future.

Moderately and highly suitable areas in current and future projections were predicted in the Valley of the Andean Colombia, specifically in the departments of Huila, Valle del Cauca, and Tolima (Fig 3). The presence of *D. areolatus* in the department of Valle del Cauca was previously reported by Carrejo and González [98] preying on *Anastrepha obliqua* (Macquart, 1835) in mango crops. Also, *D. areolatus* in Colombia seems to be more common in areas of low elevations [99],

which coincides with our predictions of moderate to low suitability in the Atlantic Coast and Plains regions of Colombia. Therefore, the parasitoid *D. areolatus* could help mitigate the effect of *A. fraterculus* and other *Anastrepha* species in crops planted in low-altitude areas.

In Mexico, we found suitable areas for *D. areolatus* in regions characterized by a low elevation and a tropical savannah climate [78]. Previous studies reported that *D. areolatus* was the most predominant parasitoid across various host species of *Anastrepha* in the states of Vera Cruz and Chiapas [100]. Also, Sivinski et al. [101] found that *D. areolatus* was more frequent in low altitudes in the state of Vera Cruz, which supports our findings.

In the United States of America, the parasitoid *D. areolatus* was introduced in 1969 in Florida to control the Caribbean fruit fly, *Anastrepha suspensa* (Loew, 1862) [102]. This parasitoid was successfully established in Florida except in the southern part of the state. Eitam et al. [103] pointed out that the absence of *D. areolatus* in this region can be attributed to competitive displacement due to the introduction of *D. longicaudata*. These authors also found that *D. areolatus* was more common in sites with lower mean temperatures, where greater seasonality and less homogeneous host distribution favor this species.

~~As we discuss~~Collectively, our results suggest that the native parasitoid *D. areolatus* ~~demonstrates potential as a~~is a promising biological control agent against *Anastrepha* fruit flies, particularly in temperate and arid regions, where its establishment and parasitism rates are highest. Tropical and high-altitude areas seem to be unsuitable or low-~~-~~suitable due to excessive humidity and cooler temperatures, a trend reinforced by future climate projections. The suitable areas for the establishment of *D. areolatus* are predicted to increase between 4.72 and 8.13% in future climate change scenarios, contradicting our second hypothesis of no expansion in suitable areas for this species. ~~However, increasing suitable areas for *D. areolatus* in climate change projections,~~

Formatted: Font: Italic, Highlight

Formatted: Font: Italic, Highlight

Formatted: Font: Not Italic, Highlight

Formatted: Font: Not Italic, Highlight

Formatted: Font: Not Italic, Highlight

Formatted: Font: Not Italic, Highlight

Formatted: Font: Italic, Highlight

~~allows us to reject our second hypothesis.~~ This ~~demonstrate~~suggests that climate change could expand suitable areas also for pests' parasitoids, ~~witch~~ reinforcinge the viability of biological control programs in the future.

Formatted: Indent: First line: 0"

The fruit fly parasitoid, *Diachasmimorpha longicaudata* as a promising control agent in tropical regions

According to our projections, highly and moderately suitable areas for *D. longicaudata* have tropical climates, including areas characterized as tropical rainforests like the Amazon region. ~~While,~~ L~~l~~ow suitability or unsuitability is~~is~~ observed in areas with temperate or arid climate, such as the South of Brazil, Paraguay, along the Andean mountains, and certain states in Northwest and Northeast Mexico (Fig 4). Previous studies demonstrated that the optimum temperature for the development of immature stages and fecundity of *D. longicaudata* is between 25 ºC and 27 ºC, with complete development at 30 ºC in the hosts *Bactrocera dorsalis* (Handel, 1912) and *C. capitata* [104–106]. In another study developed by Meirelles et al. [104], regions with an average temperature higher than 25 ºC were more favorable for establishing *D. longicaudata* as a control agent in the field. Also, according to Harbi et al. [105], temperatures between 16 ºC and 24 ºC and relative humidity between 45% and 60% are ideal for parasitism by *D. longicaudata*. According to the same authors, a decrease in relative humidity reduces parasitism rates, while an increase in temperature increases parasitism. These findings collectively suggest that tropical regions provide more suitable conditions for the successful establishment and performance of *D. longicaudata*.

In Northern Brazil, areas suitable for *D. longicaudata* overlap with those of the pest *A. fraterculus* (Fig 2 and 4). However, this tropical region has low suitability for the pest, suggesting

that *D. longicaudata* could be a good biological control agent of other tephritids, such as *Bactrocera carambolae* (Drew & Hancock, 1994) (Diptera: Tephritidae) in these zones [107]. Moderately suitable areas were also identified in Southeast Brazil, particularly in São Paulo, where *D. longicaudata* could aid in tephritid control in citrus orchards. Paranhos et al. [33] reported that summer releases enhance parasitism, while winter conditions allow high survival rates despite heavy rainfall. In contrast, a low adaptation of *D. longicaudata* was observed before in the state of Amapá in ~~the~~ North Brazil ~~in the state of Amapá~~ [108], which ~~aligning~~ aligns with our current projections ~~with~~ for some areas classified as low suitable. However, future predictions indicate that Southeast and North Brazil may become moderately to highly suitable for the establishment of this parasitoid, suggesting that *D. longicaudata* could be used in forthcoming biological control programs of tephritid fruit flies. In the south of Brazil, previous studies developed in Rio Grande do Sul, demonstrated that the parasitoid has limited tolerance to winter, leading to ~~reduced~~ a smaller number of generations [104]. Therefore, other biocontrol agents that are better adapted to the environmental conditions of southern Brazil, such as *D. areolatus*, may be better options for controlling *A. fraterculus*.

In Peru, unlike *D. areolatus*, high and moderately suitable areas were projected across the entire region of "la Selva" and low suitable areas in almost the entire region of "la Sierra" (Fig 4). This suggests that the parasitoid *D. longicaudata* could be an important biocontrol agent for *A. fraterculus* in Peru's fruit-growing regions. Similarly, in Colombia, *D. longicaudata* may be an effective biological control agent of *A. fraterculus* particularly in tropical regions that are predicted to be unsuitable or low suitable for the establishment of *D. areolatus.*

In future scenarios, suitable areas for the establishment of *D. longicaudata* were predicted across all Mesoamerica (Fig 5). The coastal regions of Mexico characterized by a tropical climate

were predicted as suitable for the establishment of *D. longicaudata*, while the temperate and arid regions of Northeast, and Central Mexico were unsuitable. Our predictions indicate an expansion of suitable areas for *D. longicaudata* in ~~in~~ future climate change scenarios, which can be attributed to a projected shift in certain Mexican regions from temperate to tropical [78]. This change could benefit the expansion of *D. longicaudata* populations in the region. Some suitable areas for *D. longicaudata* ~~,~~ in Mexico, such as the southern of Chiapas cultivated with mango~~,~~ exhibit parasitism rates between 0.44% and 29.23% on other *Anastrepha* species [109]. Augmentative biological control of *Anastrepha* species in this region has shown that *D. longicaudata* can reduce pest populations without negatively affecting parasitoid local richness [110,111]. Therefore, *D. longicaudata* is a promising biocontrol ~~control~~ agent for *A. fraterculus* and other *Anastrepha* species in the tropical climate areas in Mexico.

In the United States, *D. longicaudata* was introduced in 1988 to control *C. capitata* in Florida.~~,~~ T~~t~~his~~e~~ parasitoid reduced the fruit fly populations and is now established in the region [103,112]. The distribution of *D. longicaudata* proposed a decade ago aligns closely with the suitable areas predicted by our model [103]. According to our results, both *D. areolatus* and *D. longicaudata* could be used for the control of *A. fraterculus* in the State of Florida (Fig 4 and 6). However, if the pest spreads to other northern states in the future, where conditions are unsuitable for both parasitoids according to our predictions, the use of alternative control tactics or biocontrol agents may become necessary.

In summary, suitable habitats for *D. longicaudata* may expand in tropical regions under future climate scenarios, ~~leading us to reject~~contradicting our second and third hypotheses. This suggests that non-native parasitoids like *D. longicaudata* could occupy a broader ~~climatic~~geographical range than native species under climate change. Importantly, *D. longicaudata* could serve as an

Formatted: Font: Italic, Highlight

Formatted: Font: Italic, Highlight

Formatted: Font: Italic, Highlight

effective biocontrol agent against fruit flies, particularly in areas where *D. areolatus* is unsuitable, thereby nearly covering the entire potential range of the pest. ~~Although our SDMs performed well with limited data, increased occurrence records from biodiversity surveys would improve predictive reliability. Importantly, D. longicaudata could serve as an effective biocontrol agent against fruit flies, particularly in areas where *D. areolatus* is unsuitable, thereby nearly covering the entire potential range of the pest.~~

~~This study highlights the importance of incorporating biological control strategies into integrated pest management programs targeting *A. fraterculus*.~~

Formatted: Font: Italic, Highlight

Formatted: Font: Italic, Highlight

Formatted: Font: 12 pt, Not Bold

Conclusions

Our study highlights the potential expansion of the South American fruit fly *Anastrepha fraterculus* in the future under climate change, with a potential increase in fruit damage in countries like Brazil and Colombia. Suitable areas for the establishment and dispersion of this pest in the Americas were mostly associated with temperate and some tropical climate regions. Our findings also suggest an increase of suitable areas for the establishment of the parasitoids *D. areolatus* and *D. longicaudata*. The exotic parasitoid *D. longicaudata* could have a greater geographical expansion than *D. areolatus* under future climate change scenarios. Suitable areas for the establishment of *D. longicaudata* were more common in tropical climate regions, especially in tropical rainforests, whereas suitable areas for the establishment of *D. areolatus* were more common in temperate climates. Due to the ability of these parasitoids to thrive in different climates, they could be used as biocontrol agents for *A. fraterculus* in almost all suitable areas for the establishment of the pest. Future studies using SDMs for each *A. fraterculus* morphotype can be

informative for designing control tactics at a local level in the Americas. The use of SDMs to predict the establishment of biocontrol agents and pests under climate change is informative and useful for designing pest management strategies.

Acknowledgements

We thank the two anonymous reviewers who helped with the improvement of the manuscript.

References

1. Prentice IC, Farquhar GD, Fasham MJR, Goulden ML, Heimann M, Jaramillo VJ, et al. The carbon cycle and atmospheric carbon dioxide. In: Climate change 2001: the scientific basis, Intergovernmental panel on climate change [Internet]. 2001 [cited 2024 Jan 23]. Available from: https://hal.science/hal-03333974

2. Rosenzweig C. Global cClimate cChange: Predictions and oObservations. Am J Agric Econ. 1989;71(5):1265–1271.

3. Field CB, Barros VR, Mastrandrea MD, Mach KJ, Abdrabo MAK, Adger WN, et al. Summary for pPolicymakers. In: Field CB, Barros VR, Dokken DJ, Mastrandrea MD, Bilir TE, Chatterjee M, et al., editors. Climate cChange 2014. Cambridge, United Kingdom, and New York, NY, USA: Cambridge University Press; 2015. pp. 1–32.

4. Bale JS, Masters GJ, Hodkinson ID, Awmack C, Bezemer TM, Brown VK, et al. Herbivory in global climate change research: direct effects of rising temperature on insect herbivores. Glob Change Biol. 2002;8(1): 1–16.

5. Hance T, van Baaren J, Vernon P, Boivin G. Impact of eExtreme tTemperatures on pParasitoids in a cClimate cChange pPerspective. Annu Rev Entomol. 2007;52(1): 107–126.

6. Wu LH, Hoffmann AA, Thomson LJ. Potential impact of climate change on parasitism efficiency of egg parasitoids: A meta-analysis of *Trichogramma* under variable climate conditions. Agric Ecosyst Environ. 2016;231: 143–155.

7. Furlong MJ, Zalucki MP. Climate change and biological control: the consequences of increasing temperatures on host–parasitoid interactions. Curr Opin Insect Sci. 2017; 20:39–44.

8. Chidawanyika F, Mudavanhu P, Nyamukondiwa C. Global cClimate cChange as a dDriver of bBottom-uUp and tTop-dDown fFactors in aAgricultural lLandscapes and the fFate of hHost-pParasitoid iInteractions. Front Ecol Evol. 2019;7.

9. Zhang YB, Zhang GF, Liu WX, Wan FH. Variable temperatures across different stages have novel effects on behavioral response and population viability in a host-feeding parasitoid. Sci Rep. 2019;9(1): 2202.

10. Srivastava V, Lafond V, Griess VC. Species distribution models (SDM): Aapplications, benefits and challenges in invasive species management. CABI Rev. 2019;2019: 1–13.

Formatted: Font: Italic

11. Gillson L, Dawson TP, Jack S, McGeoch MA. Accommodating climate change contingencies in conservation strategy. Trends Ecol Evol. 2013;28(3): 135–142.

12. Kumar S, Neven LG, Yee WL. Evaluating correlative and mechanistic niche models for assessing the risk of pest establishment. Ecosphere. 2014;5(7): art86.

13. Ørsted IV, Ørsted M. Species distribution models of the sSpotted wWing *Drosophila* (*Drosophila suzukii*, Diptera: Drosophilidae) in its native and invasive range reveal an ecological niche shift. J Appl Ecol. 2019;56(2): 423–435.

14. Boys WA, Siepielski AM, Smith BD, Patten MA, Bried JT. Predicting the distributions of regional endemic dragonflies using a combined model approach. Insect Conserv Divers. 2021;14(1): 52–66.

15. Srivastava V, Roe AD, Keena MA, Hamelin RC, Griess VC. Oh the places they'll go: improving species distribution modelling for invasive forest pests in an uncertain world. Biol Invasions. 2021;23(1): 297–349.

16. Wang CJ, Wang R, Yu CM, Dang XP, Sun WG, Li QF, et al. Risk assessment of insect pest expansion in alpine ecosystems under climate change. Pest Manag Sci. 2021;77(7): 3165–3178.

17. Fischbein D, Lantschner MV, Corley JC. Modelling the distribution of forest pest natural enemies across invaded areas: Towards understanding the influence of climate on parasitoid establishment success. Biol Control. 2019;132: 177–188.

Formatted: Font: Italic

Formatted: Font: Italic

18. Lantschner MV, de la Vega G, Corley JC. Predicting the distribution of harmful species and their natural enemies in agricultural, livestock and forestry systems: A~~a~~n overview. Int J Pest Manag. 2019;65(3): 190–206.

19. Zhao H, Yang N, Huang H, Shi J, Xian X, Wan F, et al. Integrating biogeographic approach into classical biological control: Assessing the climate matching and ecological niche overlap of two natural enemies against common ragweed in China. J Environ Manage. 2023;347: 119095.

20. Garcia FRM, Ovruski SM, Suárez L, Cancino J, Liburd OE. Biological control of Tephritid fruit flies in the Americas and Hawaii: A review of the use of parasitoids and predators. Insects. 2020;11(10): 662.

21. Hernández-Ortiz V, Gómez-Anaya JA, Sánchez A, McPheron BA, Aluja M. Morphometric analysis of Mexican and South American populations of the *Anastrepha fraterculus* complex (Diptera: Tephritidae) and recognition of a distinct Mexican morphotype. Bull Entomol Res. 2004 Dec;94(6): 487–499.

22. Hernández-Ortiz V, Bartolucci AF, Morales-Valles P, Frías D, Selivon D. Cryptic s~~S~~pecies of the *Anastrepha fraterculus* c~~C~~omplex (Diptera: Tephritidae): A m~~M~~ultivariate a~~A~~pproach for the r~~R~~ecognition of S~~S~~outh ~~American~~American m~~M~~orphotypes. Ann Entomol Soc Am. 2012;105(2): 305–318.

23. Hernández-Ortiz V, Canal NA, Salas JOT, Ruíz-Hurtado FM, Dzul-Cauich JF. Taxonomy and phenotypic relationships of the *Anastrepha fraterculus* complex in the Mesoamerican

Formatted: Font: Italic

Formatted: Font: Italic

Formatted: Font: Italic

and Pacific Neotropical dominions (Diptera, Tephritidae). ZooKeys. 2015 Nov 26;(540): 95–124.

24. Nava DE, Botton M. Bioecologia e controle de *Anastrepha fraterculus* e *Ceratitis capitata* em pessegueiro. Pelotas. Embrapa Clima Temperado, 2010 [cited 2024 Jan 26]; Available from: ~~-~~http://www.infoteca.cnptia.embrapa.br/infoteca/handle/doc/888672 ~~http://www.infoteca.cnptia.embrapa.br/handle/doc/889693~~

25. Zucchi RA, Moraes RCB de, Malavasi A, Adaime R, Nava DE. Moscas-das-frutas, suas plantas hospedeiras e parasitoides no Brasil: dados consolidados (2022). Moscas-das-~~-~~f~~F~~rutas n~~N~~o Brasil c~~C~~onhecimento b~~B~~ásico e~~E~~ a~~A~~plicado [Internet]. 2023 [cited 2024 Jan 26];2. Available from: https://repositorio.usp.br/item/003123905

26. Dias VS, Lucky A. South American f~~F~~ruit f~~F~~ly (suggested common name) *A~~a~~nastrepha fraterculus* (Wiedemann)(Insecta: Diptera: Tephritidae): EENY-696/IN1196, 12/2017. EDIS [Internet]. 2018 [cited 2024 Jan 26];2018(1). Available from: https://journals.flvc.org/edis/article/download/105365/126710

27. Castañeda M del R, Osorio F A, Canal NA, Galeano PÉ. Especies, distribución y hospederos del género *Anastrepha* Schiner en el departamento del Tolima, Colombia. Agron Colomb. 2010;28(2): 264–272.

28. Jaramillo MG, Machado PB, Sáenz JRR, Montoya D, Valencia FF, Pinzón JFT. Las moscas de las frutas de la zona central cafetera de Colombia y su clave ilustrada. Cenicafé. 2015;66(2):58–72.

Formatted: Font: Italic

Formatted: Font: Italic

Formatted: Not Highlight

Formatted: Font: Italic

Formatted: Font: Italic

29. ICA[Internet]. Plagas c~~C~~uarentenarias p~~P~~resentes [cited 2024 Jun 6]. Available from: https://www.ica.gov.co/areas/agricola/servicios/epidemiologia-agricola/plagas-reglamentadas/plagas-cuarentenarias-presentes.aspx

30. Hernández Calderón FA. Etapas de la erradicación y manejo integrado de la mosca de la fruta (*Ceratitis capitata* Wied) en la Región Ica. Thesis, Universidad Nacional Agraria la Molina. 2016. Available from: http://repositorio.lamolina.edu.pe/handle/20.500.12996/1783

31. Perez-Staples D, Diaz-Fleischer F, Montoya P, Vera MT. Area-w~~W~~ide m~~M~~anagement of f~~F~~ruit f~~F~~ly p~~P~~ests. CRC Press; 2019.

32. Montoya P, Cancino J, Zenil M, Santiago G, Gutierrez JM. The a~~A~~ugmentative b~~B~~iological control component in the ~~mexican~~ Mexican national campaign against *Anastrepha* spp. f~~F~~ruit f~~F~~lies. In: Vreysen MJB, Robinson AS, Hendrichs J, editors. Area-wide control of insect pests. Dordrecht: Springer Netherlands; 2007. pp. 661–670.

33. Paranhos BJ, Nava DE, Malavasi A. Biological control of fruit flies in Brazil. Pesqui Agropecuária Bras. 2019;54: e26037.

34. Dias NP, Montoya P, Nava DE. A 30-year systematic review reveals success in tephritid fruit fly biological control research. Entomol Exp Appl. 2022;170(5): 370–384.

35. Alvarado-Gálvez L, Medianero E. Especies de parasitoides asociados a moscas de la fruta del género *Anastrepha* (Diptera: Tephritidae) en Panamá, República de Panamá. Sci Panamá. 2015;25: 47–62.

Formatted: Font: Italic

Formatted: Font: Italic

Formatted: Font: Italic

36. Monteiro LB, Nishimura G, Monteiro RS. Natural parasitism in fruit fly (Diptera: Tephritidae) and interaction with wild hosts surrounding apple orchards adjacent to Atlantic Forest fragments in Paraná State, Brazil. Braz J Biol. 2021;83: e250505.

37. Nunes AM, Nava DE, Müller FA, Gonçalves R da S, Garcia MS. Biology and parasitic potential of *Doryctobracon areolatus* on *Anastrepha fraterculus* larvae. Pesqui Agropecuária Bras. 2011;46: 669–671.

38. Nava DE, Gonçalves R da S, Nornberg S, Scheunemann T, Grutzmacher A. Avaliação preliminar da seletividade de inseticidas e do parasitismo de *Doryctobracon areolatus* (Hymenoptera: Braconidae) em moscas-das-frutas. Empresa Brasileira de Pesquisa Agropecuária: Embrapa Clima Temperado, Ministério da Agricultura, Pecuária e Abastecimento. 2019, Boletin de Pesquisa e Desenvolvimiento, ISSN 1678-2518; 325.

39. Ovruski SM, Bezdjian LP, Nieuwenhove GAV, Albornoz-Medina P, Schliserman P. Host preference by *Diachasmimorpha longicaudata* (Hymneoptera: Braconidae) reared on larvae of *Anastrepha fraterculus* and *Ceratitis capitata* (Diptera: Tephritidae). Fla Entomol. 2011 Jun;94(2): 195–200.

40. Ovruski SM, Oroño LE, Schliserman P, Nuñez-Campero S. The effect of four fruit species on the parasitization rate of *Anastrepha fraterculus* (Diptera: Tephritidae, Trypetinae) by *Diachasmimorpha longicaudata* (Hymenoptera: Braconidae, Opiinae) under laboratory rearing conditions. Biocontrol Sci Technol. 2007;17(10): 1079–1085.

41. Governo federal do Brasil, Portaria DAS n 784, de 19 de abril 2023 (April 19, 2023).

42. Ovruski S, Aluja M, Sivinski J, Wharton R. Hymenopteran p~~P~~arasitoids on f~~F~~ruit-infesting Tephritidae (Diptera) in Latin America and the Southern United States: Diversity, d~~D~~istribution, t~~T~~axonomic s~~S~~tatus and their use in f~~F~~ruit f~~F~~ly b~~B~~iological c~~C~~ontrol. Integr Pest Manag Rev. 2000;5(2): 81–107.

43. Carvalho R da S. Avaliação das liberações inoculativas do parasitóide exótico *Diachasmimorpha longicaudata* (Ashmead)(Hymenoptera: Braconidae) em pomar diversificado em Conceição do Almeida, BA. Neotrop Entomol. 2005 Oct;34: 799–805.

44. Cancino J, Ruiz L, López E, Aguilar E, Galvez C, Montoya P, et al. Suppression of *Ceratitis capitata* (Wied.) (Diptera: Tephritidae) populations in coffee in the Mexico–Guatemala border region through the augmentative releases of *Diachasmimorpha longicaudata* (Ashmead) (Hymenoptera: Braconidae). Biocontrol Sci Technol. 2019 Aug 3;29(8): 822–826.

45. Ovruski SM, Colin C, Soria A, Oroño LE, Schliserman P. Introducción y producción en laboratorio de *Diachasmimorpha tryon~~-~~i* y *Diachasmimorpha longicaudata* (Hymenoptera: Braconidae) para el control biológico de *Ceratitis capitata* (Diptera: Tephritidae) en la Argentina. Rev Soc Entomológica Argent. 2003;62(3–4): 49–59.

46. Governo federal do Brasil, Instrução n~~N~~ormativa c~~C~~onjunta SDA/SMC n 2, de 29 de agosto de 2018. (Aug 29, 2018).

47. Walder JMM. Produção de moscas-das-frutas e seus inimigos naturais: associação de moscas estéreis e controle biológico. Controle Biológico no Brasil Parasitóides e Predadores. São Paulo, Manole. Brazil; 2002. p. 181–190.

Formatted: Font: Italic

Formatted: Font: Italic

Formatted: Font: Italic

Formatted: Font: Italic

Formatted: Font: Italic

Formatted: Font: Italic

48. Sugayama RL, Malavasi A. Anastrepha fraterculus (Wiedemann) (Diptera: Tephritidae) na região produtora de maçãs do Rio Grande do Sul: relação com seus inimigos naturais e potencial para controle biológico. Ph. D. Thesis, Universidade de São Paulo. 2000. Available from: https://repositorio.usp.br/item/001083555

49. Owens H, Barve V, Chamberlain S, Ram K, Hart T, rOpenSci (ropensci.org/). spocc: Interface to species occurrence data sources. 2023 [cited 2024 Feb 2]. Available from: https://cran.r-project.org/web/packages/spocc/index.html

50. Moreira G da C, Bosco NS, Shimbori EM, Sobral-Souza T, Onody HC. Synthesis of the knowledge of braconids in Brazil: list of species, data gaps and spatial biases in the sampling. [Preprint]. 2023 [cited 2024 Jun 21]. Available from: https://www.researchsquare.com/article/rs-2527415/v1

51. Hijmans RJ, Phillips S, Elith JL and J. dismo: Species distribution modeling. 2023 [cited 2024 Sep 8]. Available from: https://cran.r-project.org/web/packages/dismo/index.html

52. R Core Team. R: A l~~L~~anguage and e~~E~~nvironment for s~~S~~tatistical c~~C~~omputing. Vienna, Austria: R Foundation for Statistical Computing; 2023. Available from: https://www.R-project.org/

53. Barbet-Massin M, Jiguet F, Albert CH, Thuiller W. Selecting pseudo-absences for species distribution models: how, where and how many?. Methods Ecol Evol. 2012;3(2): 327–338.

54. Fick SE, Hijmans RJ. WorldClim 2: new 1-km spatial resolution climate surfaces for global land areas. Int J Climatol. 2017;37(12): 4302–4315.

55. O'Neill BC, Tebaldi C, Van Vuuren DP, Eyring V, Friedlingstein P, Hurtt G, et al. The scenario model intercomparison project (ScenarioMIP) for CMIP6. Geosci Model Dev. 2016;9(9): 3461–3482.

56. Thomson AM, Calvin KV, Smith SJ, Kyle GP, Volke A, Patel P, et al. RCP4.5: a pathway for stabilization of radiative forcing by 2100. Clim Change. 2011;109(1): 77.

57. Riahi K, Rao S, Krey V, Cho C, Chirkov V, Fischer G, et al. RCP 8.5—A scenario of comparatively high greenhouse gas emissions. Clim Change. 2011;109(1): 33.

58. Graham MH. Confronting multicollinearity in ecological multiple regression. Ecology. 2003;84(11): 2809–2815.

59. Dormann CF, Elith J, Bacher S, Buchmann C, Carl G, Carré G, et al. Collinearity: a review of methods to deal with it and a simulation study evaluating their performance. Ecography. 2013;36(1): 27–46.

60. Naimi B, Hamm NAS, Groen TA, Skidmore AK, Toxopeus AG. Where is positional uncertainty a problem for species distribution modelling?. Ecography. 2014;37(2): 191–203.

61. Mi C, Huettmann F, Guo Y, Han X, Wen L. Why choose Random Forest to predict rare species distribution with few samples in large undersampled areas? Three Asian crane species models provide supporting evidence. PeerJ. 2017 ;5: e2849.

62. Luan J, Zhang C, Xu B, Xue Y, Ren Y. The predictive performances of random forest models with limited sample size and different species traits. Fish Res. 2020;227: 105534.

63. Li Z, Ye Z, Wan R, Zhang C. Model selection between traditional and popular methods for standardizing catch rates of target species: A case study of Japanese Spanish mackerel in the gillnet fishery. Fish Res. 2015;161: 312–319.

64. Breiman L. Random fForests. Mach Learn. 2001;45(1): 5–32.

65. Hastie T, Tibshirani R, Friedman J. Random Forests. In: Hastie T, Tibshirani R, Friedman J, editors. The elements of statistical learning: Data mining, inference, and prediction. New York, NY: Springer; 2009. pp. 587–604.

66. Probst P, Wright MN, Boulesteix A. Hyperparameters and tuning strategies for random forest. WIREs Data Min Knowl Discov. 2019;9(3): e1301.

67. Probst P, Boulesteix AL, Bischl B. Tunability: Importance of hyperparameters of machine learning algorithms. J Mach Learn Res. 2019;20(53): 1–32.

68. Kuhn M. A Short Introduction to the caret Package. R Found Stat Comput. 2015;1: 1–10.

69. Robin X, Turck N, Hainard A, Tiberti N, Lisacek F, Sanchez JC, et al. pROC: an open-source package for R and S+ to analyze and compare ROC curves. BMC Bioinformatics. 2011;12(1): 77.

70. Hijmans RJ, Bivand R, Pebesma E, Sumner MD. terra: Spatial data analysis. 2024 [cited 2024 Mar 24]. Available from: https://cran.r-project.org/web/packages/terra/index.html

71. Massicotte P, South A, Hufkens K. rnaturalearth: World map data from natural earth. 2023 [cited 2025 Jan 7]. Available from: https://cran.r-project.org/web/packages/rnaturalearth/index.html

72. Fielding AH, Bell JF. A review of methods for the assessment of prediction errors in conservation presence/absence models. Environ Conserv. 1997;24(1): 38–49.

73. Araújo MB, Pearson RG, Thuiller W, Erhard M. Validation of species–climate impact models under climate change. Glob Change Biol. 2005;11(9): 1504–1513.

74. Galvão-Silva FL, Araújo AS, Dias VS, do Nascimento AS, Joachim-Bravo IS. Responses of two *Anastrepha* species' immature stages infesting preferential hosts to different temperature exposures. Neotrop Entomol. 2024; 53: 342-350.

75. Santos W da S. Zoneamento ecológico de *Anastrepha fraterculus* e *Ceratitis capitata* (Diptera: Tephritidae) em dois cenários climáticos no Brasil. Ph. D. Thesis. Universidade de São Paulo. 2008. Available from: http://www.teses.usp.br/teses/disponiveis/11/11146/tde-12062008-161926/

76. Machado Teixeira C, Peter Krüger A, Nava DE, Mello Garcia FR. Global potential distribution of *Anastrepha grandis* (Diptera, Tephritidae) under climate change scenarios. Crop Prot. 2022;151: 105836.

77. Skendžić S, Zovko M, Živković IP, Lešić V, Lemić D. The impact of climate change on agricultural insect pests. Insects. 2021;12(5): 440.

78. Beck HE, McVicar TR, Vergopolan N, Berg A, Lutsko NJ, Dufour A, et al. High-resolution (1 km) Köppen-Geiger maps for 1901–2099 based on constrained CMIP6 projections. Sci Data. 2023;10(1): 724.

Formatted: Font: Italic

Formatted: Font: Italic

Formatted: Font: Italic

Formatted: Font: Italic

79. Morellato LPC, Haddad CFB. Introduction: The ~~brazilian~~ Brazilian ~~atlantic~~Atlantic forest1. Biotropica. 2000;32(4): 786.

80. Selivon D, Perondini ALP, Morgante JS. A genetic–morphological characterization of Two cryptic species of the *Anastrepha fraterculus* complex (Diptera: Tephritidae). Ann Entomol Soc Am. 2005;98(3): 367–381.

81. Selivon D, Perondini ALP, Hernández-Ortiz V, doVal FC, Camacho A, Gomes FR, et al. Genetical, morphological, behavioral, and ecological traits support the existence of three ~~brazilian~~ Brazilian species of the *Anastrepha fraterculus* complex of cryptic species. Front Ecol Evol. 2022;10.

82. Rubel F, Kottek M. Observed and projected climate shifts 1901-2100 depicted by world maps of the Köppen-Geiger climate classification. Meteorol Z. 2010;19(2): 135.

83. Araujo ES, Monteiro LB, Monteiro RS, Nishimura G, Franck P, Lavigne C. Impact of native forest remnants and wild host plants on the abundance of the South American fruit fly, *Anastrepha fraterculus* in Brazilian apple orchards. Agric Ecosyst Environ. 2019;275: 93–99.

84. Ramos Peña ÁM, Yábar Landa E, Ramos Peña JC, Ramos Peña ÁM, Yábar Landa E, Ramos Peña JC. Diversidad, fluctuación poblacional y hospedantes de moscas de la fruta *Anastrepha* spp. y *Ceratitis capitata* (Wiedemann) (Diptera: Tephritidae) en el Valle de Abancay, Apurímac, Perú. Acta Zool Mex. 2019;35.

85. Salazar-Mendoza P, Peralta-Aragón I, Romero-Rivas L, Salamanca J, Rodriguez-Saona C. The abundance and diversity of fruit flies and their parasitoids change with elevation in

Formatted: Font: Italic

Formatted: Font: Italic

Formatted: Font: Italic

Formatted: Font: Italic

guava orchards in a tropical ~~Andean forest~~Andean Forest of Peru, independent of seasonality. PLOS ONE. 2021;16(4): e0250731.

86. Hernández-Ortiz V, Barradas-Juanz N, Díaz-Castelazo C. A Review of the natural host plants of the *Anastrepha fraterculus* complex in the Americas. In: Area-wide management of fruit fly pests. CRC Press; 2019.

87. Mendoza RG. Contribución al estudio de las moscas Anastrephas en Colombia. Rev Fac Nac Agron Medellín. 1952;12(42): 423–549.

88. Nùñez Bueno L. Contribución al reconocimiento de las moscas de las frutas (Diptera: Tephriridae) en Colombia. Rev ICA. 1981;(4): 173–179.

89. Rust EW. Anastrepha fraterculus Wied.(Trypetidae)—A severe menace to the southern United States. J Econ Entomol. 1918;11(6): 457–467.

90. Núñez-Campero SR, González C, Rull J, Ovruski SM. Maximum E~~E~~ntropy (MaxEnt) as extreme distribution indicator of two Neotropical fruit fly parasitoids in irrigated drylands of Argentina. Bull Entomol Res. 2022;112(5): 636–645.

91. da Silva Gonçalves R, Nunes AM, Poncio S, Manica-Berto R, Nörnberg SD, Grützmacher AD, et al. Bionomics, thermal requirements, and life table of the fruit fly parasitoid *Doryctobracon areolatus* (Hymenoptera: Braconidae) under various thermal regimes. Biol Control. 2018;127: 101–108.

92. Coley PD. Possible effects of climate change on plant/herbivore interactions in moist tropical forests. Clim Change. 1998;39(2): 455–472.

Formatted: Font: Italic

Formatted: Font: Italic

93. Jahnke SM, Ponte EMD, Redaelli LR, Rego DRGP. Spatial patterns and associations of *Anastrepha fraterculus* (Diptera: Tephritidae) and its parasitoid *Doryctobracon areolatus* (Hymenoptera: Braconidae) in organic orchards of *Psidium guajava* and *Acca sellowiana*. Fla Entomol. 2014;97(2): 744–752.

94. Piovesan B, Gonçalves R da S, Nörnberg SD, Llano JHG, Contreras-Miranda J, Bernardi D, et al. Spatial and temporal dispersion of *Doryctobracon areolatus* (Szépligeti) (Hymenoptera: Braconidae) in orchards infested with *Anastrepha fraterculus* (Wiedemann) (Diptera: Tephritidae). Environ Entomol. 2023;52(4): 565–573.

95. Ovruski SM, Schliserman P, Oroño LE, Nuñéz-Campero SR, Albornoz-Medina P, Bezdjian LP, et al. Natural ~~ocurrence~~occurrence of Hymenopterous parasitoids associated with *Anastrepha fraterculus* (Diptera: Tephritidae) in Myrtaceae species in Entre Rios, Northeastern Argentina. Fla Entomol. 2008;91(2): 220–227.

96. ~~Del Medio Ambiente, P.G., del~~ Banco Mundial~~, G~~. Tomando impulso en la agricultura Peruana: oportunidades para aumentar la productividad y mejorar la competitividad del sector. 2017 [cited 2024 Jun 18]. Available from: https://documents.worldbank.org/en/publication/documents-reports/documentdetail/781561519138355286/Gaining-momentum-in-Peruvian-agriculture-opportunities-to-increase-productivity-and-enhance-competitiveness

97. Salazar-Mendoza P, Peralta-Aragón I, Romero Rivas L. Análisis faunístico, fluctuación poblacional y parasitoides asociados a las moscas de la fruta (Diptera: Tephritidae) en el níspero. Rev Perú Entomol. 2018 Dec 1;53: 21–32.

98. Carrejo NS, O. RG. Parasitoids reared from species of *Anastrepha* (Diptera: Tephritidae) in Valle del Cauca, Colombia. Fla Entomol. 1999;82(1): 113–118.

99. Saavedra-Díaz J, Galeano-Olaya PE, Canal D NA. Relaciones ecológicas entre frutos hospederos, moscas frugívoras y parasitoides en un fragmento de bosque seco tropical. Rev Cienc Agríc. 2017;34(1): 32–49.

100. Montoya P, Ayala A, López P, Cancino J, Cabrera H, Cruz J, et al. Natural parasitism in fruit fly (Diptera: Tephritidae) populations in disturbed areas adjacent to commercial mango orchards in Chiapas and Veracruz, Mexico. Environ Entomol. 2016;45(2): 328–337.

101. Sivinski J, Piñero J, Aluja M. The distributions of parasitoids (Hymenoptera) of *Anastrepha* fruit flies (Diptera: Tephritidae) along an altitudinal gradient in Veracruz, Mexico. Biol Control. 2000;18(3): 258–269.

102. Baranowski RM, Swanson RW. Introduction of *Parachasma* (=*Opius*) *Cereus* (Hymenoptera: Braconidae) into ~~florida~~ Florida as a parasite of *Anastrepha suspensa* (Diptera: Tephritidae). Fla Entomol. 1970;53(3): 161–162.

103. Eitam A, Sivinski J, Holler T, Aluja M. Biogeography of braconid parasitoids of the ~~caribbean~~ Caribbean fruit fly (Diptera: Tephritidae) in Florida. Ann Entomol Soc Am. 2004;97(5): 928–939.

104. Meirelles RN, Redaelli LR, Ourique CB. Thermal requirements and annual number of generations of *Diachasmimorpha longicaudata* (Hymenoptera: Braconidae) reared in the S~~s~~outh ~~american~~American fruit fly and the ~~mediterranean~~ Mediterranean fruit fly (Diptera: Tephritidae). Fla Entomol. 2015;98(4): 1223–1226.

Formatted: Font: Italic

Formatted: Font: Italic

Formatted: Font: Italic

Formatted: Font: Italic

Formatted: Font: Italic

Formatted: Font: Italic

105. Harbi A, Beitia F, Chermiti B, de Pedro L, Ferrara F, Asís JD, et al. Abiotic factors affecting *Diachasmimorpha longicaudata* (Hymenoptera: Braconidae) activity as a natural enemy of *Ceratitis capitata* (Diptera: Tephritidae) under semi-natural conditions in the Mediterranean region. J Appl Entomol. 2018;142(8): 755–764.

106. Ndlela S, Azrag AGA, Mohamed SA. Determination of temperature thresholds for the parasitoid *Diachasmimorpha longicaudata* (Hymenoptera: Braconidae), using life cycle simulation modeling: Implications for effective field releases in classical biological control of fruit flies. PLOS ONE. 2021;16(8): e0255582.

107. Soares GKA, Fidelis EG, Farias ES, Rodrigues GS, Paes JLA. Range expansion and population dynamics of *Bactrocera carambolae* in Roraima, Brazil. Crop Prot. 2023;165: 106167.

108. Carvalho RS. Estudos de laboratório e de campo com o parasitóide exótico *Diachasmimorpha longicaudata* Ashmead (Hymenoptera. Braconidae. no Brasil. Ph.D. Thesis, Universidade de São Paulo. 2003.

109. Aluja M, Guillen J, Liedo P, Cabrera M, Rios E, de la Rosa G, et al. Fruit infesting tTephritids [Dipt.: Tephritidae] and associated parasitoids in Chiapas, Mexico. Entomophaga. 1990;35(1): 39–48.

110. Montoya P, Liedo P, Benrey B, Cancino J, Barrera JF, Sivinski J, et al. Biological cControl of *Anastrepha* spp. (Diptera: Tephritidae) in mMango oOrchards through aAugmentative rReleases of *Diachasmimorpha longicaudata* (Ashmead) (Hymenoptera: Braconidae). Biol Control. 2000;18(3): 216–224.

Formatted: Font: Italic

Formatted: Font: Italic

Formatted: Font: Italic

Formatted: Font: Italic

111. Montoya P, López P, Cruz J, López F, Cadena C, Cancino J, et al. Effect of *Diachasmimorpha longicaudata* releases on the native parasitoid guild attacking *Anastreph*a spp. larvae in disturbed zones of Chiapas, Mexico. BioControl.;62(5):581–593.

112. Baranowski R, Glenn H, Sivinski J. Biological control of the ~~caribbean~~Caribbean fruit fly (Diptera: Tephritidae). Fla Entomol. 1993;76(2): 245–251.

Formatted: Font: Italic

Formatted: Font: Italic

Supporting information

S1 Fig. Overlapping suitable predicted areas for *A. fraterculus* and *D. areolatus* in the future. Overlapping suitable areas were predicted for *A. fraterculus* and *D. areolatus* under two future climatic scenarios (SSP2-4.5 and SSP5-8.5) and two time periods (2021-2040 and 2041-2060).

S2 Fig. Overlapping suitable predicted areas for *A. fraterculus* and *D. longicaudata* in the future. Overlapping suitable areas were predicted for *A. fraterculus* and *D. longicaudata* under two future climatic scenarios (SSP2-4.5 and SSP5-8.5) and two time periods (2021-2040 and 2041-2060).

S1 Table. Global Climate Models (GCM) from the Coupled Model Intercomparison Project Phase 6 (CMIP6) used in our RF model construction.

S2 Table. Extension of suitable and unsuitable areas predicted for the pest, *A. fraterculus* and the parasitoids *D. areolatus* and *D. longicaudata* across the Americas.

Response to reviewers

Editor comments:

Thank you for submitting your manuscript to PLOS ONE. After careful consideration, we feel that it has merit but does not fully meet PLOS ONE's publication criteria as it currently stands. Therefore, we invite you to submit a revised version of the manuscript that addresses the points raised during the review process.

This is a much improved version, however, the ms still needs additional improvements. Please careful edit your ms to remove grammatical error and improve other parts (e.g. quotation of references cited). Comments of both reviewers should be carefully considered and addressed. Authors may also consider to further elaborate either on maps or in the text on the expected fruit crops that would come under attack due to climate change.

Reply: We appreciate the editor's observations. The grammatical errors in the manuscript have been corrected, and the references have been revised to comply with the journal's requirements. Regarding the fruit crops potentially affected by the expansion of *A. fraterculus*, we mentioned some of these crops in the manuscript. However, we acknowledge that this pest has over 160 host species. To address this, we have added a paragraph at the end of the first section of the discussion, emphasizing the importance of focusing research on crops in high-risk regions.

Review Comments to the Author:

1. Reviewer #1: General comments:

The manuscript has been improved but grammatical errors remain at the following lines and should be corrected before publication.

Line 50- should be "Therefore, increases in mean temperature, fewer occurrences of freezing temperatures"

Response: The sentence was corrected (line 52)

Line 68- should be “This approach may help entomologists to predict”.

Response: The sentence was corrected (line 71)

Line 79- “MASL (meters above sea level)”.

Response: The sentence was corrected (line 83)

Line 110 delete “in the future”

Response: The sentence was corrected (line 123)

Line 398 delete “While” or use a comma instead of a period.

Response: The sentence was corrected (line 446)

Line 41 possibly should be “in the U.S. and Mexico”

Response: Maybe the Line wasn’t written right by the reviewer, because do not match the line 41 with the suggestion.

Line 533 delete While” as in line 398.

Response: The sentence was corrected (line 623)

Line 581 “Florida. The parasitoid reduced”

Response: The sentence was corrected (line 690)

We sincerely appreciate the time and careful attention put into reviewing our manuscript. We have implemented all the grammatical corrections you identified,

2. Reviewer #3: General comments:

The manuscript entitled “Biological control under climate change: Distribution patterns of the South American fruit fly, Anastrepha fraterculus and two of its parasitoids in the Americas” uses species distribution models (SDM) to predict the potential geographic range of a pest, its biological control agents, and the areas that overlap between the two.

The manuscript is clear and acceptable for publication. Here are some minor suggestions or comments:

Introduction comments: The three hypotheses (L113 – 119) are interesting but are never discussed further.
Reply: In response to the reviewer's comment, we have added dedicated discussion paragraphs after each relevant section to explicitly address and evaluate the three hypotheses. See lines 525-534, 609-617, and 704-709.

Materials and methods comments: (L123-130) How many occurrences per species were found and used to build the SDM analysis? Are you sure you can find occurrences of all three species in the Vertebrate Network? Have you sorted or selected the occurrences (according to their reliability, for example)?
Reply: The number of occurrences used in the model’s construction has been added to the methods section (lines 143-148). Regarding the vertebrate network database, no records were initially available for the species in this database. To obtain these records, we used the “occ” function from the “spocc” package in RStudio, following the code provided by its authors, and included all recommended databases. The occurrences were then filtered to remove duplicates. Due to the limited number of available records for the parasitoids, we retained all occurrences in the final model construction.

Results comments: In general: Occupation zones are described by countries, regions (Amazon

region, Mesoamerican countries, Orinoquia region....) and geological formations (Andes mountains). These names are never indicated on any map, which makes them difficult for non-specialist readers to understand.

Reply: We appreciate the suggestion. While we did not label specific zones (e.g., countries, regions like the Amazon or Orinoquía, or geological formations like the Andes) on the maps, we intentionally described them using cardinal directions (e.g., northern, southern) within each country or area. This approach was taken to emphasize the significance of these regions in relation to pest expansion and to guide research focus accordingly. However, we acknowledge that adding geographical labels could improve clarity for non-specialist readers, and we will consider this for future work.

Other comments: L247-251: "the largest extension of suitable areas". This paragraph requires clarification. You mention "largest extension of suitable areas", but it is unclear whether the values refer to current or future occupancy or an extension factor. From Table 2, it looks like occupancy between 2041 and 2060 according to the SS95-8.5 scenario, although not all the values are identical.

Reply: The paragraph has been modified to specify that the values refer to projected occupancy under the SSP5-8.5 scenario (2041-2060). See lines 273-277

Fig 1 legend: L291: Remove "Extension of".

Reply: "Extension of" has been removed from the Fig 1 legend (Line 330)

Fig 2 legend: L297: Change "(B-D)" by (B-E). Idem for fig 3 and fig 5.

The figure references have been corrected to (B-E) in Figs 2, 3, and 5 legends. (lines 336, 370, 429).

Discussion comments: The three main parts of the discussion contain a substantial amount of information. It may be advisable to conclude each section with a summary of the key points (a take-home message).

Reply: We appreciate the reviewer's suggestion. In response, we have added concise summary

statements at the end of each discussion section to highlight the key findings and main conclusions of our study.

Bibliography comments: In some refs, each word is capitalized. Species names are not always italicized.

Reply: Regarding our bibliography formatting, we have systematically addressed these issues by:

- Standardizing title capitalization to sentence case for all references
- Ensuring proper italicization of all species names throughout the bibliography

These corrections have been implemented across the entire reference section to comply with the journal's style guidelines and maintain proper formatting standards.

We extend our sincere gratitude to the reviewers for their insightful feedback, which has been important in refining this work. We deeply appreciate the careful attention given to our manuscript; each constructive comment has helped strengthen our arguments and enhance clarity. The manuscript has significantly improved through the revision process, and we are truly thankful for the expertise shared throughout the review.
