# Supplementary material for: Biological control under climate change: Distribution patterns of the South American fruit fly, Anastrepha fraterculus and two of its parasitoids in the Americas
Source: PLoS One. 2025 Jun 11;20(6):e0325761. doi: 10.1371/journal.pone.0325761 (PMC12157005; doi:10.1371/journal.pone.0325761)
Supplement: Attachment — Submitted filename: Response to Reviewers.docx [file pone.0325761.s006.docx]

**Rebutal Letter**

**Edittor comments:**

The paper contains useful information following an interesting approach. However, evaluating the ms myself I m rather aligned with Reviewer #1. Without pointing into details at the current stages, I found the Results and Discussion section difficult to follow, wordy and with details that can not be followed. Also, data presentation should be reconsidered and become more comprehensive. Apparently, this needs to be thoroughly revised. Also, I would suggest to separate Results from discussion. Also, to improve the ms comments of both reviewers, especially those of Reviewer 1, should be carefully addressed. The list o References cited should be also throughly edited to align with the style of Plos One.

**Reply:**

We appreciate the observations, we separate the Results and Discussion sections to facilite the compression, also we considerate to include two new figures to be more comprehensive, and the references were edited to aling with Plos one style

**Journal Requirements:**

1. Please ensure that your manuscript meets PLOS ONE's style requirements, including those for file naming. The PLOS ONE style templates can be found at
2. We note that the grant information you provided in the ‘Funding Information’ and ‘Financial Disclosure’ sections do not match. When you resubmit, please ensure that you provide the correct grant numbers for the awards you received for your study in the ‘Funding Information’ section.
3. Thank you for stating the following in the Acknowledgments Section of your manuscript: [We thank the anonymous reviewers who helped with the improvement of the manuscript, and the Coordination for the Improvement of Higher Education Personnel – Brazil (CAPES) by financial support. The author D.E. Nava is thankful for the financial support of Conselho Nacional de Desenvolvimento Científico e Tecnológico – CNPq, #310233/2020-8] We note that you have provided funding information that is not currently declared in your Funding Statement. However, funding information should not appear in the Acknowledgments section or other areas of your manuscript. We will only publish funding information present in the Funding Statement section of the online submission form. Please remove any funding-related text from the manuscript and let us know how you would like to update your Funding Statement. Currently, your Funding Statement reads as follows: [The authors received no specific funding for this work.] Please include your amended statements within your cover letter; we will change the online submission form on your behalf.
4. We note that [Figures 2-4] in your submission contain [map/satellite] images which may be copyrighted. All PLOS content is published under the Creative Commons Attribution License (CC BY 4.0), which means that the manuscript, images, and Supporting Information files will be freely available online, and any third party is permitted to access, download, copy, distribute, and use these materials in any way, even commercially, with proper attribution. For these reasons, we cannot publish previously copyrighted maps or satellite images created using proprietary data, such as Google software (Google Maps, Street View, and Earth). For more information, see our copyright guidelines: <http://journals.plos.org/plosone/s/licenses-and-copyright>.
5. Please include captions for your Supporting Information files at the end of your manuscript, and update any in-text citations to match accordingly. Please see our Supporting Information guidelines for more information: [http://journals.plos.org/plosone/s/supporting-information](http://track.editorialmanager.com/CL0/http:%2F%2Fjournals.plos.org%2Fplosone%2Fs%2Fsupporting-information/1/010f019361b8f549-758ad435-a1a9-436b-a4c4-25b9cf3c9b22-000000/hewY-cWGdPvofR5PfOmTCgrrVpUfj70d--fkEsLikIA=186).

**Reply:**

1. We have updated the style of the paper to comply with the PLOS ONE formatting guidelines.
2. Changes have been made to sections 2 and 3 of the manuscript. Additionally, we have resubmitted the 'Funding Information' and 'Financial Disclosure' sections as requested.
3. The maps were created using RStudio with the rnaturalearth package. The methodology has been revised to clarify this process.
4. The supporting information captions were include at the end of the manuscript.

**Review Comments to the Author**

Reviewer #1: The reviewer found the manuscript to be timely and of considerable general interest. Here are some minor comments.
References 1-5 are outdated and newer articles with more information about recent climate change are readily available and free. It would be preferable to keep these but also add 5 newer articles to flesh out this part of the introduction.

**Reply:** Changes in references were made

Line 88 “agent against such species”

**Reply:** We have corrected the English throughout the manuscript.

Lines 261-266. The reviewer had difficulty sorting out what was meant by relative contribution because the number of individuals comprising each individual code is not well specified. Perhaps information could be added to Table 1 about this.

**Reply:** The Relative Variable Importance ranks predictors based on their impact on model performance, showing how each predictor contributes to improvements when splits are made across the entire forest

Line 288. Should this be Fig. or Figure? At first, the reviewer could not find Figure 2.

**Reply:** Changes were made

Line 365 Reference [88]. Steck et al 1980, Proc Entomol Soc. Washington, is a more recent reference to presence in the US.

**Reply:** No recent references have been found regarding the presence of the species in the United States.

**Reviewer #2: General comments:**

The manuscript is not written well and need intensive English editing (language rules, syntax, preposition words). In addition, there are many redundant arguments within text which make reading awkward.
Due to the general scope of the manuscript, its better to present data with tables and graphs. The results section contains mass textual descriptions of future regional distributions within Brazil, which are not common for the global reader and cannot be accessible in the presented maps. It's suggested to use geographical and climatic characteristics (i.e., arid, temperate, high altitude etc') instead.
The authors describe in details the expected distribution changes within countries. The general phenomena are increase in all three species distribution which allow to keep biological control activities. The authors however do not elaborate the potential biological responses due to the significant differences between the two parasitoied species (general increase of 10-13% with A. fraterculus, 4-8% with D. longicaudata and 22-38% with D. arealotus upon the different model scenarios). As authors cite, there are already evident from the field on the effects of climate change on the focal species. If the model may contribute to the potential activity of A. fraterculus parasitoids, its expected to discusses it thorough.

**Reply**: The manuscript has undergone English editing, and the Results and Discussion sections have been separated. The climatic characteristics used in the manuscript are accessible. The inclusion of the Basil regions was necessary because, in our study, Brasil was the country with the largest suitable areas for the pest. Regarding the increase in suitable areas mentioned by the reviewer, we believe the extension of suitable areas is more important for predicting locations where biological control programs can be implemented.

Examples:
Introduction:
Row 49: its better to delete " and their natural enemies" , since you use (row 51) "Similarly" and describe the effect on natural enemies.
Row 52: " than on insect pests themselves" its not clear why you repeat on what is written before (Row 49)
Row 63: " Currently, many SDMs are being developed using correlative models" repeat previous argument (row 60).
Row 64: unlike the "SDMs" MaxEnt and Bioclim, "Random forest" is a machine learning algorithm and not a species distribution Model per se.
Row 68: the author claims that " Currently, the focus of SDMs is not only to understand the effects of climate change on agricultural pests…." Need to be justified by the developers of MaxEnt and Bioclim which are not been credited within this manuscript.

**Reply:** We have corrected the English throughout the manuscript.

Methods:
The author used random pseudo-absences locations where it is assumed that there is no record or presence of the species, thus providing a point of comparison with the presence records of each species with a ratio of 2:1 pseudo-absence to presence locations generation. Its important to understand 1. what is the spatial scale from which those pseudo locations were chosen (may affect model outcomes)? 2. What is the rational for choosing ratio of 2:1 pseudo-absence to presence locations?

**Reply:**

1. "The scale of the pseudo-absences was set at 2.5 arc-minutes.
2. Typically, the minimum number of pseudo-absences used in random forest species distribution models (SDM) is at least equal to the number of occurrences. It is also recommended to use 10 replications when there are fewer than 10,000 occurrences. To improve the accuracy of our predictions, we increased the number of pseudo-absences and conducted at least 10 replications. The AUC values of our models indicate that our predictions are accurate.

Results
Using previous findings (Selivon et al) of different distribution of three Brazilian morphotypes, the authors argue that " our results suggest that the expansion of suitable areas for A. fraterculus could be associated with specific adaptations of morphotypes.". since the model is generalized for the 10 mentioned A. fraterculus morphotypes, this link needs at least supporting evident.

**Reply:**

This misunderstanding arose during the writing process. We have corrected the English to better reflect our results. What we intended to convey is that our study evaluates the species *A. fraterculus* without the different morphotypes.
