# Supplementary material for: Biological control under climate change: Distribution patterns of the South American fruit fly, Anastrepha fraterculus and two of its parasitoids in the Americas
Source: PLoS One. 2025 Jun 11;20(6):e0325761. doi: 10.1371/journal.pone.0325761 (PMC12157005; doi:10.1371/journal.pone.0325761)
Supplement: Attachment — Submitted filename: Response to Reviewers .docx [file pone.0325761.s008.docx]

**Response to reviewers**

**Editor comments:**

Thank you for submitting your manuscript to PLOS ONE. After careful consideration, we feel that it has merit but does not fully meet PLOS ONE’s publication criteria as it currently stands. Therefore, we invite you to submit a revised version of the manuscript that addresses the points raised during the review process.

This is a much improved version, however, the ms still needs additional improvements. Please careful edit your ms to remove grammatical error and improve other parts (e.g. quotation of references cited). Comments of both reviewers should be carefully considered and addressed. Authors may also consider to further elaborate either on maps or in the text on the expected fruit crops that would come under attack due to climate change.

**Reply:** We appreciate the editor’s observations. The grammatical errors in the manuscript have been corrected, and the references have been revised to comply with the journal’s requirements. Regarding the fruit crops potentially affected by the expansion of *A. fraterculus*, we mentioned some of these crops in the manuscript. However, we acknowledge that this pest has over 160 host species. To address this, we have added a paragraph at the end of the first section of the discussion, emphasizing the importance of focusing research on crops in high-risk regions.

**Review Comments to the Author:**

1. **Reviewer #1: General comments:**

The manuscript has been improved but grammatical errors remain at the following lines and should be corrected before publication.

Line 50- should be “Therefore, increases in mean temperature, fewer occurrences of freezing temperatures”

**Response:** The sentence was corrected (line 52)

Line 68- should be “This approach may help entomologists to predict”.

**Response:** The sentence was corrected (line 71)

Line 79- “MASL (meters above sea level)”.

**Response:** The sentence was corrected (line 83)

Line 110 delete “in the future”

**Response:** The sentence was corrected (line 123)

Line 398 delete “While” or use a comma instead of a period.

**Response:** The sentence was corrected (line 446)

Line 41 possibly should be “in the U.S. and Mexico”

**Response:** Maybe the Line wasn’t written right by the reviewer, because do not match the line 41 with the suggestion.

Line 533 delete While” as in line 398.

**Response:** The sentence was corrected (line 623)

Line 581 “Florida. The parasitoid reduced”

**Response:** The sentence was corrected (line 690)

We sincerely appreciate the time and careful attention put into reviewing our manuscript. We have implemented all the grammatical corrections you identified,

2. **Reviewer #3: General comments:**

The manuscript entitled “Biological control under climate change: Distribution patterns of the South American fruit fly, Anastrepha fraterculus and two of its parasitoids in the Americas” uses species distribution models (SDM) to predict the potential geographic range of a pest, its biological control agents, and the areas that overlap between the two.

The manuscript is clear and acceptable for publication. Here are some minor suggestions or comments:

**Introduction comments**: The three hypotheses (L113 – 119) are interesting but are never discussed further.

**Reply**: In response to the reviewer's comment, we have added dedicated discussion paragraphs after each relevant section to explicitly address and evaluate the three hypotheses. See lines 525-534, 609-617, and 704-709.

**Materials and methods comments**: (L123-130) How many occurrences per species were found and used to build the SDM analysis? Are you sure you can find occurrences of all three species in the Vertebrate Network? Have you sorted or selected the occurrences (according to their reliability, for example)?

**Reply**: The number of occurrences used in the model’s construction has been added to the methods section (lines 143-148). Regarding the vertebrate network database, no records were initially available for the species in this database. To obtain these records, we used the “occ” function from the “spocc” package in RStudio, following the code provided by its authors, and included all recommended databases. The occurrences were then filtered to remove duplicates. Due to the limited number of available records for the parasitoids, we retained all occurrences in the final model construction.

**Results comments:** In general: Occupation zones are described by countries, regions (Amazon region, Mesoamerican countries, Orinoquia region....) and geological formations (Andes mountains). These names are never indicated on any map, which makes them difficult for non-specialist readers to understand.

**Reply**: We appreciate the suggestion. While we did not label specific zones (e.g., countries, regions like the Amazon or Orinoquía, or geological formations like the Andes) on the maps, we intentionally described them using cardinal directions (e.g., northern, southern) within each country or area. This approach was taken to emphasize the significance of these regions in relation to pest expansion and to guide research focus accordingly. However, we acknowledge that adding geographical labels could improve clarity for non-specialist readers, and we will consider this for future work.

**Other comments**: L247-251: “the largest extension of suitable areas”. This paragraph requires clarification. You mention "largest extension of suitable areas", but it is unclear whether the values refer to current or future occupancy or an extension factor. From Table 2, it looks like occupancy between 2041 and 2060 according to the SS95-8.5 scenario, although not all the values are identical.

**Reply**: The paragraph has been modified to specify that the values refer to projected occupancy under the SSP5-8.5 scenario (2041-2060). See lines 273-277

Fig 1 legend: L291: Remove “Extension of”.

**Reply:** "Extension of" has been removed from the Fig 1 legend (Line 330)

Fig 2 legend: L297: Change “(B-D)” by (B-E). Idem for fig 3 and fig 5.

The figure references have been corrected to (B-E) in Figs 2, 3, and 5 legends. (lines 336, 370, 429).

**Discussion comments**: The three main parts of the discussion contain a substantial amount of information. It may be advisable to conclude each section with a summary of the key points (a take-home message).
**Reply:** We appreciate the reviewer's suggestion. In response, we have added concise summary statements at the end of each discussion section to highlight the key findings and main conclusions of our study.

**Bibliography comments**: In some refs, each word is capitalized. Species names are not always italicized.

**Reply:** Regarding our bibliography formatting, we have systematically addressed these issues by:

- Standardizing title capitalization to sentence case for all references
- Ensuring proper italicization of all species names throughout the bibliography

These corrections have been implemented across the entire reference section to comply with the journal's style guidelines and maintain proper formatting standards.

We extend our sincere gratitude to the reviewers for their insightful feedback, which has been important in refining this work. We deeply appreciate the careful attention given to our manuscript; each constructive comment has helped strengthen our arguments and enhance clarity. The manuscript has significantly improved through the revision process, and we are truly thankful for the expertise shared throughout the review.
